# Enterovirus Inhibition by Hinged Aromatic Compounds with Polynuclei

**DOI:** 10.3390/molecules25173821

**Published:** 2020-08-22

**Authors:** Jih Ru Hwu, Avijit Panja, Srinivasan Jayakumar, Shwu-Chen Tsay, Kui-Thong Tan, Wen-Chieh Huang, Yu-Chen Hu, Pieter Leyssen, Johan Neyts

**Affiliations:** 1 Department of Chemistry, National Tsing Hua University, Hsinchu 300, Taiwan; panjanthu2016@gmail.com (A.P.); vasan6328@gmail.com (S.J.); tsay.susan@gmail.com (S.-C.T.); kttan@mx.nthu.edu.tw (K.-T.T.); jordanback2001@yahoo.com.tw (W.-C.H.); 2 Frontier Research Center on Fundamental and Applied Sciences of Matters, National Tsing Hua University, Hsinchu 300, Taiwan; ychu@mx.nthu.edu.tw; 3 Department of Chemistry, National Central University, Jhongli City, Taoyuan 320, Taiwan; 4 Department of Chemical Engineering, National Tsing Hua University, Hsinchu 300, Taiwan; 5 Rega Institute for Medical Research, Katholieke Universiteit Leuven, Minderbroedersstraat 10, Leuven B-3000, Belgium; Pieter.Leyssen@kuleuven.be

**Keywords:** enterovirus, morpholine, furan, thiophene, pyrazole, hinged bond

## Abstract

The modern world has no available drugs for the treatment of enteroviruses (EV), which affect millions of people worldwide each year. The EV71 is a major causative disease for hand, foot, and mouth disease; sometimes it is associated with severe central nervous system diseases. Treatment for enteroviral infection is mainly supportive; treatment for aseptic meningitis caused by enteroviruses is also generally symptomatic. Upon the urgent request of new anti-enterovirus drugs, a series of hinged aromatic compounds with polynulei were synthesized through two different chemical pathways. Among these morpholine–furan/thiophene/pyrrole–benzene–pyrazole conjugates, three new agents exhibited inhibitory activity with EC_50_ = 2.29–6.16 μM toward EV71 strain BrCr in RD cells. Their selectivity index values were reached as high as 33.4. Their structure–activity relationship was deduced that a thiophene derivative with morpholine and trifluorobenzene rings showed the greatest antiviral activity, with EC_50_ = 2.29 μM.

## 1. Introduction

Millions of people worldwide each year are affected by enteroviruses (EV), which are often found in the respiratory secretions and stools of an infected person [1]. These viruses cause blurred vision, encephalitis, pericarditis, rashes, and summer colds. Enteroviruses are by far the most common causes of aseptic meningitis in children. For example, EV is responsible for 30,000–50,000 meningitis hospitalizations per year as a result of 30–50 million infections in the United States. There are 64 non-polio EV that can cause disease in humans [2]. Infection can result in a wide variety of symptoms, including acute flaccid paralysis, acute hemorrhagic conjunctivitis, aseptic meningitis, hand/foot/mouth disease, myocarditis, neonatal sepsis-like disease, respiratory illness (common cold) [1]. Enterovirus 71 is notorious and associated with severe central nervous system diseases [3].

Enteroviruses are members of the picornavirus family, a large and diverse group of small RNA viruses associated with several human and mammalian diseases. These viruses are characterized by a single positive-strand genomic RNA. The current treatment for enteroviral infection is mainly supportive; for aseptic meningitis is still symptomatic. The treatment consists of prevention and complications for patients with enteroviral carditis [4]. Neither approved vaccines nor existing drugs are available nowadays [5]. The goal of this work is to develop new compounds as drug leads with impressive selective index toward enteroviruses.

In 2015, Pourianfar and Grollo [6] reviewed scientists’ numerous efforts on the discovery of synthetic and natural compounds with activity against EV71 infection. Liu et al. [7] recorded many compounds isolated from biogenic sources with anti-EV71 activity over the period between 2005–2015. Recently reported anti-EV agents include aminopyridyl 1,2,5-thiadiazolidine 1,1-dioxides [8], 3-aryl-1,2,4-oxadiazoles [7], benzothiophenes [9], *N*^6^-benzyladenosine [10], 3-benzyl-1,3-benz-oxazine-2,4-diones [11], biaryl-substituted quinolones [12], diarylhydrazides [13], disaccharide heparan sulfates [5], epidithiodiketopiperazines [14], isopentenyladenosine [15], α-keto amides [16,17], nitrobenzonitriles [18], peptidomimetic aldehydes [19], quinoline–thiophene conjugates [20], sophoridine alkaloid [21], stilbenoids [22], multi-tryptophan derivatives [23], and others.

Some hinged aromatic compounds show significant anti-EV71 activity. They include conjugates of aniline–oxazolopyridine [24], benzene–heterocycle [25], benzene–oxadiazole [26], pyridine–quinolone–arene [27], pyrazolopyridine–thiophene [28], etc. In 2019, Egorova et al. [29] summarized recent developments of hinged capsid-binding inhibitors of EV71.

**Figure 1:**
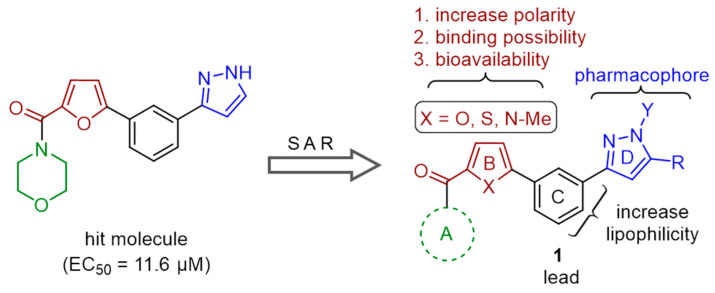
Design of new hinged compounds **1** to inhibit enterovirus.

In the past, our laboratories have also reported hinged conjugates with antiviral activities. Bisbenzimidazole–pyridine conjugates inhibit pestivirus replication in RNA polymerase [30]. Biphenylimidazopyridines exhibit activity against bovine viral diarrhoea virus [31]. Coumarin–benzimidazole conjugates and their ribofuranosides inhibit HCV [32]. Imidazole–isoquinoline conjugates act as enterovirus replication inhibitor TTP-8307 [33]. In addition to those compounds, Neyts’ research group collected many others from various sources. After high throughput screening of these compounds against the EV71 target, the hinged furan–benzene–pyrazole conjugate shown below was found with potency of EC_50_ = 11.6 µM. We considered it as a hit molecule and, accordingly, designed and synthesized a series of hinged heterocycle–benzene–pyrazole conjugated compounds **1** (Figure 1). Their role of becoming drug leads would be on the basis of their inhibitory activity toward EV71 to be tested and evaluated.

The presence of an oxygen, sulfur, or nitrogen atom in the B ring of target molecules **1** would increase polarity and their potential to form hydrogen bonding with the target virus. It could also improve bioavailability and the pharmacokinetic characteristics of the conjugated molecules [34]. The furan nucleus exists in many natural coumarins, flavonoids, terpenoids, etc. with significant biological activities. The presence of a thiophene unit in drugs may alter the metabolic pathway and thus thiophene derivatives may have less toxic effects or better therapeutic profile [35]. It also functions as a potential site of metabolism. Pyrrole derivatives exhibit a wide range of biological activities. They become antibacterial, anticancer, antifungal, anti-inflammatory, and antiviral agents [36]. Incorporation of a central benzene moiety as the C ring is to increase their lipophilicity [37]. Pyrazole moiety (i.e., the D ring in targets **1**) exists in some drugs, such as betazole, celecoxib, difenamizole, fezolamide, and rimonabant [38]. Moreover, many antibacterial, anti-inflammatory, antifungal, antileishmanial, antitumoral, antiviral, and analgesic agents [39] contain the pyrazole moiety. Pyrazole derivatives also exhibit a broad spectrum of drug-like properties [40] and show important bioactivity.

Herein we report our new findings on hinged heterocyclic aromatic compounds showing activity against EV71. A series of new compounds were synthesized by two different chemical pathways; three of which exhibited inhibitory activity with EC_50_ = 2.29–6.16 μM toward EV71 strain BrCr in RD cells. Their structure–activity relationship is also elucidated.

## 2. Results

### 2.1. Chemical Synthesis by Direct Meerwein Arylation of Heteroarenes (Method 1, Scheme 1)

Coupling of the commercially available ethyl 2-furoate (**2**) with 3-aminobenzaldehyde (**3**) was performed in the presence of sodium nitrite, copper (I) chloride, and hydrochloric acid at 0–25 °C. The hinged intermediate **4** was obtained in 32% yield, which is comparable with those reported by Aoyama et al. [41]. Treatment of the aldehyde **4** with tosyl hydrazine and then trimethylsilyl acetylene (**5**) gave the tricyclic pyrazole **6** at 95 °C in 85% yield by a single-flask reaction [42]. After removal of the silyl group by *n*-tetrabutylammonium flouride (TBAF), the resultant ester **7** was saponificated with aqueous LiOH in THF to afford heterotricycle **8** in 78% yield.

Amidation of the common heterotricylic 2-furoatic acid **8** was accomplished through their activation with 1-ethyl-3-(3-dimethylaminopropyl)carbodiimide (EDCI), hydroxybenzotriazole (HOBt), and *N*,*N*-diisopropylethylamine (DIPEA). Then the intermediate was allowed to react with cyclic amines **9** or arylamines **11** in DMF. Accordingly, the desired targets **10a**,**b**,**c** and **12a**,**b** with polynuclei were obtained in 85–90% yields, respectively. Secondary cyclopropylamide **13** and (methoxyethyl)amide **14** were also prepared by the same method for the analysis of the structure–activity relationship. The relative low yields were obtained in the key step **2** + **3** → **4** in Method 1 for the formation of the strategic carbon–carbon bonds. Thus the second method was applied for production of the desired targets in sufficient quantities for biological tests. 

### 2.2. Chemical Synthesis by Palladium-Catalyzed Cross-Coupling Reaction Involving C–H Activation (Method 2, Scheme 2)

Condensation of 2-furan, 2-thiophene, and 2-pyrrole carboxylic acids **15a**,**b**,**c** with morpholine in the presence of EDCI, HOBt, and DIPEA in DMF at room temperature generated the corresponding amides **16a**,**b**,**c**, respectively, in high yields. The next step was to arylate the furan-, thiophene-, and pyrrole-containing amides **16a**,**b**,**c** at their C-5 position. Often the arylation might take place randomly at the C-2, -3, -4, and -5 positions [43]. After many trials, we found that the difficulty on regioselective arylation of Ring B at their C-5 position could be circumvented by using the method reported by Doucet et al. [44], in which their substrates are furan esters. The new examples in our synthesis shown in Scheme 2 were furan amides. Accordingly, the formation of the strategic carbon–carbon bonds was achieved by reaction of the morpholine amides **16a**,**b**,**c** with 3-bromobenzaldehyde (**17**) in the presence of palladium(II) acetate as the catalyst. Through oxidative addition and reductive elimination at 130 °C, the aldehydic carbonyl group well survived. It led to the desired hinged trinuclear benzaldehydes **18a**,**b**,**c** in 80–85% [44].

Regarding amide substrates **16a**,**b**,**c**, the use of palladium(II) acetate was essential for their formation of a C–C bond with benzaldehyde **17**. However, use of other palladium(II) catalysts, such as [1,1′-bis(diphenylphosphino)ferrocene]dichloropalladium(II), 1,4-bis(diphenylphosphino)-butanedichloropalladium(II), and bis(triphenylphosphine) dichloridepalladium(II) would lead to decarbonylative products through cross-coupling at the amide functionality. These results found by us echoed what Szostak et al. have reported very recently [45].

For the formation of the Ring D (i.e., pyrazole) in structure **1**, trinuclear benzaldehydes **18a**,**b**,**c** were condensed with various ketones **19** in methanolic NaOH at room temperature. α,β-Unsaturated ketones **20a**–**j** were generated in ~30% yields through aldol condensation. The lower yields obtained than expected came from the competitive self-condensation of reagents **19**. The terminal methyl group therein did not create significant steric hindrance to retard the enolates generated from the same ketones attacking the ketonic carbonyl group. Further condensation between enones **20a**–**j** with tosyl hydrazine followed by annulation produced the corresponding pyrazoles **21a**–**j** in 70–75% yields.

**Scheme 1:**
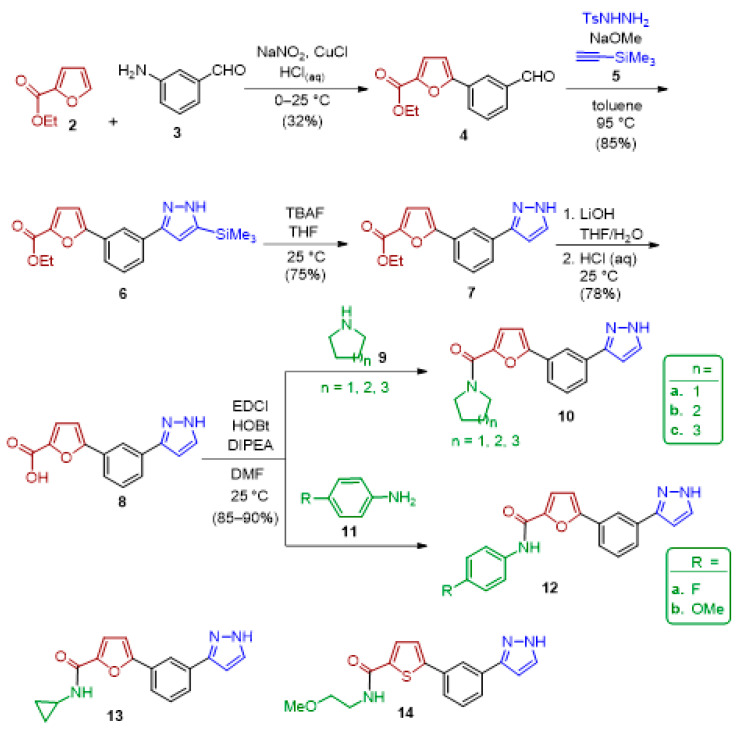
Synthesis of hinged aromatic compounds **10**, **12**, **13**, and **14** through a direct Meerwein arylation of heteroarenes.

**Scheme 2:**
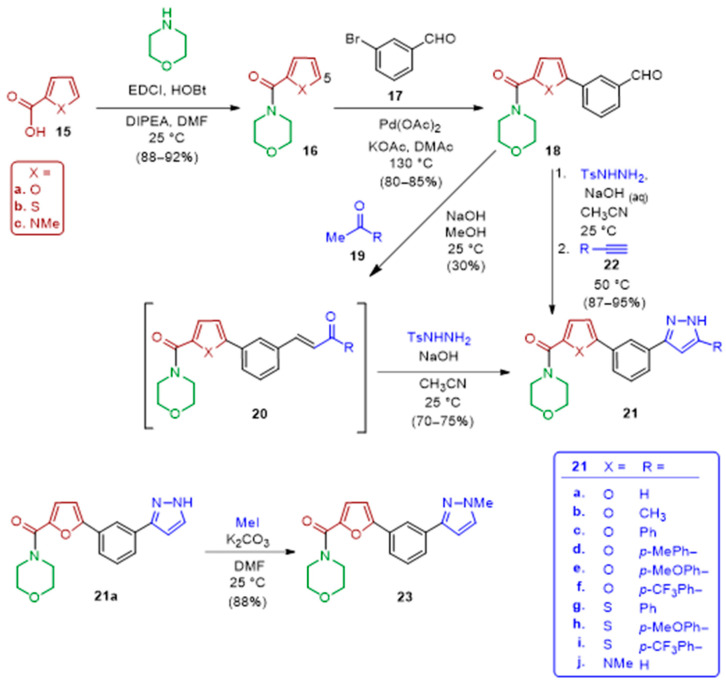
Synthesis of hinged aromatic compounds **21** through a palladium-catalyzed cross-coupling reaction involving C–H activation.

To improve the overall yields for the conversions of **18** → **20** → **21**, we applied the similar reaction conditions used for the conversion of aldehyde **4** to **8** by using terminal alkynes **22** (cf. (trimethylsilyl)acetylene in Scheme 1). Accordingly, the benzaldehyde intermediates **18a**,**b**,**c** were readily converted to the target pyrazoles **21a**–**j** in 87–95% yields through a condensation–cyclization process as shown in Scheme 2. Furthermore, the free N–H proton in target **21a** were readily converted to a methyl group in pyrazole **23** for the purpose of elucidation of structure–activity relationship.

### 2.3. Structural Identification of New Conjugated Compounds

The structures of all new compounds were confirmed on the basis of their spectroscopic characteristics. For example, the exact mass of **21g** was measured as 415.1353 for M^+^, which is very close to its theoretical value of 415.1354 (C_24_H_21_N_3_O_2_S)^+^. Its ^13^C-NMR spectrum showed 20 peaks, which matches exactly the total number of unsymmetrical carbons therein. The peak at 162.6 ppm corresponded to the C=O carbon. The two distinct peaks at 147.2 and 98.9 ppm came from the C=N and NNC*C*H carbons, respectively. Two peaks at 66.1 and 45.5 ppm were associated with the OCH_2_ and NCH_2_ carbons of the morpholine ring. Its ^1^H-NMR spectrum included two singlets at 7.81 and 6.60 ppm, which corresponded to the CH protons in the benzene and pyrazole rings, respectively. A multiplet appeared between 3.48–3.44 ppm for the protons in the morpholine ring. Its IR spectrum exhibited a weak absorption at 3167 cm^–1^ for the N–H stretching in the pyrazole ring and 2921 cm^–1^ for an aromatic C–H stretching. A strong characteristic absorption band at 1606 cm^–1^ was associated with the amido C=O stretching vibration.

**Figure 2:**
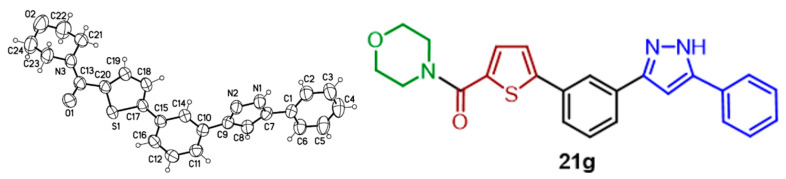
ORTEP diagram of compound **21g** (on the left side) obtained by X-ray analysis.

The structures of all new compounds were identified on the basis of their spectroscopic characteristics (see Supplementary Materials). Moreover, the polynuclear molecular framework of morpholine–thiophene–benzene–pyrazole–benzene conjugate **21g** was determined by single X-ray diffraction analysis (Figure 2). The ORTEP diagram shown in Figure 1 confirms the connection of the thiophene unit at its C-5 position to the benzene nucleus at its C-3 position through a hinged bond. It also indicates the presence of a pyrazole as the D ring. Its monoclinic crystals (mp 239.6–241.8 °C, ethanol) had the space group C2/c with a = 27.0531(13) Å, b = 6.0926(3) Å, c = 26.4787(13) Å, α = 90°, β = 109.8440(10)°, and γ = 90°.

### 2.4. Measurement of Lipophilicity

The log *P* values associated with the molecular lipophilicity of all hinged aromatic compounds with polynuclei were obtained by use of the ‘‘shake–flask method’’ [46]. The partition coefficient was measured as the ratio of the equilibrium concentrations of a dissolved hinged compound in a two-phase system consisting of *n*-octane and water, which are immiscible solvents. The temperature was kept constant at 25 ± 1 °C. The analytical method employed was UV spectrometry. As listed in Table 1, the log *P* values fell into the range of 2.56–5.04 for compounds **10a**–**c**, **12a**,**12b**, **13**, **14**, **21a**–**j**, and **23**.

As shown in Table 1, the concentrations of hinged aromatic compounds that inhibited virus replication by 50% (i.e., EC_50_) were calculated on the basis of the obtained dose-response curves. The concentrations to reduce host cell metabolism by 50% (i.e., CC_50_) were obtained for compounds that exhibited significantly low EC_50_ values. The selectivity index (SI = CC_50_/EC_50_), a measure for the therapeutic window of the compound in the assay system, was then calculated. The antiviral effect of hinged aromatic compounds that adversely affected the host cell metabolism was likely as a result of a pleiotropic or non-specific effect on the host cell. Among these 18 synthetic compounds, we found that the new hinged arenes **10c**, **21h**, and **21i** exhibited compelling potency in EV71 RD cells with EC_50_ values ranging from 2.29 to 6.16 μM. They displayed a significant window of selectivity with SI values between 10.2 and 33.4.

## 3. Discussion

### 3.1. Chemical Syntheses and Physical Properties

Application of Method 1 shown in Scheme 1 allowed us to produce polynuclear hinged compounds **10**, **12**, **13**, and **14** in five steps. Nevertheless, the first step would generate a diazonium salt intermediate from 3-aminobenzaldehyde (**3**) and sodium nitrite. Although being immediately consumed by ethyl 2-furoate (**2**) in situ, the violent decomposition hazard and the potential explosive property of the diazonium salt material brought a safety concern. By contrast, Method 2 shown in Scheme 2 provided a safe way to generate polynuclear hinged compounds, such as morpholine–furan/thiophene/pyrrole–benzene–pyrazoles **21**, in a large quantity through only three steps. The overall yields of the Method 2 (61–72%) were found ~3.8 times higher than those of the Method 1 (16–19%).

**Scheme 3:**
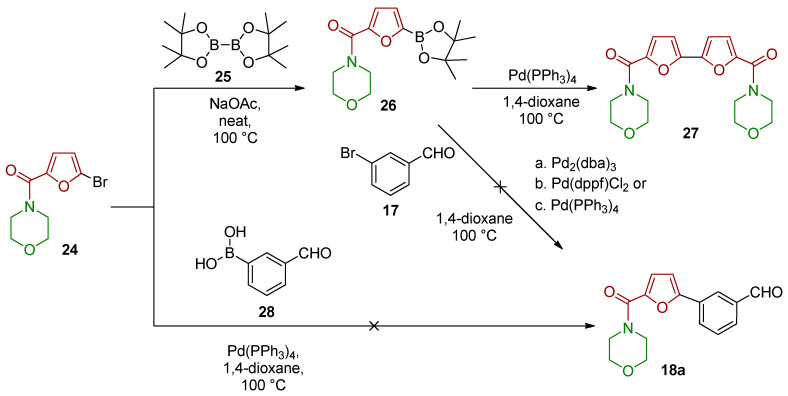
Competing reactions for the formation of the strategic hinged carbon–carbon bond.

For the formation of the strategic hinged carbon–carbon bond, different synthetic approaches were explored as shown in Scheme 3. 5-Bromofuranyl amide **24** was used as the starting material to ensure the regioselective bond formation occurring at the C-5 position. After it reacted with bis(pinacolato)diboron (**25**), the corresponding borate **26** was isolated exclusively. Nevertheless, to make an attempt to couple it with 3-bromobenzaldehyde (**17**) met with failure by use of three different palladium(II) catalysts, including Pd_2_(dba)_3_, Pd(dppf)Cl_2_, and Pd(PPh_3_)_4_. Instead of the target **18a**, the dimer **27** was formed as the major product in 60% yield. The target **18a** was not generated either by reaction of 5-bromofuranyl amide **24** with boronic acid **28** in the presence of Pd(PPh_3_)_4_ and dioxane/water. Our results shown in Scheme 3 indicate that organic oxyborane reagents (i.e., **25** and **28**) had stronger activity in favor of self-condensation through the homocoupling than the cross coupling with an aromatic bromides (e.g., **17**) through the Suzuki–Miyaura reaction.

### 3.2. Lipophilicity

Our target compounds with structure **1** are similar to those reported before [37,47] yet two differences exist. First, the B ring of our targets is either furan, thiophene, or pyrrole; the reported compounds contain an imidazole nucleus. Second, the connecting bond between the rings B and C is a carbon–carbon bond in our targets, yet the reported compounds contain an amide bond. Thus we considered generating the strategic carbon–carbon single bond under mild reaction conditions through the Meerwein arylation as shown in the Method 1. It involved the use of diazonium salts as a coupling partner.

Our design on molecules with the skeleton **1** as potential anti-enterovirus agents would allow the synthesized hinged compounds containing mixed units of morpholine, furan/thiophene/pyrrole, benzene, and pyrazole. In order to obtain the hinged conjugates with different lipophilicity, various aliphatic and aromatic primary or secondary amines were used in the Ring A during our synthesis (Scheme 1). The log *P* values of target compounds **10a**–**c**, **12a**, **12b**, **13**, **14**, **21a**–**j**, and **23** were found between 2.56–5.04, which fall into the ideal range between –0.4 and 5.6 for drug-like molecules [48]. These results confirm the validity of our design of new drug leads on their lipophilicity by conjugation of these heterocyclic and aryl rings in one entity.

Analysis of the log *P* values towards the variation on the Ring A indicate that compounds with aliphatic rings (i.e., **10a**, **10b**, and **13** with log *P* values of 3.25–3.87) are lower than compounds with aromatic rings (i.e., **12a** and **12b** with log *P* values of 4.15–4.18). When the ring size of cycloamines increases, the log *P* value increases (i.e., **10a**, **10b**, and **10c** with log *P* values of 3.42, 3.87, and 4.22, respectively).

Furthermore, addition of a benzene unit as the Ring E onto compounds with the skeleton **1** increases the log *P* value from 2.60 and 2.87 (i.e., **21a** and **21b**) to 3.92–4.33 (i.e., **21c**–**f**). Attachment of a –CF_3_ group onto the benzene unit slightly increases the log *P* value of the hinged compounds. Examples include the comparison of furan derivatives **21c** (nor-CF_3_) with **21f** (with-CF_3_), of which the log *P* values were 3.92 and 4.33, respectively. Moreover, thiophene derivatives **21g** (nor-CF_3_) and **21i** (with-CF_3_) had the log *P* values of 4.61 and 5.04, respectively. Among all hinged conjugates, the log *P* value was 5.04 for the compound **21i**. It exhibits the most significant activity against enterovirus.

### 3.3. Structure–Activity Relationship 

**Figure 3:**
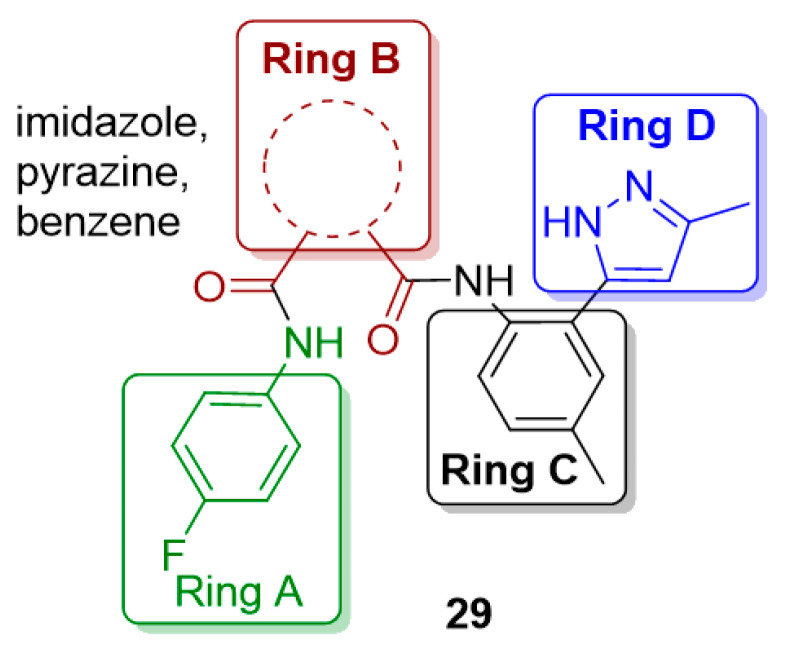
Structure of some conjugated compounds with anti-enteroviral activity.

Most of the hinged target compounds synthesized by us contain 4 or 5 rings. The order follows morpholine, cyclic amine, or arylamine as the Ring A; furan, thiophene, or pyrrole as the Ring B; disubstituted benzene as the Ring C; 1*H*-pyrazole as the Ring D; and benzene with certain electron-withdrawing or –donating groups as the Ring E if any. These new conjugated compounds are structurally close to those with the structure **29** reported previously. Nevertheless, it differs with ours with four characteristics: the first, Ring A is fixed as *p*-fluoroanilinyl moiety; the second, the Ring B is imidazole, pyrazine, or benzene; the third, the Ring E is absent; and the fourth, Ring B is connected with Ring C by an amide bond (cf. C–C bond in compounds **1**). Some of compounds **29** shown in Figure 3 show inhibitory activities against DENV and yellow fever virus, but not EV71.

According to the EC_50_, CC_50_, SI, and log *P* values of all compounds listed in Table 1, the following SAR guidelines are deduced for their activity against EV71. Among the 18 hinged aromatic compounds with polynuclei, compounds **10c**, **21h**, and **21i** exhibited greatest potency with EC_50_ values of 6.16, 4.10, and 2.29 μM, respectively; their SI values were 13.1, 10.2, and 33.4, respectively. A common feature shared by these compounds is that the ring B therein. It is a five-membered heterocyclic thiophene or furan. These moieties do not exist in the compounds **29** reported previously. Furthermore, the SI values of thiophene derivatives were found better than those of furan derivatives by factors of 6.8–12.8 (cf. **21h** versus **21e** and **21i** versus **21f**). These results are due to the lower toxicity (CC_50_ value) associated with thiophene derivatives **21h** and **21i** than the furan derivatives **21e** and **21f** by factors of 20.0–23.3.

Among the amide derivatives **10a**–**c** with four nuclei, compound **10a** with a five-membered ring A exhibited the lowest toxicity (CC_50_ = 119 μM), compound **10b** with a six-membered ring A exhibited the greatest potency (EC_50_ = 2.14 μM), yet compound **10c** with a seven-membered ring A exhibited the best selectivity (SI = 13.1). Among the three morpholine/thiophene derivatives **21g**,**h**,**i** with five nuclei, compound **21h** bearing an electron-donating –OMe group on the benzene ring E turned out to be more effective than the parent compound **21g** on the basis of the values of CC_50_, EC_50_, and SI. Nevertheless, the compound **21i** with an electron-withdrawing –CF_3_ group on the benzene ring E exhibited the lowest toxicity (CC_50_ = 76.4 μM), greatest potency (EC_50_ = 2.29 μM), and best selectivity (SI = 33.4). It also became the best candidate as the lead among all of the 18 hinged aromatic compounds with polynuclei.

Two different substituents were placed at the para position of the ring A anilinyl moiety. One was an electron-withdrawing –F group in compound **12a** and the other was an electron-donating –OMe group in compound **12b**. Their EC_50_ values were on the same order of micro molar (i.e., 1.73 μM and 2.41 μM); the selectivity of **12b** (SI = 8.70) was better than that of **12a** (SI = 2.60). Moreover, adjustment of lipophilicity of the conjugated target compounds were accomplished by use of different moieties as the ring A, including morpholine, heterocyclic amine, and aniline (see compounds in Table 1). Accordingly, various aliphatic and aromatic amines were used in the synthesis of compounds **10a**,**b**,**c** and **12a**,**b** shown in Scheme 1. Their lipophilicity fell within the range of 3.42–4.22.

On the other hand, removal of the N–H proton on the ring D by replacement with a –CH_3_ group did not improve the biological significance. Data of the related compounds **21a** and **23** are shown in Table 1.

## 4. Experimental Section

### 4.1. Assay of Antiviral Activity against of EV71 

The biological activity of the 18 hinged aromatic compounds against EV71 was evaluated in a cell-based assay with live enteroviruses. The EV71 strains were originated from Chang Gung University of Taiwan. The rhabdomyosarcoma (RD) cells, grown for 3–4 days in tissue culture flasks, were harvested and seeded in MEM Rega 3 medium (Gibco, Merelbeke, Belgium) supplemented with fetal bovine serum (FBS, 2.0%, Integro, Merelbeke, Belgium), 200 mM L-glutamine (Gibco, Merelbeke, Belgium), and aqueous 7.5% NaHCO_3_ (Gibco, Merelbeke, Belgium). The microtitre plates were incubated overnight to produce a non-confluent cell monolayer. Then assay plates were transferred to a liquid handling platform, on which a sample dilution series was prepared at a sample concentration of 0.100 mg/mL or lower in DMSO. After preparation of the hinged compound dilution series, virus was added to the wells with a dilution optimized for each EV71 strain. The final, maximal DMSO concentration that reached in the assay wells with the highest sample input (1.0%) was tolerated well by the cells. The assay plates were returned to the incubator at 37 °C (5.0% CO_2_) for four days. According to the manufacturers’ instructions (Promega, Leiden, The Netherlands), residual cell viability was then quantified by use of an MTS readout method. 

### 4.2. Chemical Syntheses and Structural Identification

All reactions were carried out in oven-dried glassware (120 °C) under an atmosphere of nitrogen unless as indicated otherwise. Acetonitrile, ethyl acetate, and hexanes from Mallinckrodt Chemicals Co. (Dublin, Ireland) were dried and distilled from CaH_2_. Dichloromethane and methanol were purchased from Mallinckrodt Chemicals Co. Tetrahydrofuran (THF) and toluene from Mallinckrodt Chemicals Co. were dried by distillation from sodium and benzophenone under an atmosphere of nitrogen. Dimethylacetamide (DMAc), dimethylformamide (DMF), *N*,*N*-diisopropylethylamine (DIPEA), and toluene were purchased from TEDIA. Cyclopropylamine, 4-fluoroaniline, 4-methoxyaniline, 2-methoxyethylamine, hydrochloric acid (HCl), lithium hydroxide (LiOH), potassium carbonate (K_2_CO_3_), and sodium hydroxide (NaOH) were purchased from Aldrich (St. Louis, MO, USA). 3-Bromobenzaldehyde, copper chloride, 2-furoic acid, 1-methyl-1*H* pyrrole-2-carboxylic acid, palladium(II)acetate Pd(OAc)_2_, potassium acetate (KOAc), sodium nitrite (NaNO_2_), and 2-thiophenecarboxylic acid were purchased from Alfa Aesar Chemical Co. (Ward Hill, MA, USA). 3-Aminobenzaldehyde, ethyl 2-furoate, sodium methoxide (NaOMe), trimethylsilylacetylene, and tetra-*n*-butylammonium fluoride (TBAF) were purchased from Merck (Kenilworth, NJ, USA). Azepane, 1-ethyl-3-(3-dimethylaminopropyl)carbodiimide hydrochloride (EDCI.HCl), hydroxybenzotriazole (HOBt), piperidine, and pyrrolidine were purchased from Tokyo Chemical Industry Co. Ltd. (Tokyo, Japan). 4-Ethynylanisole, methyl iodide (MeI), phenylacetylene, propyne, *p*-tolylacetylene, tosylhydrazine (TsNHNH_2_), and 4-(trifluoromethyl)phenylacetylene were purchased from Acros (Porto, Portugal).

Analytical thin layer chromatography (TLC) was performed on precoated plates (silica gel 60 F-254). Purification by gravity column chromatography was carried out by use of ultra-pure silica gel (particle size 40−63 μM, 230−400 mesh, Silicycle, Quebec, QC, Canada). Infrared spectra (IR) were measured on a Fourier transform infrared spectrometer (FT−IR, spectrum 100, PerkinElmer, Waltham, MA, USA). Absorption intensities are described by the following abbreviations: s, strong; m, medium; and w, weak. Proton NMR spectra were obtained on a 400 MHz spectrometer (Mercury-400, Varian, Palo Alto, CA, USA or AC-400, Bruker, Billerica, MA, USA) using chloroform-*d* (CDCl_3_) and dimethyl sulfoxide-*d_6_* (DMSO-*d_6_*) as the solvents. Proton NMR chemical shifts were referenced to the residual protonated solvent (*δ* 7.24 ppm for chloroform, 2.49 ppm for dimethyl sulfoxide). Carbon-13 NMR spectra were obtained on a 100 MHz spectrometer using chloroform-*d* (CDCl_3_) and dimethyl sulfoxide-*d_6_* (DMSO-*d_6_*) as the solvents (Mercury-400, Varian, Palo Alto, CA, USA or AC-400, Bruker, Billerica, MA, USA). Carbon-13 chemical shifts were referenced to the centre of the CDCl_3_ triplet (*δ* 77.0 ppm) or DMSO-*d_6_* septet (*δ* 39.5 ppm). Multiplicities are recorded by the following abbreviations: s, singlet; d, doublet; dd, doublet of doublet; t, triplet; q, quartet; m, multiplet; and *J*, coupling constant (hertz). High-resolution mass spectra (HRMS) were measured on an JMS-T100LP 4G instrument (JEOL, Tokyo, Japan) using a time-of-flight mass analyzer (TOF) with electrospray ionization (ESI).

#### 4.2.1. Ethyl 5-(3-Formylphenyl)furan-2-carboxylate (**4**)

To a solution containing 3-aminobenzaldehyde (**3**, 2.21 g, 18.2 mmol, 1.0 equiv) in aqueous HCl (2.0 N, 40 mL) was added ethyl 2-furoate (**2**, 2.56 g, 18.2 mmol, 1.0 equiv), sodium nitrite (1.26 g, 18.2 mmol, 1.0 equiv), and copper chloride (0.361 g, 3.65 mmol, 0.20 equiv). After the reaction mixture was stirred at 25 °C for 11 h, it was quenched with water (80 mL). The solution was extracted with EtOAc (3 × 30 mL) and the combined organic layers were dried over MgSO_4_ (s), filtered, and concentrated under reduced pressure to afford a residue. The residue was purified by use of column chromatography (10% EtOAc in hexanes as the eluent) to give the desired aldehyde **4** (1.42 g, 5.81 mmol) in 32% yield as white solids: TLC R*_f_* 0.45 (20% EtOAc as the eluent); mp (recrystallized from MeOH) 87.6−89.8 °C; ^1^H-NMR (CDCl_3_) *δ* 10.07 (s, 1 H, ArCHO), 8.26 (s, 1 H, ArH), 8.04 (d, *J* = 7.6 Hz, 1 H, ArH), 7.86 (d, *J* = 7.6 Hz, 1 H, ArH), 7.60 (t, *J* = 7.6 Hz, 1 H, ArH), 7.26 (d, *J* = 3.6 Hz, 1 H, furan), 6.85 (d, *J* = 3.6 Hz, 1 H, furan), 4.40 (q, *J* = 6.8 Hz, 2 H, CH_2_), 1.41 (t, *J* = 6.8 Hz, 3 H, CH_3_); ^13^C-NMR (CDCl_3_) *δ* 191.77 (CHO), 158.63 (C=O), 155.71, 136.81, 130.48, 130.23, 129.73, 129.63, 129.57, 125.78, 119.68, 107.93, 61.05 (CH_2_), 14.33 (CH_3_); IR (KBr pellet) 3087 (w), 2995 (m), 1700 (s, C=O), 1583 (m), 1454 (m), 1280 (m), 1021 (m), 786 (m) cm^−1^; HRMS (ESI-TOF) *m/z* [M + H]^+^ calcd for C_14_H_12_O_4_ + H 245.0816, found 245.0808.

#### 4.2.2. Ethyl 5-[3-(5-Trimethylsilyl-1*H*-pyrazol-3-yl)phenyl]furan-2-carboxylate (**6**)

To a solution containing benzaldehyde **4** (2.01 g, 8.23 mmol, 1.0 equiv) in toluene (30.5 mL) was added TsNHNH_2_ (1.84 g, 9.88 mmol, 1.2 equiv), sodium methoxide (2.22 g, 41.5 mmol, 5.0 equiv), and trimethylsilylacetylene (**5,** 2.42 g, 24.6 mmol, 3.0 equiv). After the reaction mixture was stirred at 95 ^o^C for 24 h, it was quenched with water (80 mL) and then extracted with EtOAc (3 × 30 mL). The combined organic layers were dried over MgSO_4_ (s), filtered, and concentrated under reduced pressure to afford a residue. The residue was purified by use of column chromatography (15% EtOAc in hexanes as the eluent) to give the desired pyrazole **6** (2.46 g, 6.97 mmol) in 85% yield as colorless oil: TLC R*_f_* 0.52 (30% EtOAc as the eluent); ^1^H-NMR (CDCl_3_) *δ* 8.21 (s, 1 H, ArH), 7.81 (d, *J* = 7.6 Hz, 1 H, ArH), 7.74 (d, *J* = 7.6 Hz, 1 H, ArH), 7.47 (t, *J* = 7.6 Hz, 1 H, ArH), 7.25 (d, *J* = 3.6 Hz, 1 H, furan), 6.80–6.79 (m, 2 H, furan + pyrazole), 4.40 (q, *J* = 6.8 Hz, 2 H, CH_2_), 1.40 (t, *J* = 6.8 Hz, 3 H, CH_3_), 0.35 (s, 9 H, Si(CH_3_)_3_); ^13^C-NMR (CDCl_3_) *δ* 158.91 (C=O), 157.50, 144.37 (C=N), 143.85, 134.00, 129.89, 129.13, 128.66, 126.41, 124.10, 122.25, 119.85, 110.13, 107.07, 60.89 (CH_2_), 14.39 (C*C*H_3_), −1.21 (SiCH_3_); IR (neat) 3324 (w, NH), 3176 (w), 2957 (w), 1708 (s, C=O), 1606 (w), 1441 (m), 1268 (s), 1101 (s), 842 (s) cm^−1^; HRMS (ESI-TOF) *m/z* [M + Na]^+^ calcd for C_19_H_22_N_2_O_3_Si + Na 377.1297, found 377.1222.

#### 4.2.3. Ethyl 5-[3-(1*H*-Pyrazol-3-yl)phenyl]furan-2-carboxylate (**7**)

To a solution containing pyrazole **6** (2.77 g, 7.82 mmol, 1.0 equiv) in THF (30 mL) was added TBAF (2.44 g, 9.38 mmol, 1.2 equiv). After the reaction mixture was stirred at 25 °C for 12 h, it was quenched with water (80 mL) and then extracted with EtOAc (3 × 30 mL). The combined organic layers were dried over MgSO_4_ (s), filtered, and concentrated under reduced pressure to afford a residue. The residue was purified by use of column chromatography (30% EtOAc in hexanes as the eluent) to give the desired pyrazole **7** (1.65 g, 5.84 mmol) in 75% yield as colorless oil: TLC R*_f_* 0.48 (60% EtOAc as the eluent); ^1^H-NMR (CDCl_3_) *δ* 8.12 (s, 1 H, ArH), 7.68 (d, *J* = 7.6 Hz, 2 H, 2 × ArH), 7.64 (d, *J* = 2.0 Hz, 1 H, pyrazole), 7.38 (t, *J* = 7.6 Hz, 1 H, ArH), 7.20 (d, *J* = 3.6 Hz, 1 H, furan), 6.70 (d, *J* = 3.6 Hz, 1 H, furan), 6.63 (d, *J* = 2.0 Hz, 1 H, pyrazole), 4.36 (q, *J* = 6.8 Hz, 2 H, CH_2_), 1.36 (t, *J* = 6.8 Hz, 3 H, CH_3_); ^13^C-NMR (CDCl_3_) *δ* 158.90 (C=O), 157.13, 149.0 (C=N), 143.85, 132.97, 129.91, 129.19, 126.28, 124.25, 124.10, 122.00, 119.84, 107.17, 102.88, 60.94 (CH_2_), 14.34 (CH_3_); IR (neat) 3291 (w, NH), 2925 (m), 1755 (s, C=O), 1466 (w), 1315 (s), 1106 (s), 1046 (s), 765 (w) cm^−1^; HRMS (ESI-TOF) *m/z* [M + H]^+^ calcd for C_16_H_14_N_2_O_3_ + H 283.1084, found 283.1077.

#### 4.2.4. 5-[3-(1*H*-Pyrazol-3-yl)phenyl]furan-2-carboxylic Acid (**8**)

In the first step, lithium hydroxide (0.266 g, 11.1 mmol, 1.5 equiv) was added to a solution containing ester **7** (2.09 g, 7.40 mmol, 1.0 equiv) in a mixture of THF and H_2_O (3:1) and the reaction mixture was stirred at 25 °C for 10 h. In the second step, the reaction mixture was acidified with aqueous HCl (1.5 N, 15 mL). The resultant was filtered and dried under reduced pressure over P_2_O_5_ to afford the acid **8** (2.33 g, 9.13 mmol) in 78% yield as white solids: TLC R*_f_* 0.46 (10% MeOH as the eluent); mp (recrystallized from MeOH) 234.2−236.2 °C; ^1^H-NMR (DMSO-*d_6_*) *δ* 8.21 (s, 1 H, ArH), 7.80 (d, *J* = 7.8 Hz, 1 H, ArH), 7.75 (d, *J* = 2.0 Hz, 1 H, pyrazole), 7.71 (d, *J* = 7.8 Hz, 1 H, ArH), 7.50 (t, *J* = 7.6 Hz, 1 H, ArH), 7.32 (d, *J* = 3.6 Hz, 1 H, furan), 7.20 (d, *J* = 3.6 Hz, 1 H, furan), 6.80 (d, *J* = 2.0 Hz, 1 H, pyrazole); ^13^C-NMR (DMSO-*d_6_*) *δ* 159.36 (C=O), 156.10, 147.67 (C=N), 144.38, 133.69, 132.16, 129.61, 129.50, 125.69, 123.37, 120.77, 119.76, 108.20, 102.23; IR (KBr pellet) 3321 (w, OH), 1713 (m, C=O), 1514 (w), 1495 (m), 1321 (s), 1292 (s), 901 (w), 765 (m), 607 (w) cm^−1^; HRMS (ESI-TOF) *m/z* [M + H]^+^ calcd for C_14_H_10_N_2_O_3_ + H 255.0771, found 255.0763.

### 4.3. Standard Procedure 1 for the Synthesis of Polycyclic Derivatives ***10a*, *10b*, *10c*, *12a*, *12b*, *13*** and ***14***

To a solution containing a furoic acid **8** (1.0 equiv) in DMF (5.0–6.5 mL) was added amine **9** or aniline **11** (1.2 equiv), EDCI (1.1 equiv), HOBt (1.1 equiv), and DIPEA (2.0 equiv). After the reaction mixture was stirred at 25 °C for 10–12 h, it was quenched with water (10 mL) and then extracted with EtOAc (3 × 15 mL). The combined organic layers were then dried over MgSO_4_ (s), filtered, and concentrated under reduced pressure to afford a residue. The residue was purified by use of column chromatography packed with silica gel to give the desired polycycle derivatives.

#### 4.3.1. (5-[3-(1*H*-Pyrazol-3-yl)phenyl]furan-2-yl)(pyrrolidin-1-yl)methanone (**10a**)

The standard procedure 1 was followed by use of furoic acid **8** (52.1 mg, 0.204 mmol, 1.0 equiv), pyrrolidine (**9a**, 17.4 mg, 0.245 mmol, 1.2 equiv), EDCI (34.8 mg, 0.224 mmol, 1.1 equiv), HOBt (30.2 mg, 0.224 mmol, 1.1 equiv), and DIPEA (52.7 mg, 0.408 mmol, 2.0 equiv). After the solution was stirred at 25 °C for 10 h and then worked up, the residue was purified by use of column chromatography (30% EtOAc in hexanes as the eluent) to give the pure polycycle **10a** (54.0 mg, 0.176 mmol) in 86% yield as colorless oil: TLC R*_f_* 0.53 (60% EtOAc as the eluent); ^1^H-NMR (CDCl_3_) *δ* 8.08 (s, 1 H, ArH), 7.66 (d, *J* = 8.0 Hz, 1 H, ArH), 7.62 (d, *J* = 2.2 Hz, pyrazole), 7.59 (d, *J* = 7.6 Hz, 1 H, ArH), 7.39 (t, *J* = 8.0 Hz, 1 H, ArH), 7.14 (d, *J* = 3.6 Hz, 1 H, furan), 6.72 (d, *J* = 3.6 Hz, 1 H, furan), 6.61 (d, *J* = 2.2 Hz, pyrazole), 3.89 (t, *J* = 6.4 Hz, 2 H, NCH_2_), 3.64 (t, *J* = 6.4 Hz, 2 H, NCH_2_), 2.00–1.97 (m, 2 H, CCH_2_C), 1.88–1.86 (m, 2 H, CCH_2_C); ^13^C-NMR (CDCl_3_) *δ* 158.11 (C=O), 155.13, 148.67 (C=N), 147.77, 132.95, 132.08, 130.34, 129.23, 125.80, 123.72, 121.56, 118.16, 106.96, 102.73, 47.18 (NCH_2_), 47.19 (NCH_2_), 26.66 (C*C*H_2_C), 23.70 (C*C*H_2_C); IR (neat) 3167 (m, NH), 2921 (m), 1606 (s, C=O), 1583 (m), 1433 (s), 1289 (w), 1080 (w), 792 (w), 741 (w) cm^−1^; HRMS (ESI-TOF) *m/z* [M]^+^ calcd for C_18_H_17_N_3_O_2_ 307.1320, found 307.1319.

#### 4.3.2. Piperidin-1-yl(5-[3-(1*H*-pyrazol-3-yl)phenyl]furan-2-yl)methanone (**10b**)

The standard procedure 1 was followed by use of furoic acid **8** (55.4 mg, 0.218 mmol, 1.0 equiv), piperidine (**9b**, 22.2 mg, 0.261 mmol, 1.2 equiv), EDCI (37.2 mg, 0.239 mmol, 1.1 equiv), HOBt (37.2 mg, 0.239 mmol, 1.1 equiv), and DIPEA (56.3 mg, 0.436 mmol, 2.0 equiv). After the solution was stirred at 25 °C for 11 h and then worked up, the residue was purified by use of column chromatography (35% EtOAc in hexanes as the eluent) to give the desired polycycle **10b** (63.0 mg, 0.196 mmol) in 90% yield as colorless oil: TLC R*_f_* 0.54 (70% EtOAc as the eluent); ^1^H-NMR (CDCl_3_) *δ* 8.06 (s, 1 H, ArH), 7.65 (d, *J* = 7.2 Hz, 1 H, ArH), 7.57–7.55 (m, 2 H, ArH + pyrazole), 7.36 (t, *J* = 7.2 Hz, 1 H, ArH), 6.99 (d, *J* = 3.6 Hz, 1 H, furan), 6.68 (d, *J* = 3.6 Hz, 1 H, furan), 6.59 (s, 1 H, pyrazole), 3.71–3.60 (m, 4 H, 2 × NCH_2_), 1.31–1.23 (m, 6 H, 3 × CCH_2_C); ^13^C-NMR (CDCl_3_) *δ* 159.10 (C=O), 154.55, 148.58 (C=N), 147.05, 132.88, 130.25, 129.16, 125.67, 123.91, 123.53, 121.49, 117.92, 106.61, 102.60, 29.62 (NCH_2_), 29.27 (NCH_2_), 24.61 (NC*C*H_2_CC), 22.61 (NC*C*H_2_CC), 14.04 (CC*C*H_2_ CC); IR (neat) 3207 (m, NH), 2854 (s), 1606 (s, C=O), 1445 (s), 1278 (m), 1113 (w), 1022 (w), 796 (m) cm^−1^; HRMS (ESI-TOF) *m/z* [M]^+^ calcd for C_19_H_19_N_3_O_2_ 321.1477, found 321.1480.

#### 4.3.3. Azepan-1-yl(5-[3-(1*H*-pyrazol-3-yl)phenyl]furan-2-yl)methanone (**10c**)

The standard procedure 1 was followed by use of furoic acid **8** (60.1 mg, 0.236 mmol, 1.0 equiv), azepane (**9c**, 28.0 mg, 0.282 mmol, 1.2 equiv), EDCI (40.3 mg, 0.259 mmol, 1.1 equiv), HOBt (34.9 mg, 0.259 mmol, 1.1 equiv), and DIPEA (61.0 mg, 0.472 mmol, 2.0 equiv). After the solution was stirred at 25 °C for 12 h and then worked up, the residue was purified by use of column chromatography (45% EtOAc in hexanes as the eluent) to give the pure polycycle **10c** (67.3 mg, 0.201 mmol) in 85% yield as colorless oil: TLC R*_f_* 0.45 (90% EtOAc as the eluent); ^1^H-NMR (CDCl_3_) *δ* 8.09 (s, 1 H, ArH), 7.68 (d, *J* = 7.6 Hz, 2 H, 2 × ArH), 7.61–7.59 (m, 2 H, ArH + pyrazole), 7.42 (d, *J* = 7.6 Hz, 1 H, ArH), 7.08 (d, *J* = 3.6 Hz, 1 H, furan), 6.74 (d, *J* = 3.6 Hz, 1 H, furan), 6.62 (s, 1 H, pyrazole), 3.82–3.66 (m, 4 H, 2 × NCH_2_), 1.92–1.81 (m, 4 H, 2 × NCCH_2_C), 1.26–1.23 (m, 4 H, 2 × CCCH_2_CC); ^13^C-NMR (CDCl_3_) *δ* 159.90 (C=O), 154.57, 148.37 (C=N), 147.26, 132.76, 132.66, 130.10, 129.02, 125.49, 123.33, 121.37, 118.32, 106.66, 102.44, 48.64 (NCH_2_), 47.30 (NCH_2_), 30.04 (NC*C*H_2_C), 27.29 (NC*C*H_2_C), 27.22 (CC*C*H_2_CC), 27.11 (CC*C*H_2_CC); IR (neat) 3225 (m, NH), 2917 (m), 1610 (s, C=O), 1459 (m), 1283 (m), 1113 (s), 781 (s), 738 (w) cm^−1^; HRMS (ESI-TOF) *m/z* [M]^+^ calcd for C_20_H_21_N_3_O_2_ 335.1633, found 335.1632.

#### 4.3.4. *N*-(4-Fluorophenyl)-5-[3-(1*H*-pyrazol-3-yl)phenyl]furan-2-carboxamide (**12a**)

The standard procedure 1 was followed by use of furoic acid **8** (62.9 mg, 0.247mmol, 1.0 equiv), 4-fluoroaniline (**11a**, 33.0 mg, 0.296 mmol, 1.2 equiv), EDCI (42.1 mg, 0.271 mmol, 1.1 equiv), HOBt (36.6 mg, 0.271 mmol, 1.1 equiv), and DIPEA (63.8 mg, 0.494 mmol, 2.0 equiv). After the solution was stirred at 25 °C for 10 h and then worked up, the residue was purified by use of column chromatography (40% EtOAc in hexanes as the eluent) to give the pure polycycle **12a** (76.4 mg, 0.198 mmol) in 88% yield as colorless oil: TLC R*_f_* 0.49 (80% EtOAc as the eluent); ^1^H-NMR (DMSO-*d_6_*) *δ* 7.89 (s, 1 H, ArH), 7.88–7.83 (m, 2 H, 2 × ArH), 7.80–7.77 (m, 4 H, 4 × ArH), 7.52 (t, *J* = 6.8 Hz, 1 H, ArH), 7.42 (d, *J* = 3.6 Hz, 1 H, furan), 7.24–7.19 (m, 2 H, furan + pyrazole), 6.85 (s, 1 H, pyrazole); ^13^C-NMR (DMSO-*d_6_*) *δ* 158.51 d, *J_CF_* = 239 Hz), 156.11 (C=O), 155.27, 146.66 (C=N), 134.78, 134.76, 130.06, 129.73, 129.38, 125.51, 123.55, 122.95, 122.65 (d, *J_CF_* = 7.6 Hz), 121.14, 117.13, 115.36 (d, *J_CF_* = 22 Hz), 108.22, 102.28; IR (neat) 3268 (m, NH), 2922 (w), 1648 (s, C=O), 1505 (m), 1211 (m), 1093 (w), 817 (w), 784 (w) cm^−1^; HRMS (ESI-TOF) *m/z* [M]^+^ calcd for C_20_H_14_FN_3_O_2_ 347.1070, found 347.1069.

#### 4.3.5. *N*-(4-Methoxyphenyl)-5-[3-(1*H*-pyrazol-3-yl)phenyl]-furan-2-carboxamide (**12b**)

The standard procedure 1 was followed by use of furoic acid **8** (58.4 mg, 0.229 mmol, 1.0 equiv), 4-methoxyaniline (**11b**, 33.9 mg, 0.275 mmol, 1.2 equiv), EDCI (39.1 mg, 0.251 mmol, 1.1 equiv), HOBt (33.9 mg, 0.251 mmol, 1.1 equiv), and DIPEA (59.2 mg, 0.458 mmol, 2.0 equiv). After the solution was stirred at 25 °C for 12 h and then worked up, the residue was purified by use of column chromatography (30% EtOAc in hexanes as the eluent) to give the desired polycycle **12b** (73.4 mg, 0.202 mmol) in 89% yield as colorless oil: TLC R*_f_* 0.46 (60% EtOAc as the eluent); ^1^H-NMR (DMSO-*d_6_*) 8.30 (s, 1 H, ArH), 7.86 (d, *J* = 7.6 Hz, 1 H, ArH), 7.79 (d, *J* = 7.6 Hz, 1 H, ArH), 7.73 (brs, 1 H, pyrazole), 7.61 (d, *J* = 8.8 Hz, 2 H, 2 × ArH), 7.50 (t, *J* = 8.0 Hz, 1 H, ArH), 7.35 (d, *J* = 3.6 Hz, 1 H, furan), 7.19 (d, *J* = 3.6 Hz, 1 H, furan), 6.92 (d, *J* = 8.8 Hz, 2 H, 2 × ArH), 6.82 (d, *J* = 2.4 Hz, 1 H, pyrazole), 3.72 (s, 3 H, OCH_3_); ^13^C-NMR (DMSO-*d_6_*) *δ* 156.81 (C=O), 156.43, 155.42, 146.44 (C=N), 132.78, 132.20, 130.15, 129.85, 129.10, 125.79, 123.85, 122.59, 122.48, 121.27, 117.03, 107.57, 102.44, 102.41, 55.14 (OCH_3_); IR (neat) 3205 (m, NH), 2961 (w), 1637 (m, C=O), 1509 (s), 1246 (m), 1025 (w), 832 (w), 744 (w) cm^−1^; HRMS (ESI-TOF) *m/z* [M + H]^+^ calcd for C_21_H_17_N_3_O_3_ + H 360.1349, found 360.1342.

#### 4.3.6. *N*-Cyclopropyl-5-[3-(1*H*-pyrazol-3-yl)phenyl]furan-2-carboxamide (**13**)

The standard procedure 1 was followed by use of furoic acid **8** (68.3 mg, 0.268 mmol, 1.0 equiv), cyclopropylamine (18.3 mg, 0.322 mmol, 1.2 equiv), EDCI (45.7 mg, 0.294 mmol, 1.1 equiv), HOBt (39.7 mg, 0.294 mmol, 1.1 equiv), and DIPEA (69.2 mg, 0.536 mmol, 2.0 equiv). After the solution was stirred at 25 °C for 12 h and then worked up, the residue was purified by use of column chromatography (40% EtOAc in hexanes as the eluent) to give the pure polycycle **13** (63.2 mg, 0.215 mmol) in 87% yield as colorless oil: TLC R*_f_* 0.55 (80% EtOAc as the eluent); ^1^H-NMR (CDCl_3_) *δ* 8.14 (s, 1 H, ArH), 7.65 (d, *J* = 7.6 Hz, 1 H, ArH), 7.62 (d, *J* = 2.0 Hz, 1 H, pyrazole), 7.57–7.55 (m, 1 H, ArH), 7.39 (t, *J* = 7.6 Hz, 1 H, ArH), 7.15 (d, *J* = 3.6 Hz, 1 H, furan), 6.71 (d, *J* = 3.6 Hz, 1 H, furan), 6.63 (d, *J* = 2.0 Hz, 1 H, pyrazole), 2.92–2.72 (m, 1 H, NCH), 0.84–0.79 (m, 2 H, CH_2_), 0.66–0.63 (m, 2 H, CH_2_); ^13^C-NMR (CDCl_3_) *δ* 159.68 (C=O), 155.07, 148.88 (C=N), 147.14, 133.23, 131.79, 130.18, 129.23, 126.04, 124.02, 121.52, 116.35, 107.74, 102.93, 22.36 (NCH), 6.77 (CH_2_) ; IR (neat) 3226 (s, NH), 2126 (w), 1634 (s, C=O), 1547 (m), 1306 (m), 1023 (w), 795 (m), 691 (w) cm^−1^; HRMS (ESI-TOF) *m/z* [M]^+^ calcd for C_17_H_15_N_3_O_2_ 293.1164, found 293.1160.

#### 4.3.7. *N*-(2-Methoxyethyl)-5-[3-(1*H*-pyrazol-3-yl)phenyl]thiophene-2-carboxamide (**14**)

The standard procedure 1 was followed by use of 5-[3-(1*H*-pyrazol-3-yl)phenyl]thiophene-2-carboxylic acid (85.2 mg, 0.314 mmol, 1.0 equiv), 2-methoxyethylamine (28.3 mg, 0.377 mmol, 1.2 equiv), EDCI (53.6 mg, 0.345 mmol, 1.1 equiv), HOBt (46.6 mg, 0.345 mmol, 1.1 equiv), and DIPEA (81.1 mg, 0.628 mmol, 2.0 equiv). After the solution was stirred at 25 °C for 10 h and then worked up, the residue was purified by use of column chromatography (45% EtOAc in hexanes as the eluent) to give the desired polycycle **14** (91.6 mg, 0.280 mmol) in 89% yield as colorless oil: TLC R*_f_* 0.53 (90% EtOAc as the eluent); ^1^H-NMR (CDCl_3_) *δ* 7.95 (s, 1 H, ArH), 7.65–7.59 (m, 2 H, 2 × ArH), 7.47–7.45 (m, 1 H, pyrazole), 7.33 (t, *J* = 7.6 Hz, 1 H, ArH), 7.31–7.17 (m, 1 H, thiophene), 6.88 (s, 1 H, thiophene), 6.60 (d, *J* = 2.0 Hz, 1 H, pyrazole), 3.61–3.60 (m, 2 H, OCH_2_), 3.55–3.36 (m, 2 H, NCH_2_), 3.36 (s, 3 H, OCH_3_); ^13^C-NMR (CDCl_3_) *δ* 162.10 (C=O), 151.99, 148.58 (C=N), 148.41, 137.41, 133.88, 131.31, 129.36, 129.16, 125.82, 125.75, 123.63, 103.91, 102.77, 71.20 (OCH_2_), 58.73 (OCH_3_), 39.63 (NCH_2_); IR (neat) 3301 (s, NH), 2928 (m), 1633 (s, C=O), 1552 (s), 1308 (m), 1197 (w), 1093 (m), 766 (m) cm^−1^; HRMS (ESI-TOF) *m/z* [M]^+^ calcd for C_17_H_17_N_3_O_2_S 327.1041, found 327.1038.

### 4.4. Standard Procedure 2 for the Synthesis of Benzaldehyde Derivatives ***18a*,*18b*** and ***18c***

To a solution containing amides 16 (1.0 equiv) in DMAc (25–30 mL) was added 3-bromobenzaldehyde (17, 1.2 equiv), Pd(OAc)_2_ (0.20 equiv), and KOAc (2.0 equiv). The reaction mixture was stirred at 130 °C for 20–24 h. After being cooled to 25 °C, the reaction mixture was quenched with water (75 mL). The solution was extracted with EtOAc (3 × 50 mL) and the combined organic layers were then dried over MgSO_4_ (s), filtered, and concentrated under reduced pressure to afford a residue. The residue was purified by use of column chromatography packed with silica gel to give the desired hinged products.

#### 4.4.1. 3-[5-(Morpholine-4-carbonyl)furan-2-yl]benzaldehyde (**18a**)

The standard procedure 2 was followed by use of amide **16a** (1.56 g, 8.58 mmol, 1.0 equiv), 3-bromobenzaldehyde (**17**, 1.19 g, 10.3 mmol, 1.2 equiv), Pd(OAc)_2_ (0.386 g, 1.76 mmol, 0.20 equiv), and KOAc (1.68 g, 17.2 mmol, 2.0 equiv). After the solution was stirred at 130 °C for 20 h and then worked up, the residue was purified by use of column chromatography (45% EtOAc in hexanes as the eluent) to give the desired aldehyde **18a** (2.01 g, 7.04 mmol) in 82% yield as colorless oil: TLC R*_f_* 0.40 (40% EtOAc as the eluent); ^1^H-NMR (CDCl_3_) *δ* 10.04 (s, 1 H, ArCHO), 8.14 (s, 1 H, ArH), 7.91 (d, *J* = 7.6 Hz, 1 H, ArH), 7.81 (d, *J* = 7.6 Hz, 1 H, ArH), 7.60 (t, *J* = 8.0 Hz, 1 H, ArH), 7.09 (d, *J* = 3.6 Hz, 1 H, furan), 6.82 (d, *J* = 3.6 Hz, 1 H, furan), 3.78–3.76 (m, 8 H, 4 × CH_2_); ^13^C-NMR (CDCl_3_) *δ* 191.42 (CHO), 158.45 (C=O), 153.27, 146.74, 136.41, 130.17, 129.32, 129.28, 129.17, 124.61, 118.34, 107.35, 66.48 (OCH_2_), 45.89 (NCH_2_); IR (neat) 2855 (m),1700 (m), 1617 (s, C=O), 1425 (s), 1275 (m), 1113 (m), 795 (m), 594 (w) cm^−1^; HRMS (ESI-TOF) *m/z* [M + H]^+^ calcd for C_16_H_15_NO_4_ + H 286.1091, found 286.1086.

#### 4.4.2. 3-[5-(Morpholine-4-carbonyl)thiophen-2-yl]benzaldehyde (**18b**)

The standard procedure 2 was followed by use of amide **16b** (1.85 g, 9.38 mmol, 1.0 equiv), 3-bromobenzaldehyde (**17**, 2.09 g, 11.2 mmol, 1.2 equiv), Pd(OAc)_2_ (0.417 g, 0.187 mmol, 0.20 equiv), and KOAc (3.94 g, 18.7 mmol, 2.0 equiv). After the solution was stirred at 130 °C for 22 h and then worked up, the residue was purified by use of column chromatography (35% EtOAc in hexanes as the eluent) to give the pure aldehyde **18b** (2.25 g, 7.49 mmol) in 80% yield as colorless oil: TLC R*_f_* 0.45 (40% EtOAc as the eluent); ^1^H-NMR (CDCl_3_) *δ* 10.03 (s, 1 H, ArCHO), 8.06 (s, 1 H, ArH), 7.82–7.78 (m, 2 H, ArH + thiophene), 7.55 (d, *J* = 7.6 Hz, 1 H, ArH), 7.29–7.26 (m, 2 H, ArH + thiophene), 3.77–3.71 (m, 8 H, 4 × CH_2_); ^13^C-NMR (CDCl_3_) *δ* 191.43 (CHO), 162.82 (C=O), 145.79, 136.79, 136.30, 134.19, 131.53, 130.00, 129.65, 129.43, 126.42, 123.43, 66.80 (OCH_2_), 45.90 (NCH_2_); IR (neat) 2918 (m), 1700 (s, C=O), 1617 (s, C=O), 1535 (m), 1163 (m), 1113 (s), 1066 (m), 734 (m), 683(w) cm^−1^; HRMS (ESI-TOF) *m/z* [M + H]^+^ calcd for C_16_H_15_NO_3_S + H 302.0852, found 302.0861.

#### 4.4.3. 3-[1-Methyl-5-(morpholine-4-carbonyl)-1*H*-pyrrol-2-yl]benzaldehyde (**18c**)

The standard procedure 2 was followed by use of amide **16c** (1.62 g, 8.34 mmol, 1.0 equiv), 3-bromobenzaldehyde (**17**, 1.86 g, 10.0 mmol, 1.2 equiv), Pd(OAc)_2_ (0.374 g, 0.166 mmol, 0.20 equiv), and KOAc (3.54 g, 16.6 mmol, 2.0 equiv). After the solution was stirred at 130 °C for 24 h and then worked up, the residue was purified by use of column chromatography (35% EtOAc in hexanes as the eluent) to give the desired aldehyde **18c** (2.11 g, 7.09 mmol) in 85% yield as colorless oil: TLC R*_f_* 0.30 (50% EtOAc in hexanes as the eluent); ^1^H-NMR (CDCl_3_) *δ* 9.98 (s, 1 H, ArCHO), 7.85 (s, 1 H, ArH), 7.80 (d, *J* = 7.2 Hz, 1 H, ArH), 7.62 (d, *J* = 7.2 Hz, 1 H, ArH), 7.55 (t, *J* = 7.6 Hz, 1 H, ArH), 6.34 (d, *J* = 3.6 Hz, 1 H, pyrrole), 6.17 (d, *J* = 3.6 Hz, 1 H, pyrrole), 3.75 (s, 3 H, CH_3_), 3.74–3.68 (m, 8 H, 4 × CH_2_); ^13^C-NMR (CDCl_3_) *δ* 191.77 (CHO), 162.80 (C=O), 136.95, 136.41, 134.60, 133.20, 129.85, 129.04, 128.57, 126.66, 112.59, 108.40, 66.81 (OCH_2_), 45.41 (NCH_2_), 33.70 (NCH_3_); IR (neat) 2921 (w), 2854 (w), 1697 (s, C=O), 1620 (m), 1432 (m), 1279 (m), 1114 (m), 1028 (w), 794 (w); HRMS (ESI-TOF) *m/z* [M + H]^+^ calcd for C_17_H_18_N_2_O_3_ + H 299.1397, found 299.1391.

### 4.5. Standard Procedure 3 for the Synthesis of Polycyclic Derivatives ***21***

The aldehyde 18 (1.0 equiv) was added to a solution of TsNHNH_2_ (1.2 equiv) in CH_3_CN. After the mixture was stirred at 25 °C for 3.0 h, a solution of aqueous NaOH (5.0 N, 5.0 equiv) was added and the mixture was stirred for another 20 min. The alkyne 22 (3.0 equiv) was then added and the mixture was stirred at 50 °C for 44–48 h. The volatiles in the reaction mixture were evaporated under reduced pressure and the residue was dissolved in a mixture of water-ethyl acetate (1:1, 70–80 mL). The solution was extracted with EtOAc (3 × 15 mL) and the combined organic layers were then dried over MgSO_4_ (s), filtered, and concentrated under reduced pressure to afford a residue. The residue was purified by use of column chromatography packed with silica gel to give the desired polycycle.

#### 4.5.1. Morpholino-(5-[3-(1*H*-pyrazol-3-yl)phenyl]-1-methyl-1*H*-furan-2-yl)methanone (**21a**)

The benzaldehyde **18a** (125 mg, 0.438 mmol, 1.0 equiv) was added to a solution of TsNHNH_2_ (186 mg, 0.526 mmol, 1.2 equiv) in CH_3_CN. After the mixture was stirred at 25 °C for 3.0 h, a solution of aqueous NaOH (5.0 N, 995 µL, 2.19 mmol, 5.0 equiv) was added and the mixture was stirred for further 20 min. Trimethylsilyl acetylene (**5**, 128 mg, 1.31 mmol, 3.0 equiv) was then added and the mixture was stirred at 50 °C for another 45 h. The volatiles in the reaction mixture were evaporated under reduced pressure and the residue was dissolved in a mixture of water-ethyl acetate (1:1, 40–50 mL). The solution was extracted with EtOAc (3 × 15 mL) and the combined organic layers were then dried over MgSO_4_ (s), filtered, and concentrated under reduced pressure to afford a residue. The residue (0.173 g, 0.437 mmol, 1.0 equiv) was dissolved in dry THF and added TBAF (114 mg, 0.437 mmol, 1.0 equiv). After the mixture was stirred at 25 °C for 3.0 h, it was quenched with water. The solution was extracted with EtOAc (3 × 15 mL) and the combined organic layers were then dried over MgSO_4_ (s), filtered, and concentrated under reduced pressure to afford a residue. The residue was purified by use of column chromatography to give the pure polycycle **21a** (129 mg, 0.404 mmol) in 92% yield as pale yellow oil: TLC R*_f_* 0.30 (50% EtOAc as the eluent); ^1^H-NMR (CDCl_3_) *δ* 8.08 (s, 1 H, ArH), 7.71 (d, *J* = 7.6 Hz, 1 H, ArH), 7.64–7.60 (m, 2 H, ArH + pyrazole), 7.46–7.44 (m, 1 H, ArH), 7.12 (d, *J* = 4.0 Hz, 1 H, furan), 6.78 (d, *J* = 4.0 Hz, 1 H, furan), 6.66 (s, 1 H, pyrazole), 3.88–3.76 (m, 8 H, 4 × CH_2_); ^13^C-NMR (CDCl_3_) *δ* 159.83 (C=O), 155.03, 149.89 (C=N), 146.88, 133.20, 131.99, 130.18, 129.33, 125.92, 123.75, 121.57, 119.01, 106.88, 102.86, 66.99 (OCH_2_), 45.69 (NCH_2_); IR (neat) 3215 (m, NH), 2964 (w), 1618 (s, C=O), 1437 (s), 1280 (s), 1114 (s), 1028 (s), 766 (s), 592 (w) cm^−1^; HRMS (ESI-TOF) *m/z* calcd for C_18_H_17_N_3_O_3_ 323.1271, found 323.1270.

#### 4.5.2. Morpholino-(5-[3-(5-methyl-1*H*-pyrazol-3-yl)phenyl]furan-2-yl)methanone (**21b**)

The standard procedure 3 was followed by use of benzaldehyde **18a** (85.5 mg, 0.289 mmol, 1.0 equiv), TsNHNH_2_(64.6 mg, 0.347 mmol, 1.2 equiv), propyne (**22b**, 34.7 mg, 0.867 mmol, 3.0 equiv), and NaOH (344 µL, 1.44 mmol, 5.0 equiv). After the solution was stirred at 50 °C for 44 h and then worked up, the residue was purified by use of column chromatography (30% EtOAc in hexanes as the eluent) to give the pure polycycle **21b** (82.2 mg, 0.254 mmol) in 87% yield as colorless oil: TLC R*_f_* 0.35 (50% EtOAc as the eluent); ^1^H-NMR (CDCl_3_) *δ* 8.03 (s, 1 H, ArH), 7.66 (d, *J* = 7.4 Hz, 1 H, ArH), 7.58 (d, *J* = 7.4 Hz, 1 H, ArH), 7.43–7.41 (m, 1 H, ArH), 7.10 (d, *J* = 3.6 Hz, 1 H, furan), 6.75 (d, *J* = 3.6 Hz, 1 H, furan), 6.37 (s, 1 H, pyrazole), 3.87–3.75 (m, 8 H, 4 × CH_2_), 2.35 (s, 3 H, CH_3_); ^13^C-NMR (CDCl_3_) *δ* 159.06 (C=O), 155.08, 146.65 (C=N), 142.68, 133.68, 133.48, 130.05, 129.23, 125.78, 123.55, 121.41, 119.06, 106.81, 102.13, 66.98 (OCH_2_), 45.65 (NCH_2_), 11.50 (CH_3_); IR (neat) 3500 (w, NH), 2855 (w), 1697 (s), 1617 (s, C=O), 1425 (m), 1275 (s), 1114 (m), 794 (w) cm^−1^; HRMS (ESI-TOF) *m/z* [M]^+^ calcd for C_19_H_19_N_3_O_3_ 337.1426, found 337.1427.

#### 4.5.3. Morpholino-(5-[3-(5-phenyl-1*H*-pyrazol-3-yl)phenyl]furan-2-yl)methanone (**21c**)

The standard procedure 3 was followed by use of benzaldehyde **18a** (65.2 mg, 0.228 mmol, 1.0 equiv), TsNHNH_2_ (51.1 mg, 0.274 mmol, 1.2 equiv), phenylacetylene (**22c**, 69.9 mg, 0.684 mmol, 3.0 equiv), and NaOH (241 µL, 1.14 mmol, 5.0 equiv). After the solution was stirred at 50 °C for 45 h and then worked up, the residue was purified by use of column chromatography (35% EtOAc in hexanes as the eluent) to give the pure polycycle **21c** (83.9 mg, 0.210 mmol) in 92% yield as colorless oil: TLC R*_f_* 0.50 (70% EtOAc as the eluent); ^1^H-NMR (CDCl_3_) *δ* 8.05 (s, 1 H, ArH), 7.69–7.68 (m, 3 H, 3 × ArH), 7.67–7.66 (m, 1 H, ArH), 7.58–7.56 (m, 3 H, 3 × ArH), 7.42–7.31 (m, 1 H, ArH), 7.06 (d, *J* = 3.6 Hz, 1 H, furan), 6.85 (s, 1 H, pyrazole), 6.70 (d, *J* = 3.6 Hz, 1 H, furan), 3.87–3.76 (m, 4 H, 2 × OCH_2_), 3.75–3.73 (m, 4 H, 2 × NCH_2_); ^13^C-NMR (CDCl_3_) *δ* 159.10 (C=O), 154.93, 148.53 (C=N), 146.58, 132.65, 132.45, 130.57, 130.18, 129.35, 128.94, 128.43, 125.79, 125.55, 123.84, 121.47, 118.94, 106.90, 100.23, 66.95 (OCH_2_), 46.58 (NCH_2_); IR (neat) 3225 (w, NH), 2921 (s), 2340 (w), 1680 (s, C=O), 1456 (m), 1179 (w), 1276 (m), 1114 (m), 764 (w) cm^−1^; HRMS (ESI-TOF) *m/z* [M]^+^ calcd for C_24_H_21_N_3_O_3_ 399.1582, found 399.1583.

#### 4.5.4. Morpholino[5-(3-[5-(*p*-tolyl)-1*H*-pyrazol-3-yl]phenyl)furan-2-yl]methanone (**21d**)

The standard procedure 3 was followed by use of benzaldehyde **18a** (88.1 mg, 0.309 mmol, 1.0 equiv), TsNHNH_2_ (69.0 mg, 0.370 mmol, 1.2 equiv), *p*-tolylacetylene (**22d**, 107 mg, 0.927 mmol, 3.0 equiv), and NaOH (418 µL, 1.54 mmol, 5.0 equiv). After the solution was stirred at 50 °C for 48 h and then worked up, the residue was purified by use of column chromatography (35% EtOAc in hexanes as the eluent) to give the pure polycycle **21d** (113 mg, 0.273 mmol) in 88% yield as colorless oil: TLC R*_f_* 0.40 (50% EtOAc as the eluent); ^1^H-NMR (CDCl_3_) *δ* 7.94 (s, 1 H, ArH), 7.57 (d, *J* =7.4 Hz, 1 H, ArH), 7.52 (d, *J* = 6.8 Hz, 2 H, 2 × ArH), 7.43 (d, *J* = 6.8 Hz, 2 H, 2 × ArH), 7.23 (s, 1 H, furan), 7.00 (d, *J* = 7.4 Hz, 2 H, 2 × ArH), 6.71 (s, 1 H, furan), 6.48 (s, 1 H, pyrazole), 3.78–3.68 (m, 8 H, 4 × CH_2_), 2.23 (s, 3 H, ArCH_3_); ^13^C-NMR (CDCl_3_) *δ* 158.89 (C=O), 154.85, 148.73 (C=N), 147.53, 146.23, 137.76, 132.23, 129.70, 129.26, 128.97, 127.57, 125.57, 125.27, 123.26, 121.25, 118.89, 106.19, 99.50, 66.69 (OCH_2_), 45.95 (NCH_2_), 20.98 (CH_3_); IR (neat) 3216 (m, NH), 2296 (w), 1610 (s, C=O), 1523 (m), 1437 (m), 1280 (m), 1115 (m), 1029 (s), 745 (m) cm^−1^; HRMS (ESI-TOF) *m/z* [M]^+^ calcd for C_25_H_23_N_3_O_3_ 413.1739, found 413.1738.

#### 4.5.5. Morpholino[5-(3-[5-(4-methoxyphenyl)-1*H*-pyrazol-3-yl]phenyl)furan-2-yl]methanone (**21e**)

The standard procedure 3 was followed by use of benzaldehyde **18a** (95.0 mg, 0.333 mmol, 1.0 equiv), TsNHNH_2_ (74.4 mg, 0.399 mmol, 1.2 equiv), *p*-methoxyphenylacetylene (**22e**, 132 mg, 0.999 mmol, 3.0 equiv), and NaOH (354 µL, 1.66 mmol, 5.0 equiv). After the solution was stirred at 50 °C for 46 h and then worked up, the residue was purified by use of column chromatography (45% EtOAc in hexanes as the eluent) to give the pure polycycle **21e** (129 mg, 0.299 mmol) in 90% yield as colorless oil: TLC R*_f_* 0.55 (90% EtOAc as the eluent); ^1^H-NMR (CDCl_3_) *δ* 7.95 (s, 1 H, ArH), 7.58–7.52 (m, 3 H, 3 × ArH), 7.42 (d, *J* = 7.6 Hz, 1 H, ArH), 7.24 (t, *J* = 7.6 Hz, 1 H, ArH), 6.96 (d, *J* = 3.6 Hz, 1 H, furan), 6.74–6.66 (m, 3 H, 2 × ArH + pyrazole), 6.52 (d, *J* = 3.6 Hz, 1 H, furan), 3.77–3.66 (m, 8 H, 4 × CH_2_); ^13^C-NMR (CDCl_3_) *δ* 159.34 (C=O), 158.87, 154.83, 148.58 (C=N), 147.33, 146.11, 133.28, 129.74, 128.96, 126.66, 125.55, 123.28, 123.10, 121.20, 118.85, 113.95, 106.62, 99.14, 66.62 (OCH_2_), 54.92 (OCH_3_), 45.92 (NCH_2_); IR (neat) 3219 (w, NH), 1607 (m, C=O), 1434 (w), 1325 (m), 1256 (m), 1114 (m), 1061 (m), 750 (s) cm^−1^; HRMS (ESI-TOF) *m/z* [M]^+^ calcd for C_25_H_23_N_3_O_4_ 429.1688, found 429.1687.

#### 4.5.6. Morpholino(5-[3-(5-[4-(trifluoromethyl)phenyl]-1*H*-pyrazol-3-yl)phenyl]furan-2-yl)-methanone (**21f**)

The standard procedure 3 was followed by use of benzaldehyde **18a** (73.2 mg, 0.256 mmol, 1.0 equiv), TsNHNH_2_ (57.3 mg, 0.308 mmol, 1.2 equiv), *p*-(trifluoromethyl)phenylacetylene (**22f**, 130 mg, 0.768 mmol, 3.0 equiv), and NaOH (275 µL, 1.28 mmol, 5.0 equiv). After the solution was stirred at 50 °C for 46 h and then worked up, the residue was purified by use of column chromatography (45% EtOAc in hexanes as the eluent) to give the pure polycycle **21f** (105 mg, 0.225 mmol) in 88% yield as colorless oil: TLC R*_f_* 0.53 (90% EtOAc as the eluent); ^1^H-NMR (CDCl_3_) *δ* 7.89 (s, 1 H, ArH), 7.68 (d, *J* = 8.0 Hz, 2 H, 2 × ArH), 7.54 (d, *J* = 8.0 Hz, 2 H, 2 × ArH), 7.41 (d, *J* = 7.6 Hz, 2 H, 2 × ArH), 7.24 (t, *J* = 7.6 Hz, 1 H, ArH), 6.94 (d, *J* = 3.4 Hz, 1 H, furan), 6.80 (s, 1 H, pyrazole), 6.49 (d, *J* = 3.4 Hz, 1 H, furan), 3.80–3.70 (m, 8 H, 4 × CH_2_); ^13^C-NMR (CDCl_3_) *δ* 159.13 (C=O), 154.72, 148.00, 147.53, 146.24, 134.60, 131.09, 130.04, 129.82, 129.51 (q, *J_CF_* = 32.4 Hz), 129.27, 125.51, 125.29, 123.85 (q, *J_CF_* = 270.5 Hz), 122.59, 121.30, 118.59, 106.74, 100.62, 66.79 (OCH_2_), 45.24 (NCH_2_); IR (neat) 3504 (w, NH), 2856 (w), 1698 (s), 1618 (s, C=O), 1431 (m), 1279 (m), 1115 (s), 1029 (m) cm^−1^; HRMS (ESI-TOF) *m/z* [M]^+^ calcd for C_25_H_20_F_3_N_3_O_3_ 467.1457, found 467.1455.

#### 4.5.7. Morpholino(5-[3-(5-phenyl-1*H*-pyrazol-3-yl)phenyl]thiophen-2-yl)methanone (**21g**)

The standard procedure 3 was followed by use of benzaldehyde **18b** (78.6 mg, 0.261 mmol, 1.0 equiv), TsNHNH_2_ (58.3 mg, 0.313 mmol, 1.2 equiv), phenylacetylene (**22c**, 80.1 mg, 0.783 mmol, 3.0 equiv), and NaOH (313 µL, 1.30 mmol, 5.0 equiv). After the solution was stirred at 50 °C for 46 h and then worked up, the residue was purified by use of column chromatography (40% EtOAc in hexanes as the eluent) to give the pure polycycle **21g** (99.7 mg, 0.240 mmol) in 92% yield as white solids: mp (recrystallized from EtOH) 239.6–241.8 °C; TLC R*_f_* 0.52 (80% EtOAc as the eluent); ^1^H-NMR (CDCl_3_) *δ* 7.81 (s, 1 H, ArH), 7.51–7.47 (m, 3 H, 3 × ArH), 7.24–7.22 (m, 1 H, ArH), 7.16–7.10 (m, 4 H, 3 × ArH + thiophene), 7.05–6.96 (m, 2 H, ArH + thiophene), 6.60 (s, 1 H, pyrazole), 3.48–3.44 (m, 8 H, 4 × CH_2_); ^13^C-NMR (CDCl_3_) *δ* 162.56 (C=O), 147.22 (C=N), 142.51, 135.01, 133.09, 132.56, 132.35, 129.49, 128.81, 128.31, 128.10, 127.24, 126.19, 124.90, 124.85, 124.58, 122.07, 98.95, 66.08 (OCH_2_), 45.50 (NCH_2_); IR (KBr pellet) 3167 (w, NH), 2921 (w), 1606 (m, C=O), 1583 (m), 1433 (s), 1080 (w), 1018 (w), 792 (w), 741 (w) cm^−1^; HRMS (ESI-TOF) *m/z* [M]^+^ calcd for C_24_H_21_N_3_O_2_S 415.1354, found 415.1353.

#### 4.5.8. [5-(3-[5-(4-Methoxyphenyl)-1*H*-pyrazol-3-yl]phenyl)thiophen-2-yl](morpholino)methanone (**21h**)

The standard procedure 3 was followed by use of benzaldehyde **18b** (95.2 mg, 0.316 mmol, 1.0 equiv), TsNHNH_2_ (70.6 mg, 0.379 mmol, 1.2 equiv), *p*-methoxyphenylacetylene (**22e**, 0.408 mL, 0.948 mmol, 3.0 equiv), and NaOH (316 µL, 1.58 mmol, 5.0 equiv). After the solution was stirred at 50 °C for 48 h and then worked up, the residue was purified by use of column chromatography (45% EtOAc in hexanes as the eluent) to give the pure polycycle **21h** (129 mg, 0.290 mmol) in 92% yield as colorless oil: TLC R*_f_* 0.48 (90% EtOAc as the eluent); ^1^H-NMR (CDCl_3_) *δ* 7.95 (s, 1 H, ArH), 7.63 (d, *J* = 7.6 Hz, 1 H, ArH), 7.57 (d, *J* = 8.0 Hz, 2 H, 2 × ArH), 7.48 (d, *J* = 7.2 Hz, 1 H, ArH), 7.34 (t, *J* = 7.6 Hz, 1 H, ArH), 7.23–7.20 (m, 2 H, 2 × ArH), 7.17–7.16 (m, 1 H, thiophene), 6.85 (d, *J* = 8.0 Hz, 1 H, thiophene), 6.74 (s, 1 H, pyrazole), 3.77–3.74 (m, 4 H, 2 × OCH_2_), 3.71–3.70 (m, 4 H, 2 × NCH_2_); ^13^C-NMR (CDCl_3_) *δ* 163.42 (C=O), 160.31, 149.31 (C=N), 147.57, 147.36, 147.31, 135.43, 133.86, 132.72, 130.31, 129.46, 126.88, 125.62, 123.15, 122.97, 122.96, 114.31, 99.61, 66.84 (OCH_2_), 55.27 (OCH_3_), 45.57 (NCH_2_); IR (neat) 3225 (w, NH), 2285 (w), 1601 (s, C=O), 1325 (s), 1262 (w), 1114 (s), 1067 (m), 749 (w) cm^−1^; HRMS (ESI-TOF) *m/z* [M]^+^ calcd for C_25_H_23_N_3_O_3_S 445.1460, found 445.1458.

#### 4.5.9. Morpholino(5-[3-(5-[4-(trifluoromethyl)phenyl]-1*H*-pyrazol-3-yl)phenyl]thiophen-2-yl)-methanone (**21i**)

The standard procedure 3 was followed by use of benzaldehyde **18b** (73.5 mg, 0.249 mmol, 1.0 equiv) in CH_3_CN (3.5 mL) was added TsNHNH_2_ (54.5 mg, 0.292 mmol, 1.2 equiv), 4*-*(trifluoromethyl)phenyl acetylene (**22f**, 124 mg, 0.732 mmol, 3.0 equiv), and NaOH (290 µL, 1.24 mmol, 5.0 equiv). After the solution was stirred at 50 °C for 46 h and then worked up, the residue was purified by use of column chromatography (45% EtOAc in hexanes as the eluent) to give the pure polycycle **21i** (108 mg, 0.224 mmol) in 90% yield as colorless oil: TLC R*_f_* 0.56 (90% EtOAc as the eluent); ^1^H-NMR (CDCl_3_) *δ* 7.81 (s, 1 H, ArH), 7.51–7.47 (m, 2 H, 2 × ArH), 7.25–7.22 (m, 2 H, 2 × ArH), 7.16–7.10 (m, 4 H, 3 × ArH + thiophene), 7.05–6.96 (m, 1 H, thiophene), 6.60 (s, 1 H, pyrazole), 3.48–3.44 (m, 8 H, 4 × CH_2_); ^13^C-NMR (DMSO-*d_6_*) *δ* 163.04 (C=O), 160.61, 158.25, 154.09, 146.28 (C=N), 129.88 (q, *J_CF_* = 32.4 Hz), 129.69, 127.21 (q, *J_CF_* = 265.9 Hz), 127.14, 125.22, 124.33, 123.45, 120.63, 118.45, 118.05, 115.88, 115.68, 107.62, 100.05, 66.26 (OCH_2_), 46.16 (NCH_2_); IR (neat) 3197 (w, NH), 2923 (m), 2313 (w), 1608 (s, C=O), 1528 (m), 1459 (s), 1281 (m), 1113 (s), 751 (s) cm^−1^; HRMS (ESI-TOF) *m/z* [M]^+^ calcd for C_25_H_20_F_3_N_3_O_2_S 483.1228, found 483.1229.

#### 4.5.10. Morpholino(5-[3-(1*H*-pyrazol-3-yl)phenyl]-1-methyl-1H-pyrrol-2-yl)methanone (**21j**)

The benzaldehyde **18c** (82.0 mg, 0.272 mmol, 1.0 equiv) was added to a solution of TsNHNH_2_ (61.4 mg, 0.329 mmol, 1.2 equiv) in CH_3_CN. After the mixture was stirred at 25 °C for 3.0 h, a solution of NaOH (330 µL, 1.36 mmol, 5.0 equiv) was added and the mixture was stirred for another 20 min. Trimethylsilyl acetylene (**5**, 81.0 mg, 0.816 mmol, 3.0 equiv) was then added and the mixture was stirred at 50 °C for 45 h. The volatiles in the reaction mixture were evaporated under reduced pressure and the residue was dissolved in a mixture of water–ethyl acetate (1:1, 50–60 mL). The solution was extracted with EtOAc (3 × 15 mL) and the combined organic layers were then dried over MgSO_4_ (s), filtered, and concentrated under reduced pressure to afford a residue. The residue (0.112 g, 0.275 mmol, 1.0 equiv) was dissolved in dry THF and added TBAF (71.9 mg, 0.275 mmol, 1.0 equiv). After the mixture was stirred at 25 °C for 3.0 h, it was quenched with quenched with water. The solution was extracted with EtOAc (3 × 15 mL) and the combined organic layers were then dried over MgSO_4_ (s), filtered, and concentrated under reduced pressure to afford a residue. The residue was purified by use of column chromatography packed with silica gel to give the pure polycycle **21j** (83.4 mg, 0.247 mmol) in 90% yield as pale yellow oil: TLC R*_f_* 0.48 (60% EtOAc as the eluent); ^1^H-NMR (CDCl_3_) *δ* 7.76 (s, 1 H, ArH), 7.72 (d, *J* = 7.2 Hz, 1 H, ArH), 7.52 (s, 1 H, pyrrole), 7.38 (t, *J* = 7.2 Hz, 1 H, ArH), 7.29 (d, *J* = 7.2 Hz, 1 H, ArH), 6.57 (s, 1 H, pyrrole), 6.35 (d, *J* = 2.8 Hz, 1 H, pyrazole), 6.16 (d, *J* = 2.8 Hz, 1 H, pyrazole), 3.78 (s, 3 H, CH_3_), 3.78–3.69 (m, 8 H, 4 × CH_2_); ^13^C-NMR (CDCl_3_) *δ* 163.22 (C=O), 148.84 (C=N), 138.65, 132.71, 132.59, 131.96, 128.76, 128.54, 126.53, 126.06, 124.94, 112.80, 107.97, 102.58, 66.94 (OCH_2_), 45.64 (NCH_2_), 33.85 (NCH_3_); IR (neat) 3419 (s, NH), 2115 (w, aromatic C–H bending), 1621 (m, C=O), 1456 (m), 1273 (w), 1113 (w), 1034 (w), 764 (w) cm^−1^; HRMS (ESI-TOF) *m/z* [M]^+^ calcd for C_19_H_20_N_4_O_2_ 336.1587, found 336.1586.

### 4.6. Morpholino(5-[3-(1-methyl-1H-pyrazol-3-yl)phenyl]furan-2-yl)methanone (***23***)

To a solution containing a polycycle **21a** (55.0 mg, 0.170 mmol, 1.0 equiv) in DMF (2.0 mL) was added CH_3_I (28.9 mg, 0.204 mmol, 1.2 equiv) and K_2_CO_3_ (35.1 mg, 0.255 mmol, 1.5 equiv). After the reaction mixture was stirred at 25 °C for 3.0 h, it was quenched with water (20 mL). The solution was extracted with EtOAc (3 × 15 mL) and the combined organic layers were then dried over MgSO_4_ (s), filtered, and concentrated under reduced pressure to afford a residue. The residue was purified by use of column chromatography to give the pure polycycle **23** (56.4 mg, 0.149 mmol) in 88% yield as colorless oil: TLC R*_f_* 0.58 (60% EtOAc as the eluent); ^1^H-NMR (CDCl_3_) *δ* 8.09 (s, 1 H, ArH), 7.72 (d, *J* = 7.8 Hz, 1 H, ArH), 7.58 (d, *J* = 7.8 Hz, 1 H, ArH), 7.41–7.39 (m, 2 H, ArH + pyrazole), 7.12 (d, *J* = 3.6 Hz, 1 H, furan), 6.78 (d, *J* = 3.6 Hz, 1 H, furan), 6.55 (d, *J* = 2.4 Hz, 1 H, pyrazole), 3.95 (s, 3 H, NCH_3_), 3.90–3.79 (m, 4 H, 2 × OCH_2_), 3.78–3.77 (m, 4 H, 2 × NCH_2_); ^13^C-NMR (CDCl_3_) *δ* 158.48 (C=O), 155.94, 155.52, 146.98 (C=N), 138.81, 134.88, 131.44, 128.94, 125.52, 123.08, 121.13, 118.81, 106.55, 102.75, 66.74 (OCH_2_), 45.72 (NCH_2_), 37.80 (NCH_3_); IR (neat) 3461 (w), 2856 (m), 1700 (s), 1618 (s, C=O), 1436 (s), 1278 (s), 1115 (s), 1028 (s), 794 (m) cm^−1^; HRMS (ESI-TOF) *m/z* [M]^+^ calcd for C_19_H_19_N_3_O_3_ 337.1426, found 337.1423.

## 5. Conclusions

In conclusion, eighteen conjugated compounds with the basic skeleton of (morpholine, cyclic amine, or aryl amine)–(furan, thiophene, or pyrrole)–benzene–1*H*-pyrazole were synthesized through two different chemical pathways. Their inhibitory activity toward enteroviruses was investigated. Conjugates **10c**, **21h**, and **21i** were able to impede EV71 at EC_50_ values of 6.16, 4.10, and 2.29 μM, respectively (see Figure S1 in Supplementary Materials). These three leads showed ideal lipophilicity and water solubility, which fit into the suggested physical properties of pharmaceuticals. The best SI value of 33.4 was associated with the new compound **21i**. Its skeleton contains three heterocyclic rings (i.e., morpholine, thiophene, and pyrazole), two benzene rings, one –CF_3_ group, one amide linker, and three hinged C–C bonds. Accordingly, we identify compound **21i** as a lead with higher antiviral activity and lower cytotoxicity in vitro than the original hit compound **21a**.

## Figures and Tables

**Table 1:** Antiviral activity of polycyclic compounds on the replication of EV71 strain BrCr in RD cells.

Conjugates	Structure	CC_50_^a^ (µM)	EC_50_^b^ (µM)	SI ^c^	log *P* ^d^
**10a**	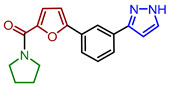	119.0 ± 2.0	16.2 ± 1.0	7.30	3.42
**10b**	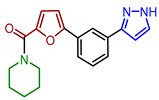	14.3 ± 1.0	2.14 ± 0.12	6.70	3.87
**10c**	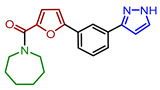	81.1 ± 3.0	6.16 ± 0.15	13.1	4.22
**12a**	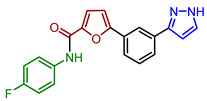	4.53 ± 0.10	<1.73	2.60	4.18
**12b**	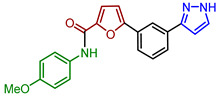	21.0 ± 1.5	2.41 ± 0.30	8.70	4.15
**13**	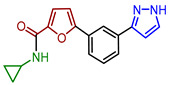	ND	15.1 ± 1.0	ND	3.25
**14**	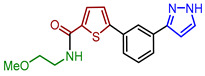	22.4 ± 2.5	ND	ND	3.15
**21a**	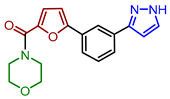	ND	11.6 ± 0.3	ND	2.60
**21b**	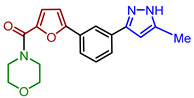	ND	13.0 ± 2.5	ND	2.87
**21c**	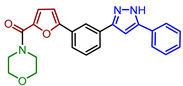	4.13 ± 0.12	ND	ND	3.92
**21d**	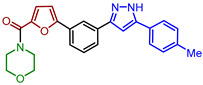	3.25 ± 0.30	ND	ND	4.30
**21e**	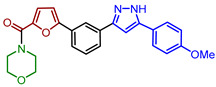	2.09 ± 0.10	<1.40	1.50	3.96
**21f**	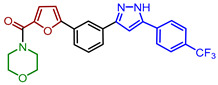	3.28 ± 0.11	<1.28	2.60	4.33
**21g**	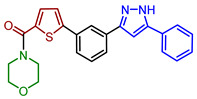	ND	241.1 ± 3.0	ND	4.61
**21h**	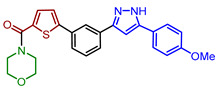	41.9 ± 2.2	4.10 ± 0.12	10.2	4.64
**21i**	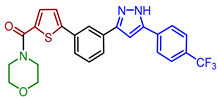	76.4 ± 3.0	2.29 ± 0.10	33.4	5.04
**21j**	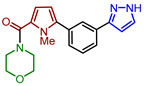	ND	223.0 ± 2.0	ND	2.56
**23**	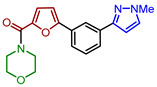	ND	34.6 ± 1.0	ND	2.75
	Pirodavir ^e^	>54	0.3 ± 0.1	>180	4.44

^a^ The concentration of a compound with an adverse effect of 50% was observed on the host cell metabolism, as determined by the MTS method. ^b^ The concentration of a compound at which virus replication was inhibited by 50% was observed, as determined by real-time quantitative RT–PCR. ^c^ Selectivity index. ^d^ Log *P* values were determined as described in the text and were an average of three independent experiments. ^e^ Pirodavir is used as a positive control. ND = Not Determined.

## Supplementary Materials

The following are available online. Table S1: Comparison of EC_50_ values among compounds **10c**, **21h**, and **21i**; ^1^H-NMR and ^13^C-NMR spectra. CCDC-2019249 contains the supplementary crystallographic data for morpholino(5-[3-(5-phenyl-1*H*-pyrazol-3-yl)phenyl]thiophen-2-yl)methanone (**21g**).
