# Supplementary material for: Enterovirus Inhibition by Hinged Aromatic Compounds with Polynuclei
Source: Molecules. 2020 Aug 22;25(17):3821. doi: 10.3390/molecules25173821 (PMC7503712; doi:10.3390/molecules25173821)
Supplement: Supplementary file 1 [file molecules-25-03821-s001.pdf]

Supplementary Materials

Enterovirus Inhibition by Hinged Aromatic Compounds with Polynuclei

Jih Ru Hwu[a,b,c,*], Avijit Panja[a], Srinivasan Jayakumar[a], Shwu-Chen Tsay[a,b,c], Kui-Thong Tan [a,b], Wen-Chieh Huang[a,b],Yu-Chen Hu[b,d], Pieter Leyssen[e], and Johan Neyts[e,*]

[a] *Department of Chemistry, National Tsing Hua University, Hsinchu 300, Taiwan*

[b] *Frontier Research Center on Fundamental and Applied Sciences of Matters, National Tsing Hua University, Hsinchu 300, Taiwan*

[c] *Department of Chemistry, National Central University, Jhongli City, Taoyuan 320, Taiwan*

[d] *Department of Chemical Engineering, National Tsing Hua University, Hsinchu 300, Taiwan*

[e] *Rega Institute for Medical Research, Katholieke Universiteit Leuven, Minderbroedersstraat 10, B-3000 Leuven, Belgium*

Contents

^{1}H NMR and ^{13}C NMR Spectra of New Compounds S2

Comparison of EC_{50} values among compounds **10c**, **21h**, and **21i** S27

X-ray Crystal Data of Compound **21g** S28

^{1}H NMR and ^{13}C NMR Spectra of New Compounds

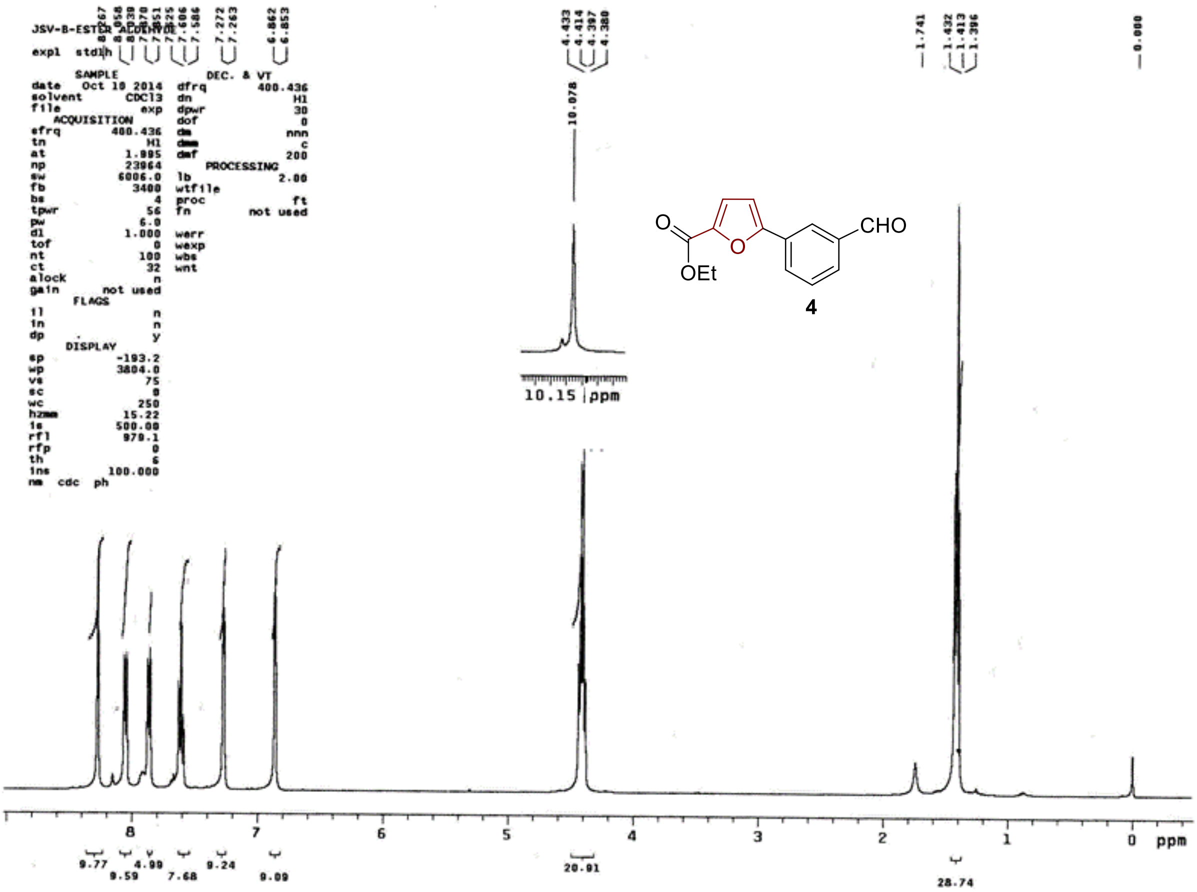

^{1}H NMR spectrum of compound **4**

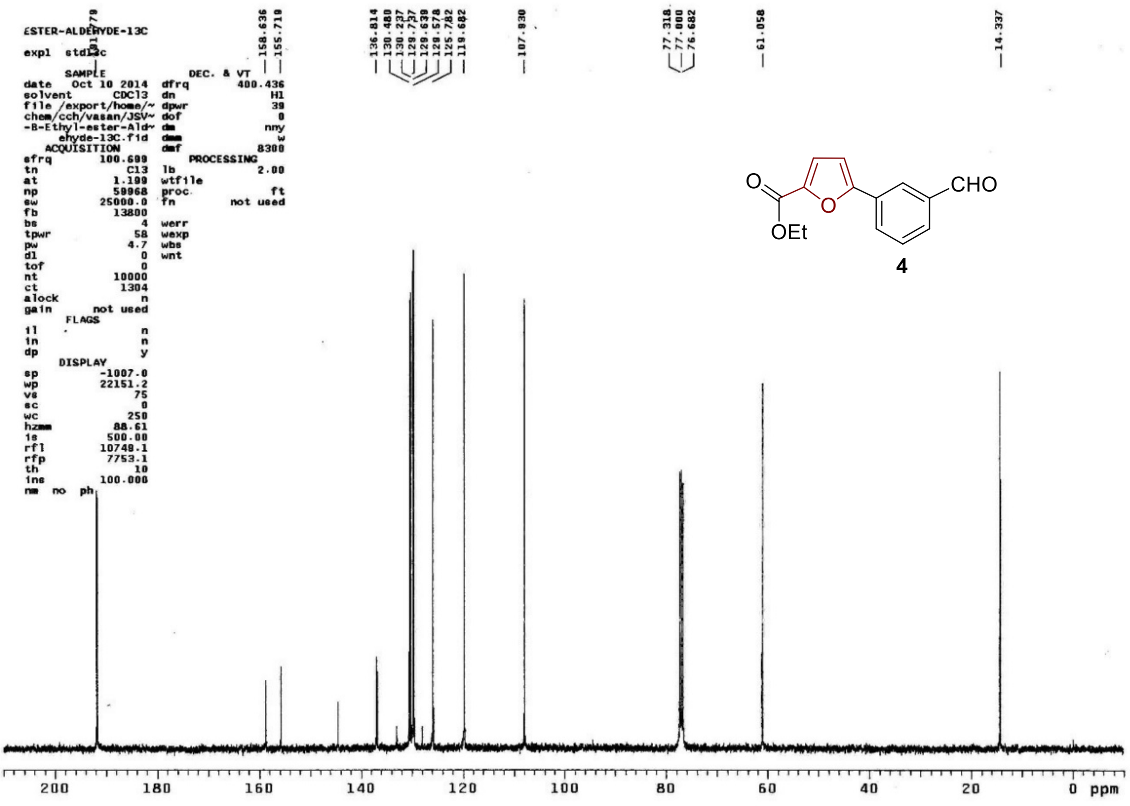

^{13}C NMR spectrum of compound **4**

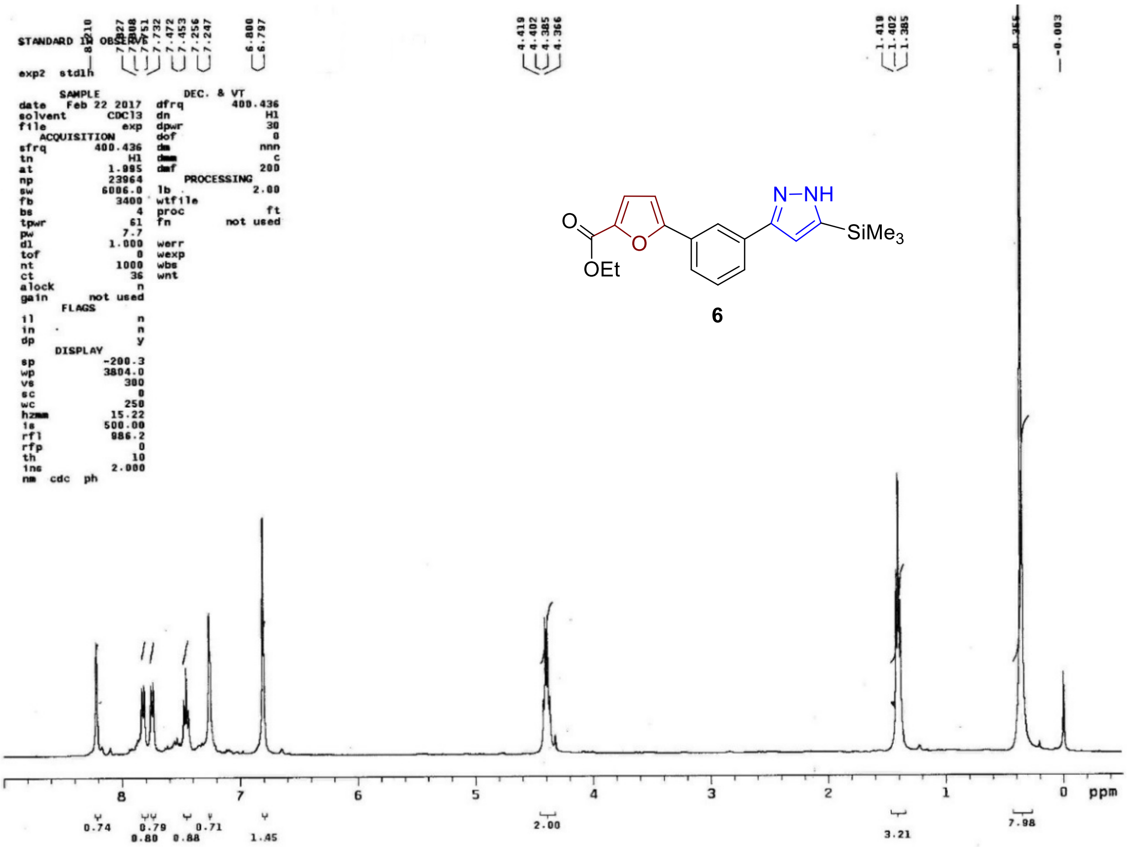

^{1}H NMR spectrum of compound **6**

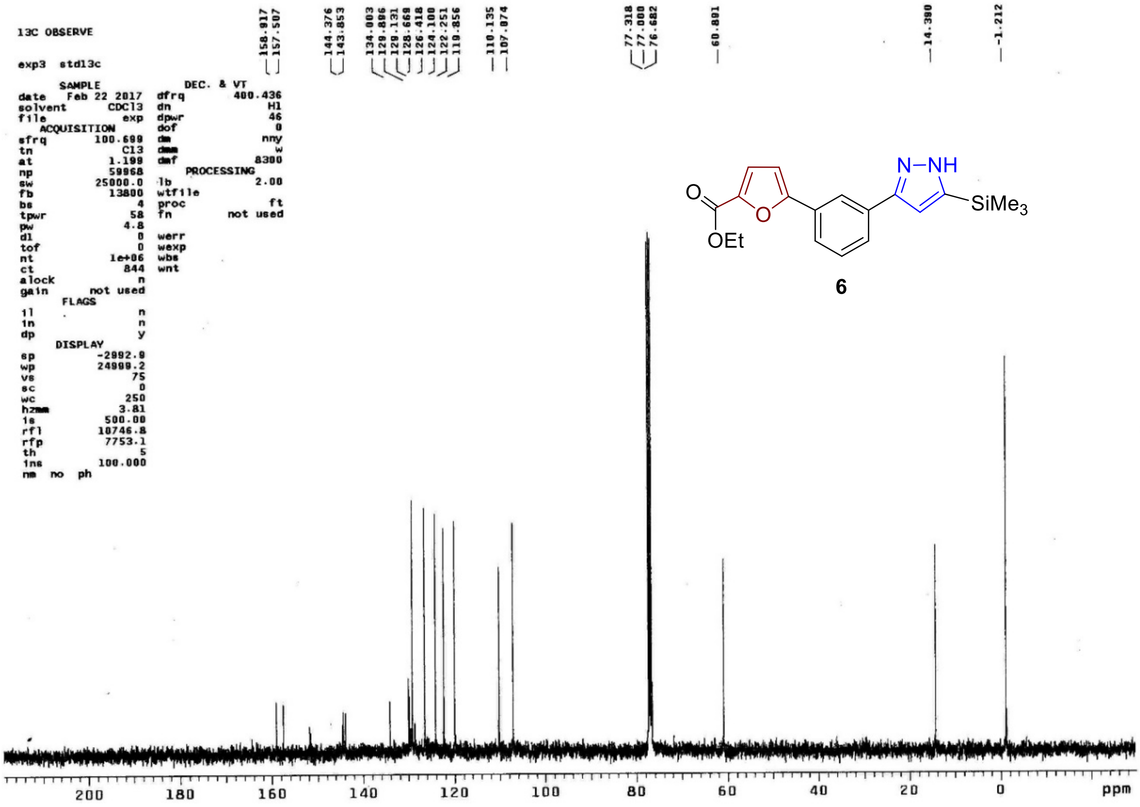

^{13}C NMR spectrum of compound **6**

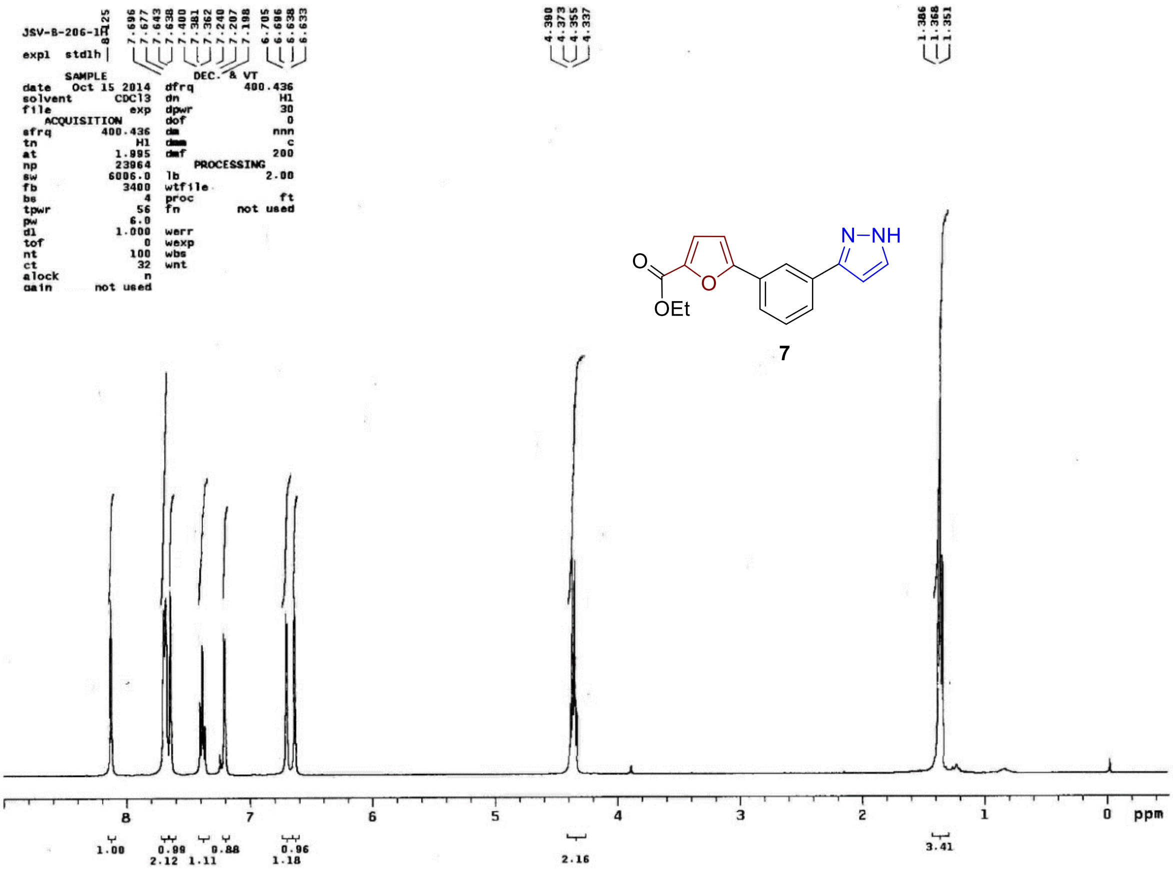

^{1}H NMR spectrum of compound **7**

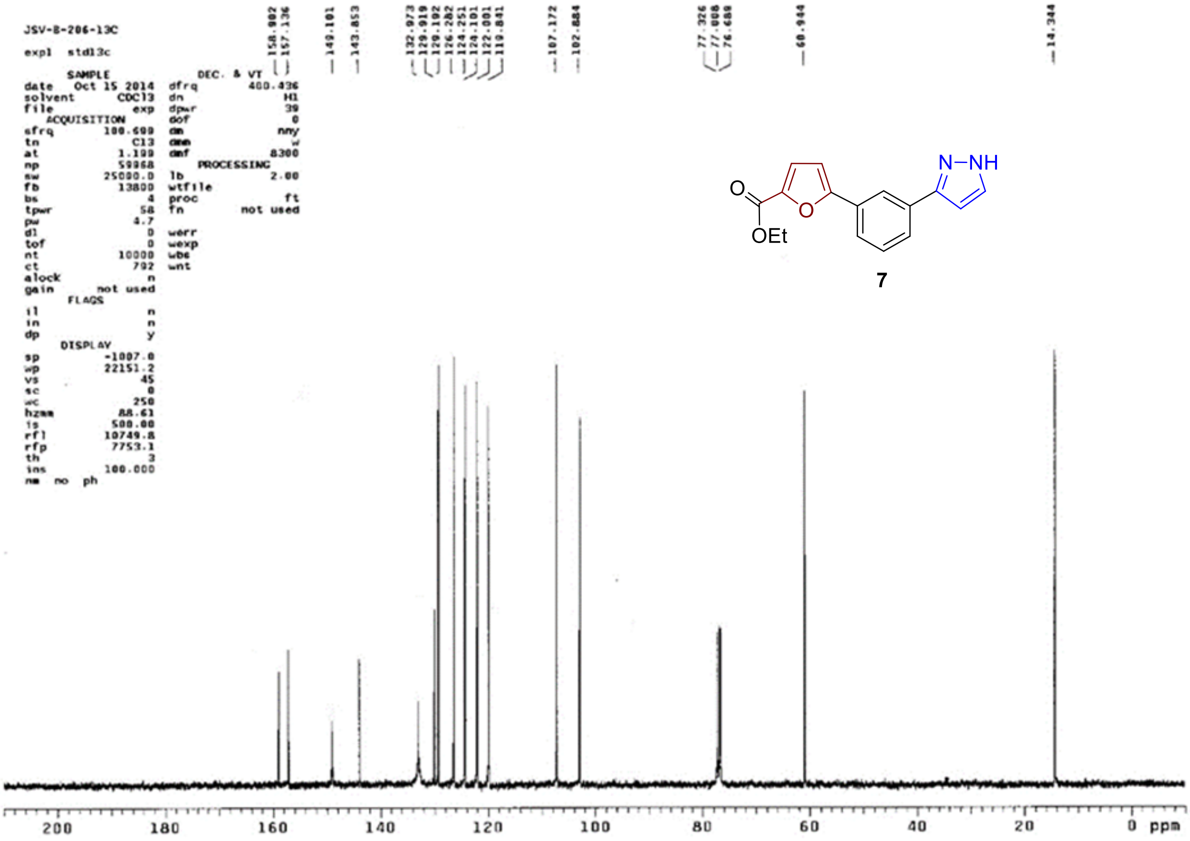

^{13}C NMR spectrum of compound **7**

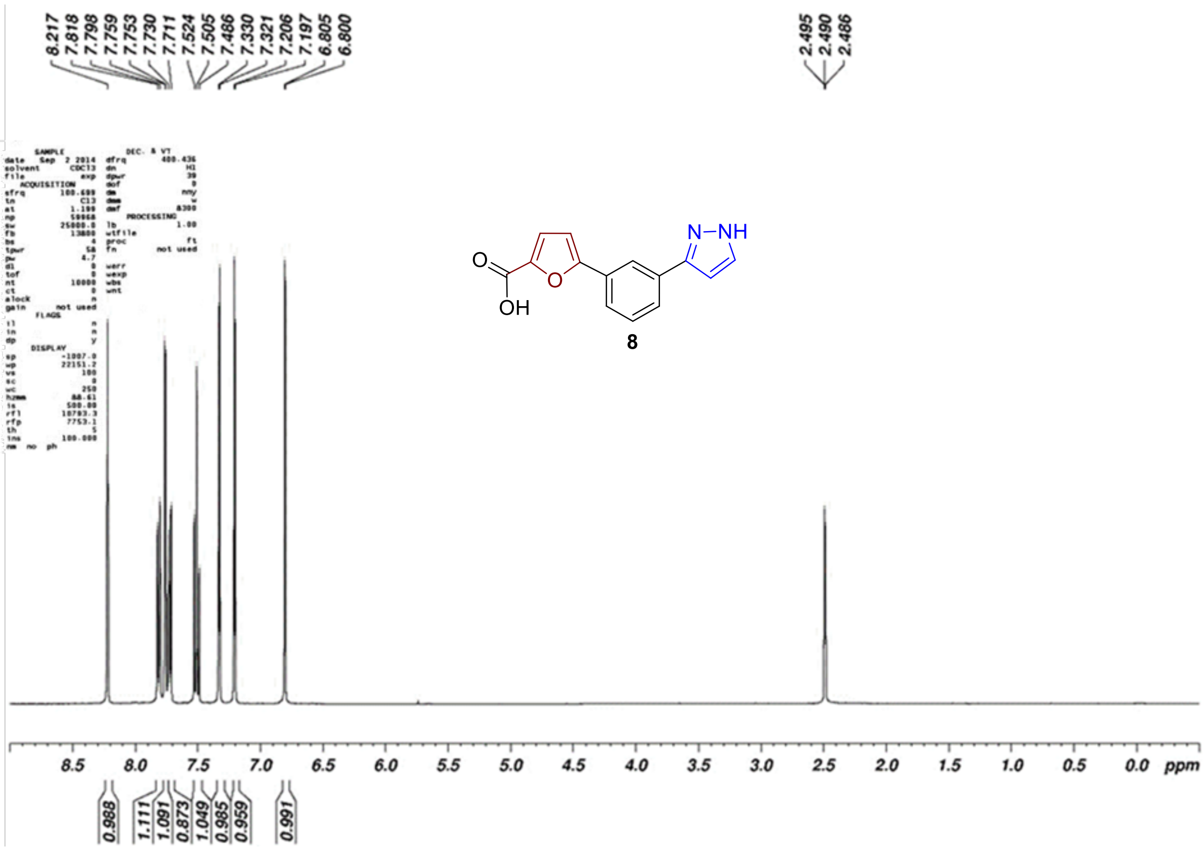

^{1}H NMR spectrum of compound **8**

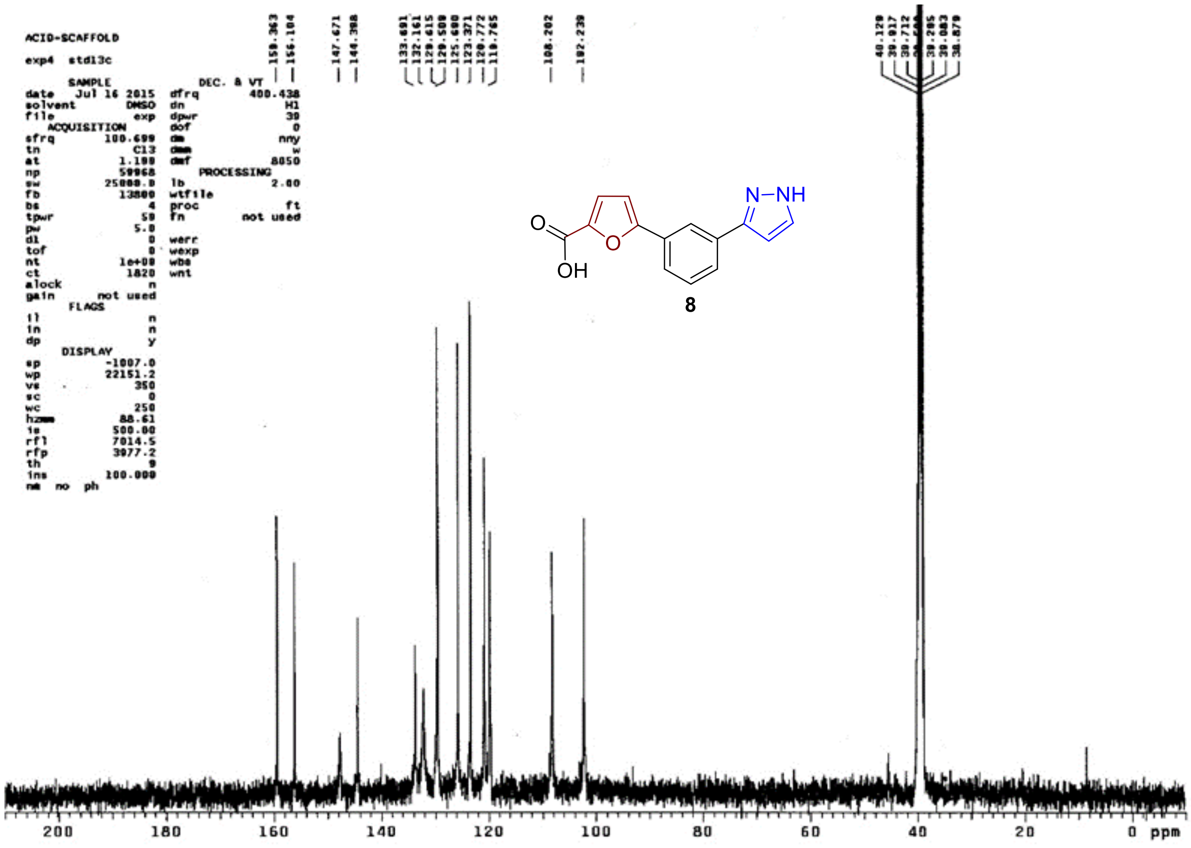

^{13}C NMR spectrum of compound **8**

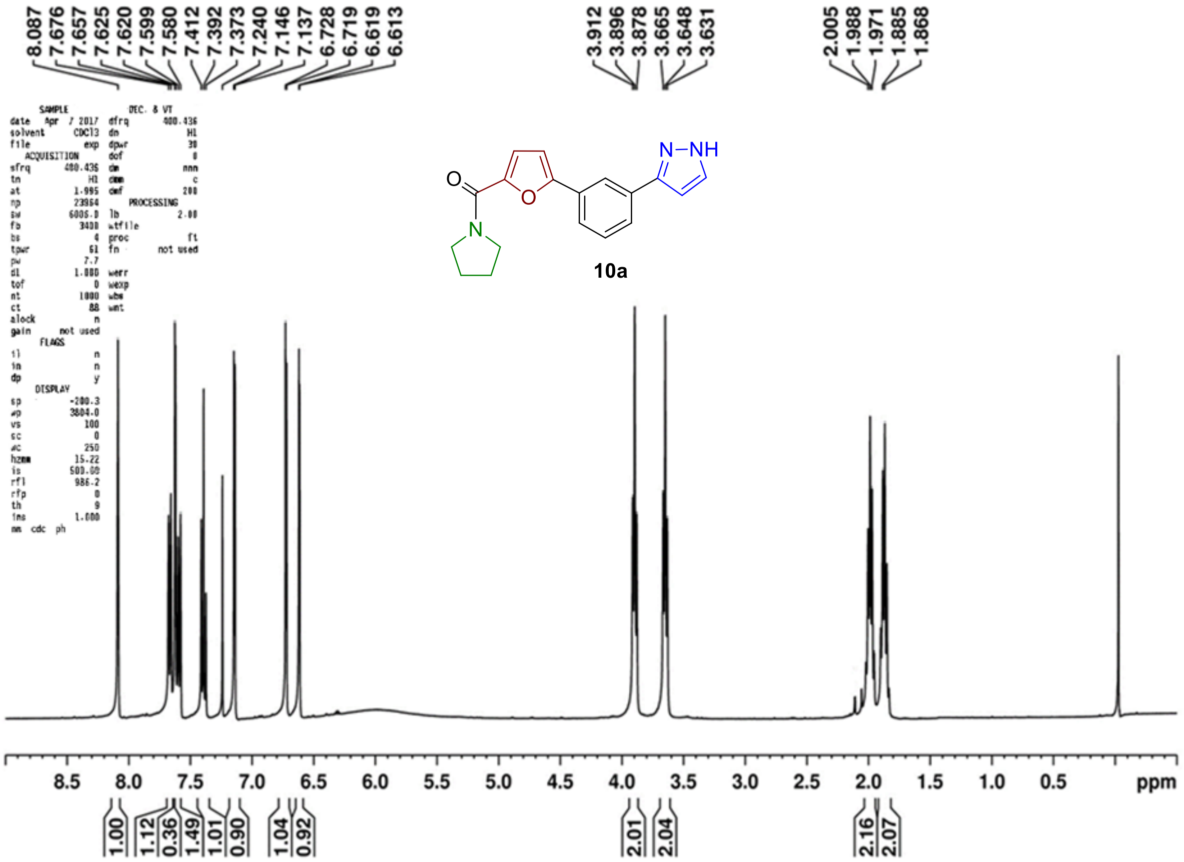

^{1}H NMR spectrum of compound **10a**

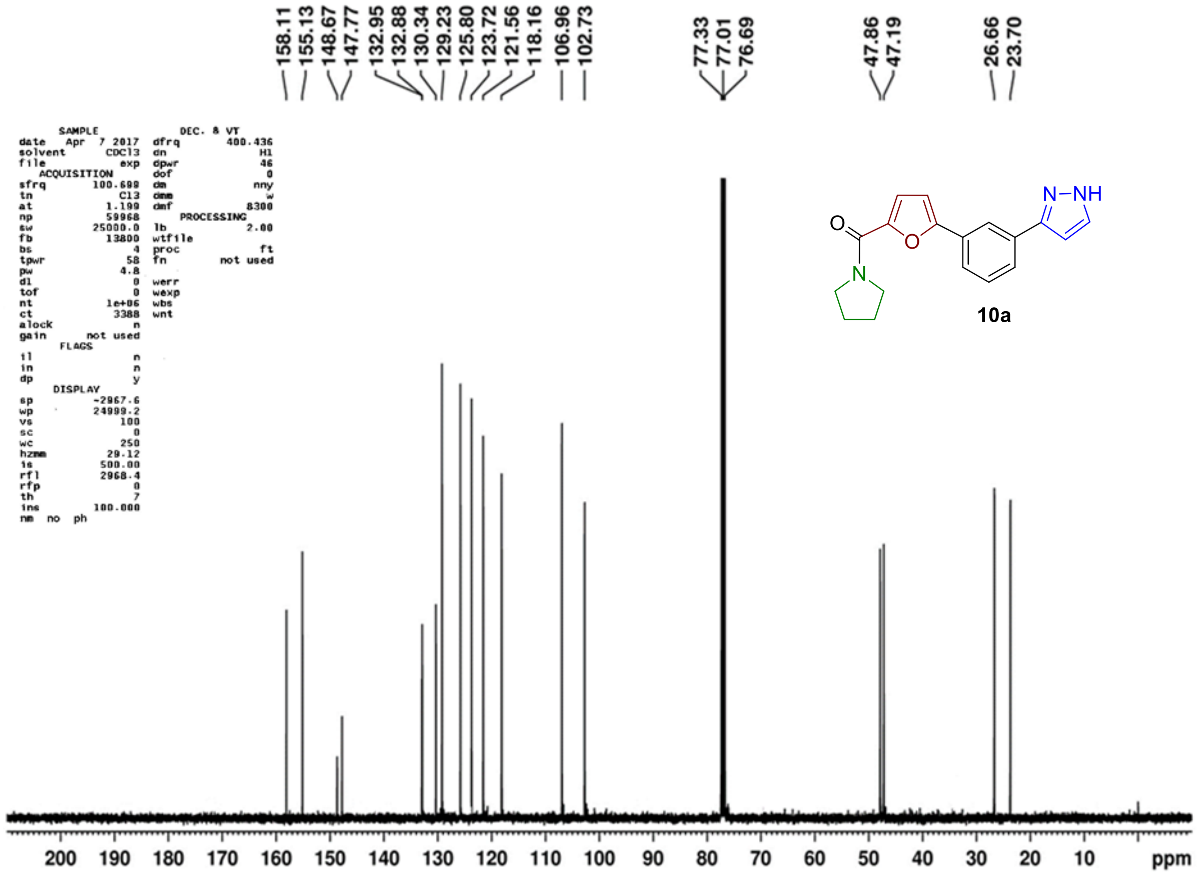

^{13}C NMR spectrum of compound **10a**

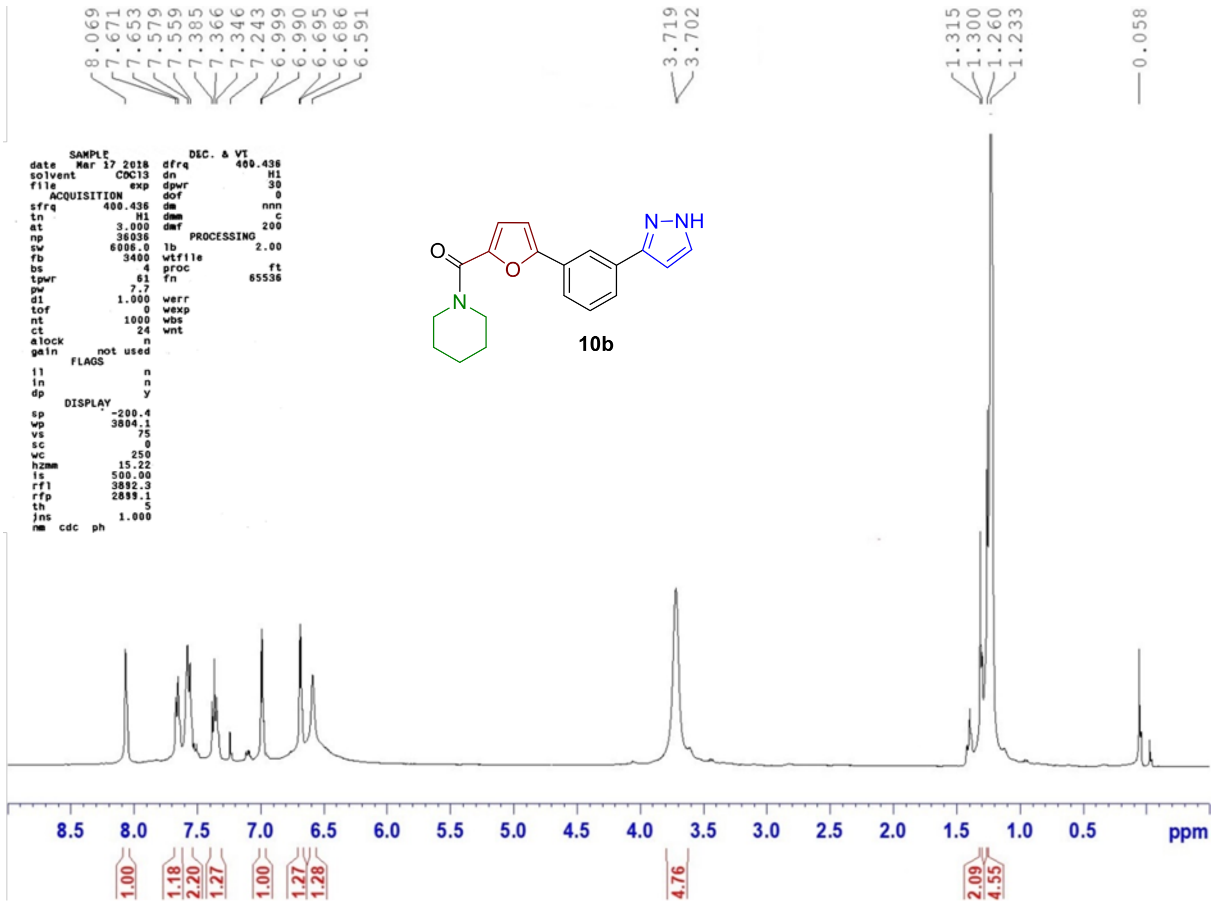

^{1}H NMR spectrum of compound **10b**

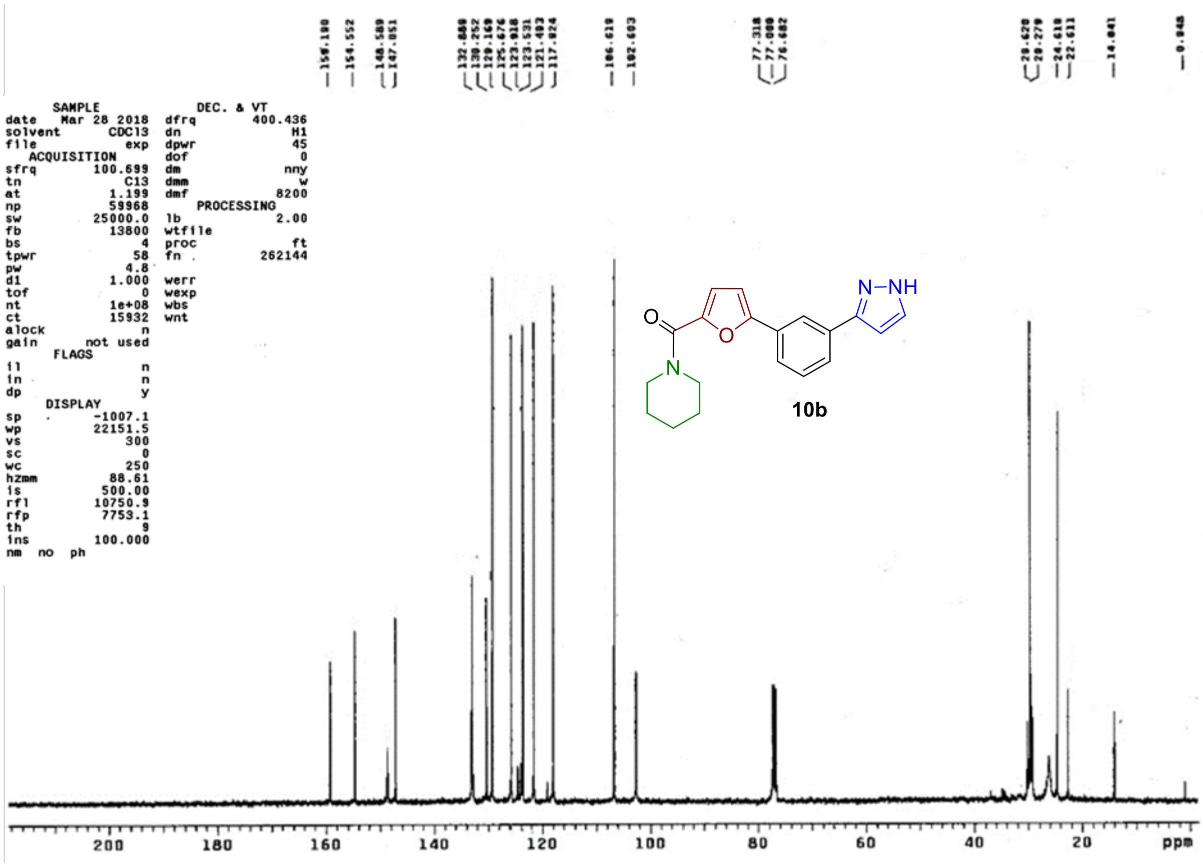

^{13}C NMR spectrum of compound **10b**

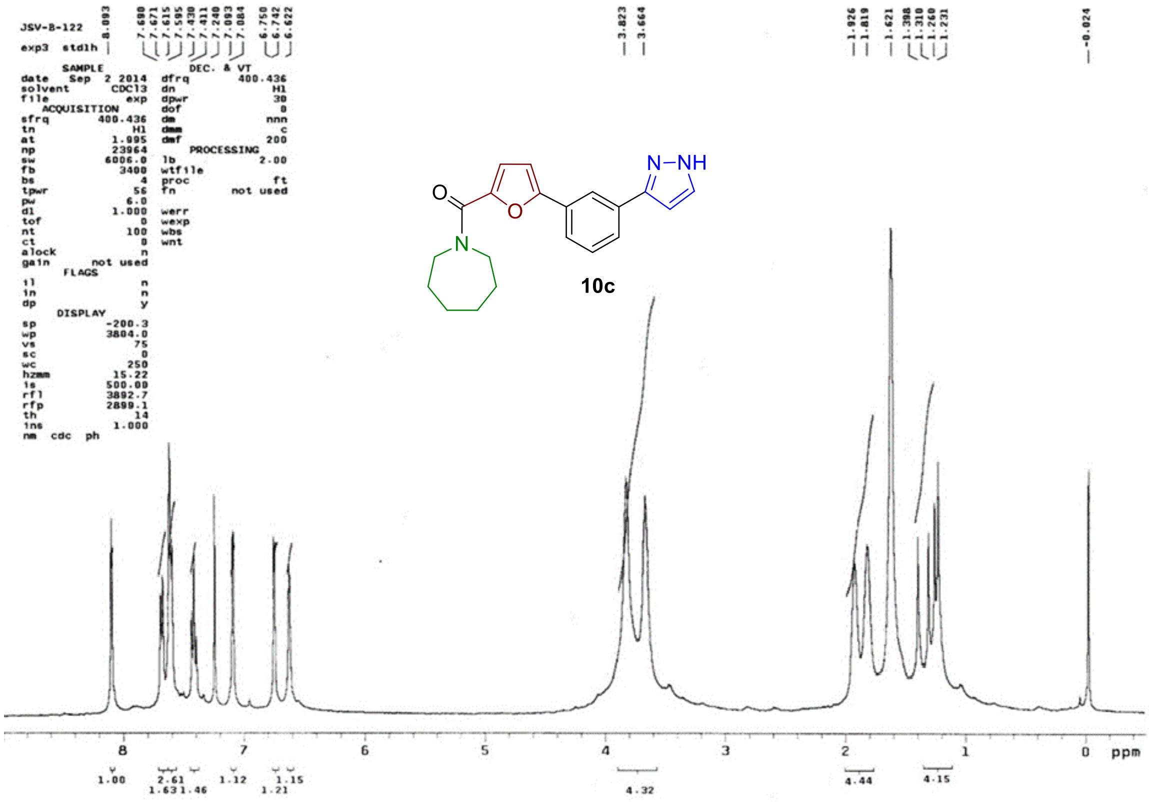

^{1}H NMR spectrum of compound **10c**

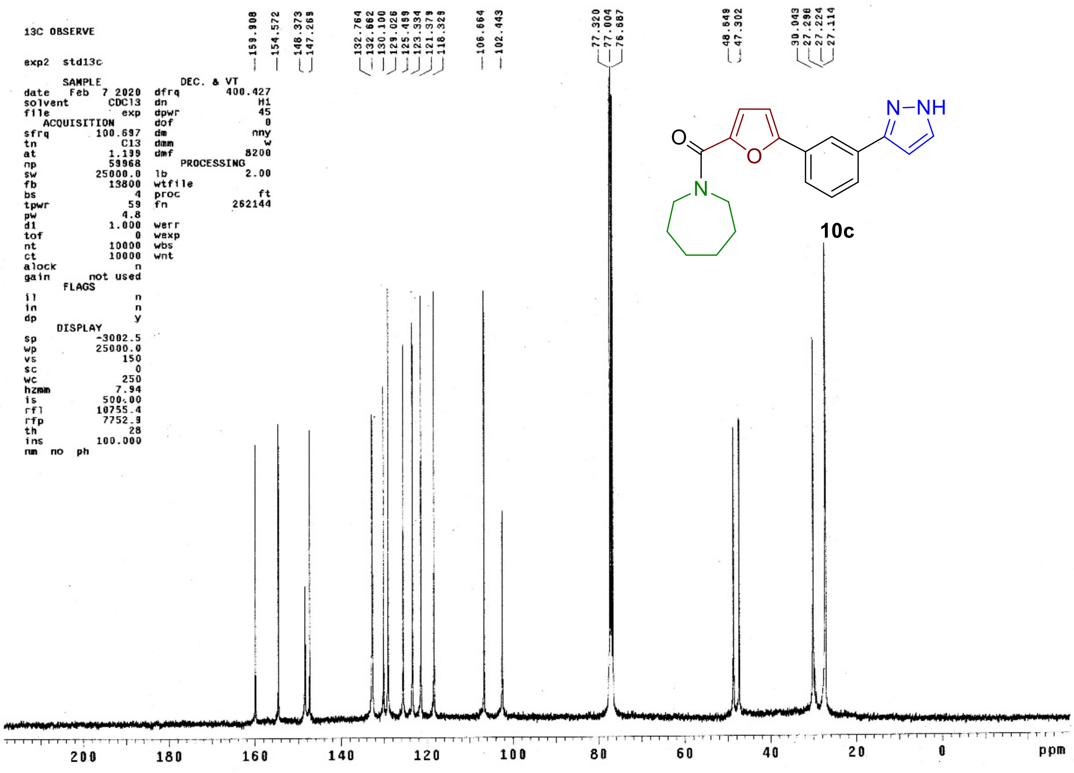

^{13}C NMR spectrum of compound **10c**

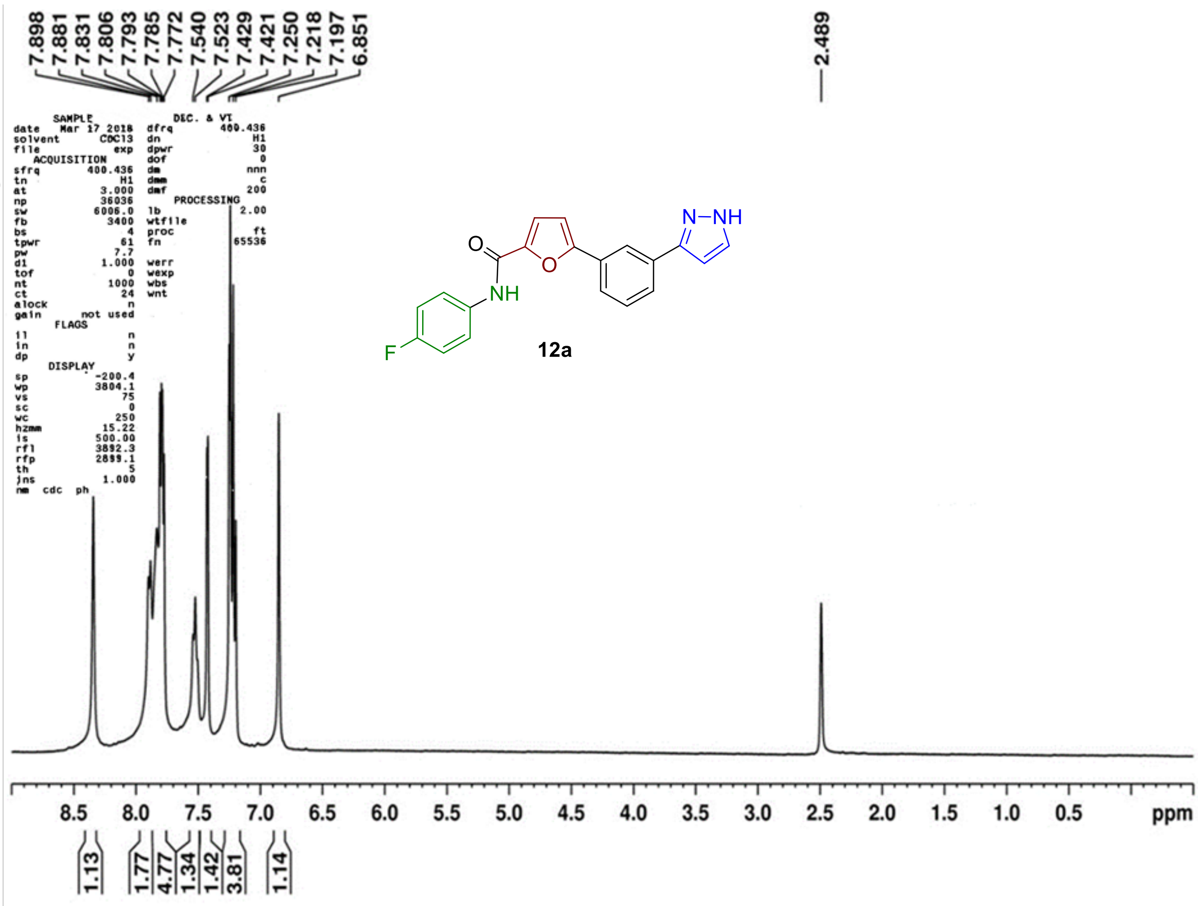

^{1}H NMR spectrum of compound **12a**

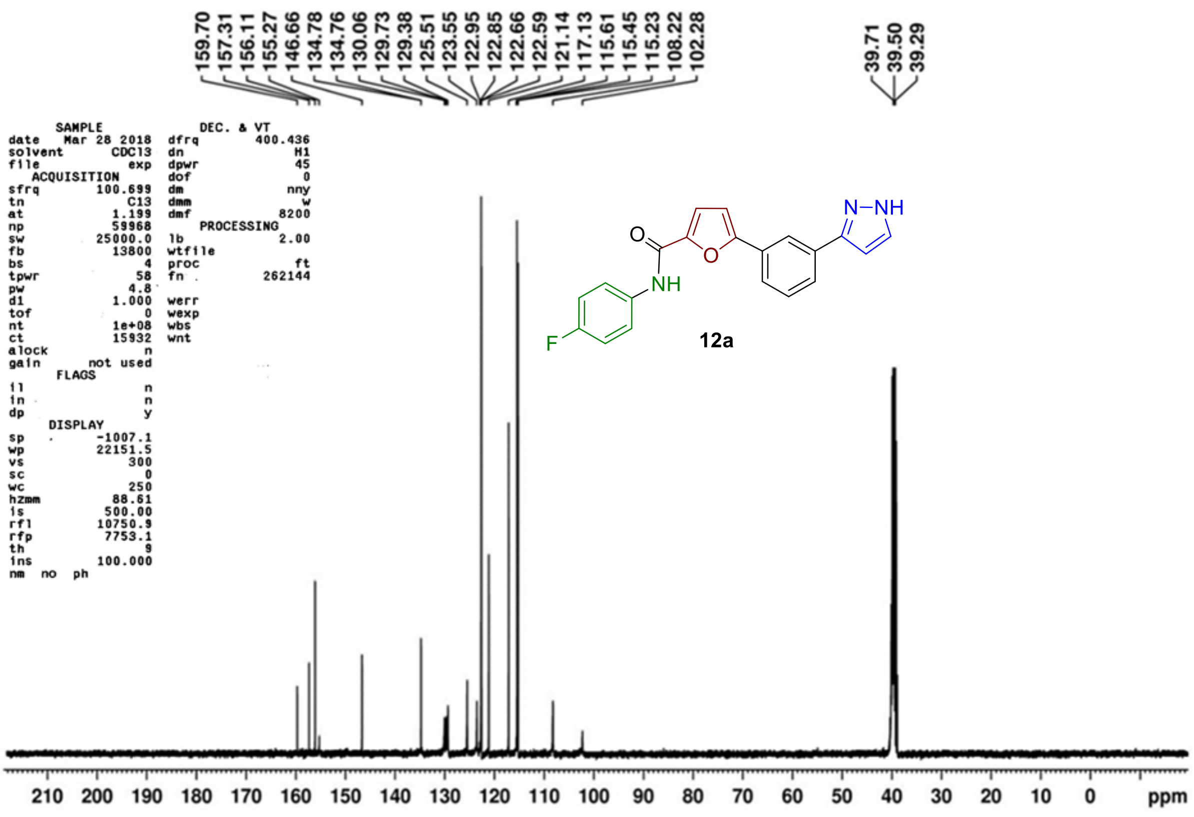

^{13}C NMR spectrum of compound **12a**

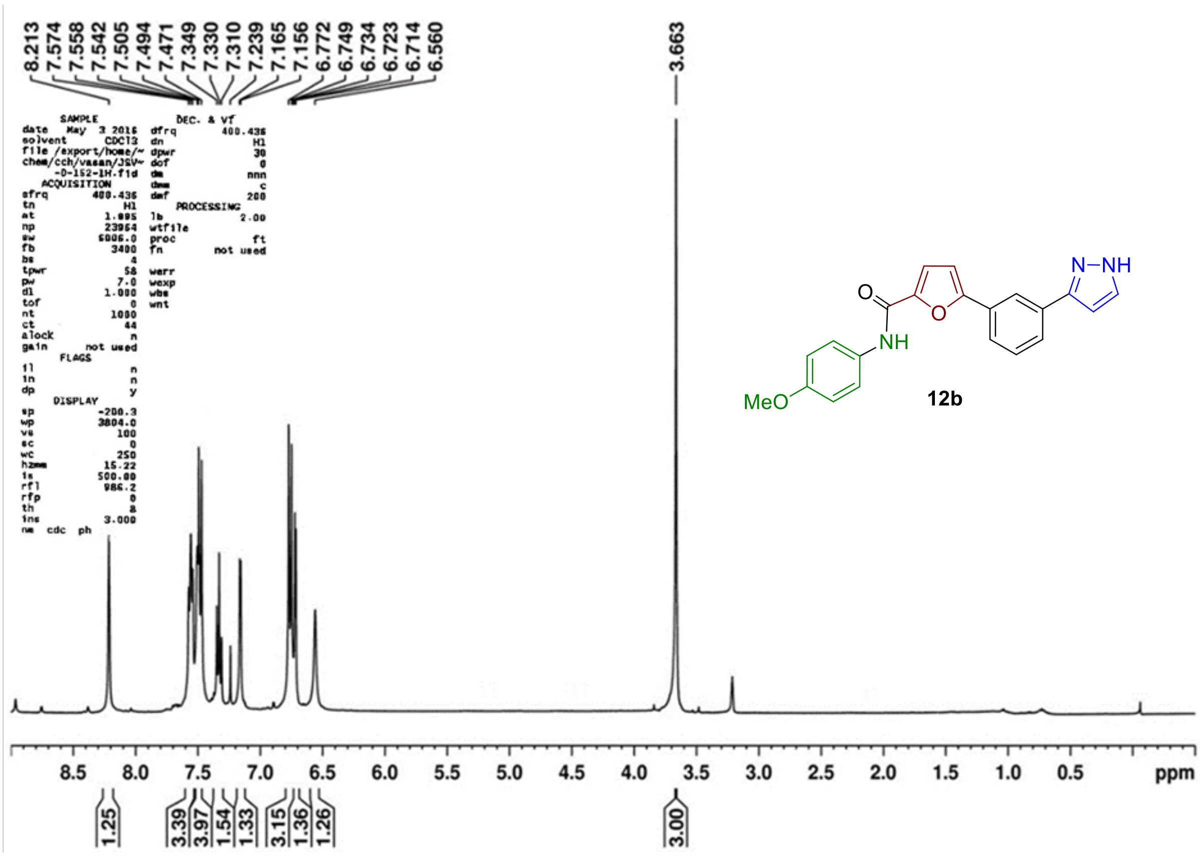

^{1}H NMR spectrum of compound **12b**

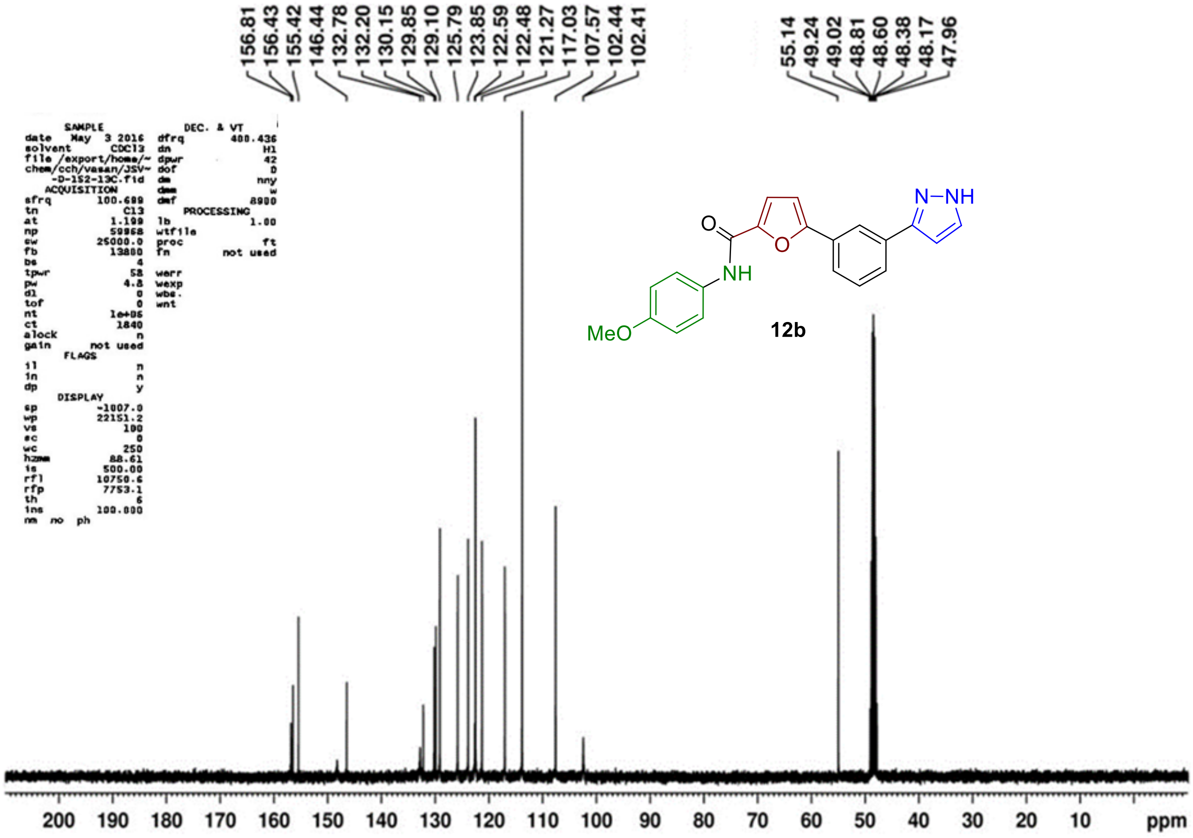

^{13}C NMR spectrum of compound **12b**

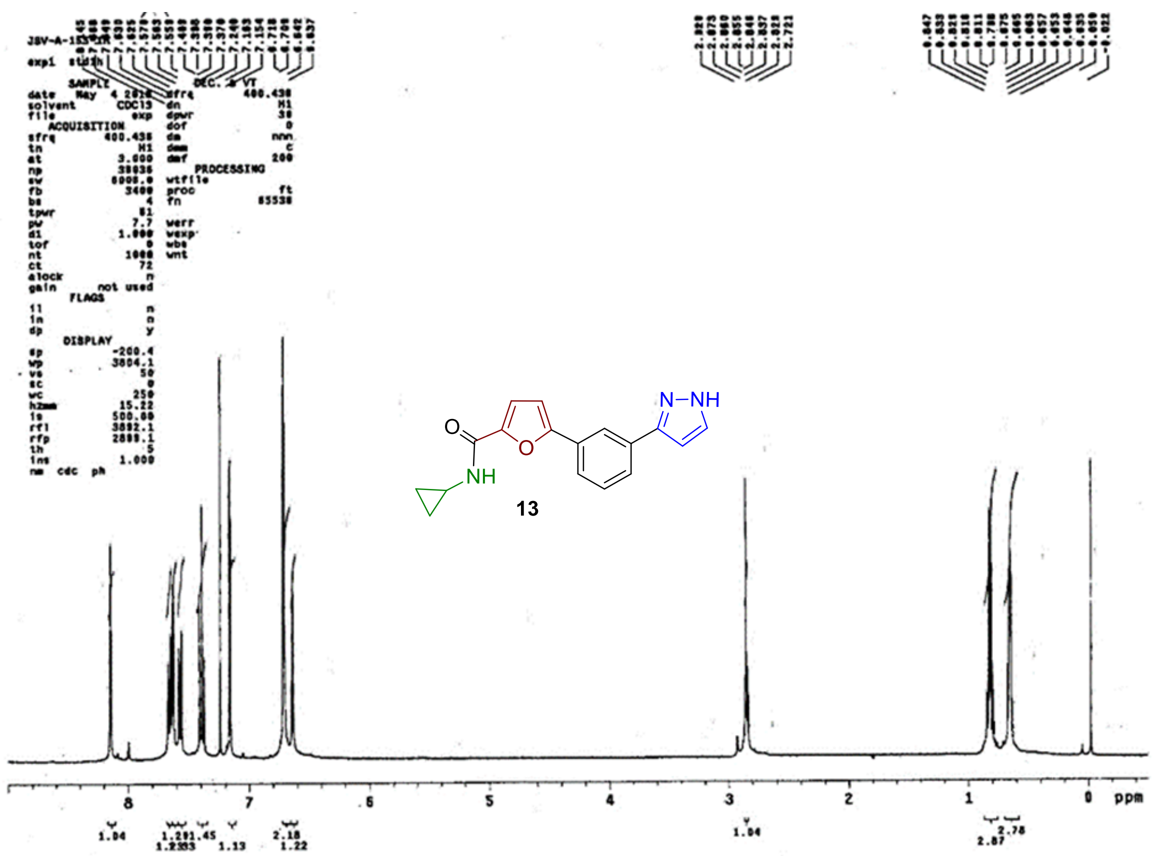

^{1}H NMR spectrum of compound **13**

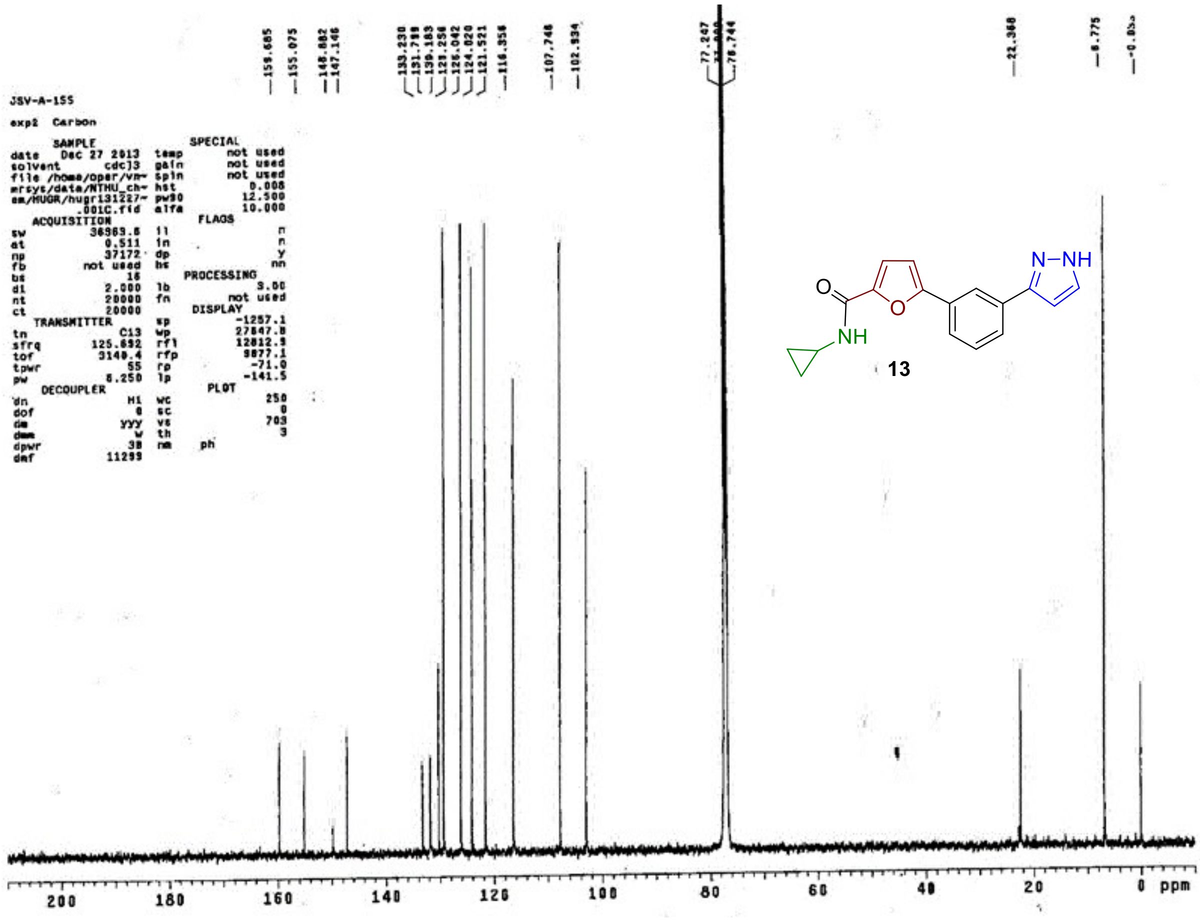

^{13}C NMR spectrum of compound **13**

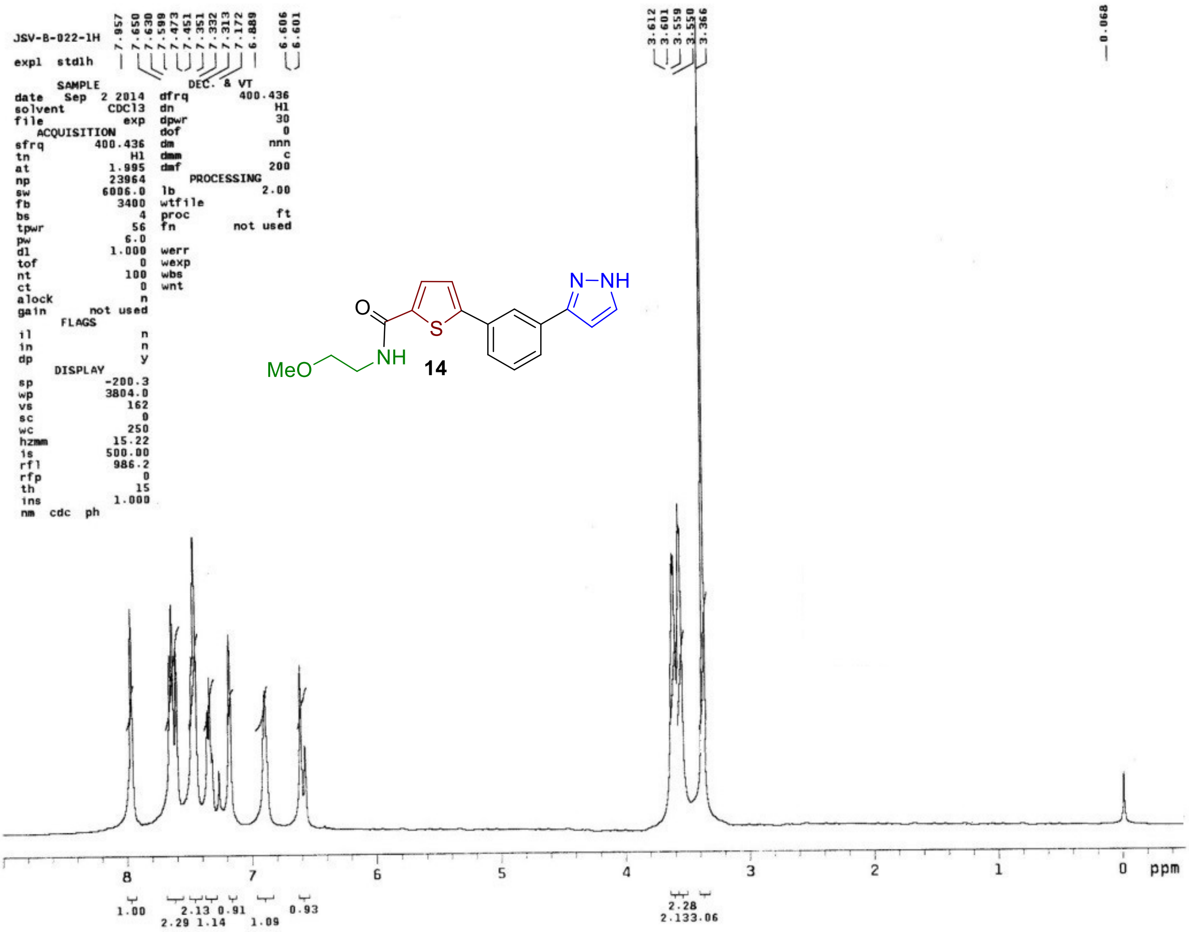

^{1}H NMR spectrum of compound **14**

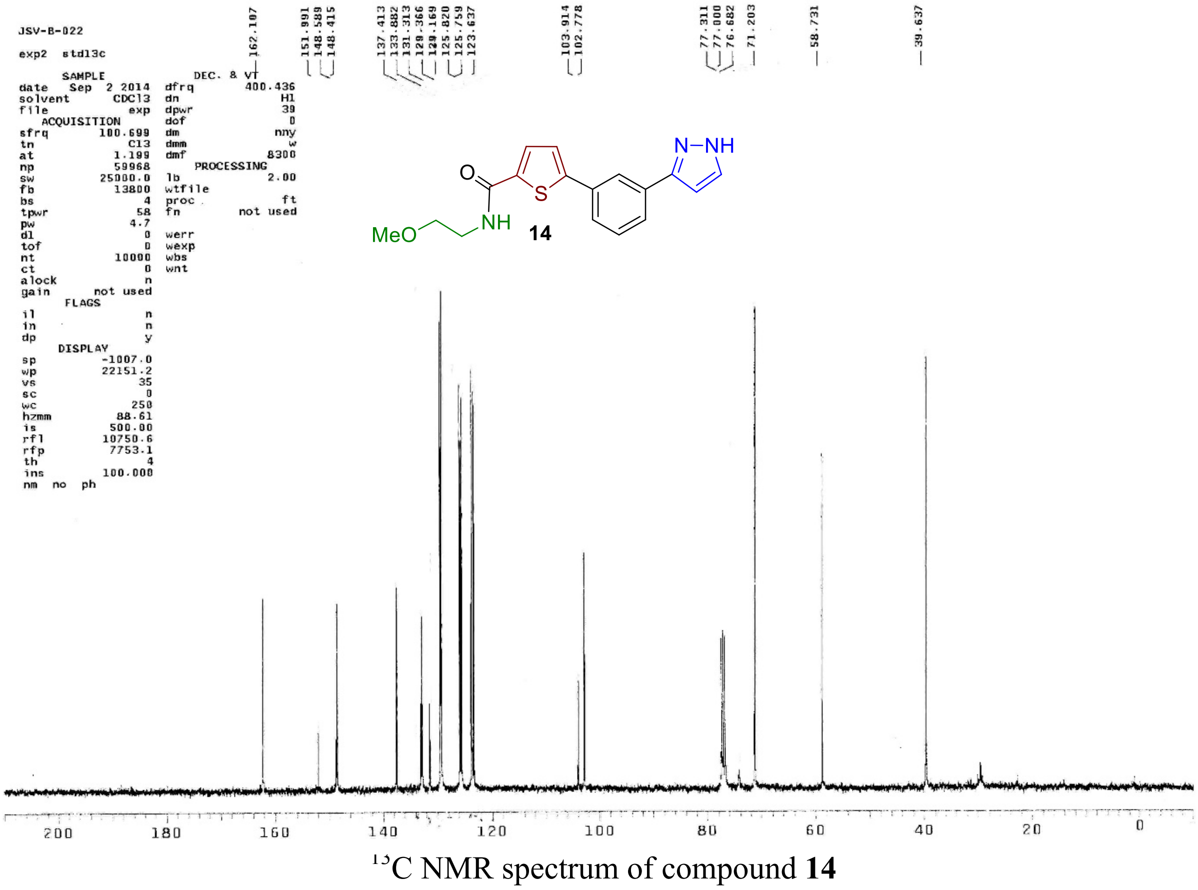

^{13}C NMR spectrum of compound **14**

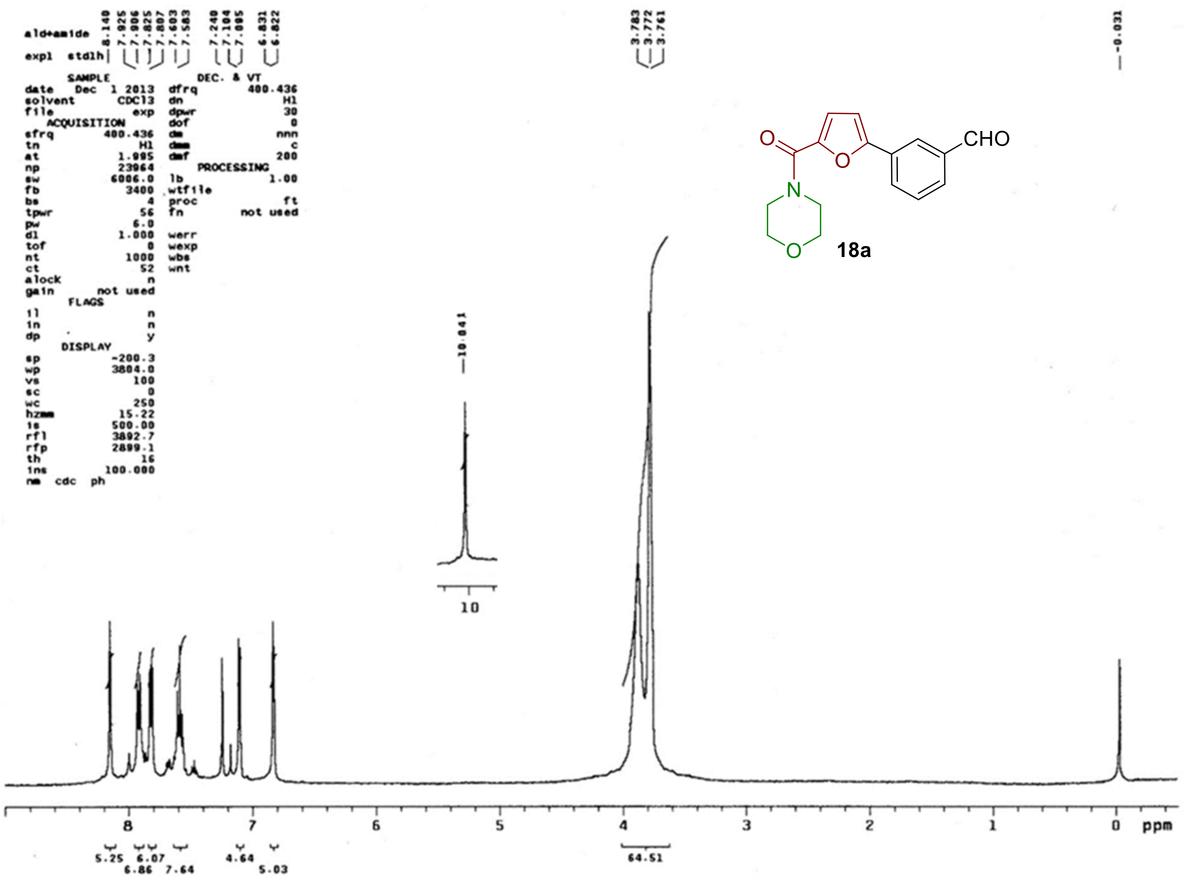

^{1}H NMR spectrum of compound **18a**

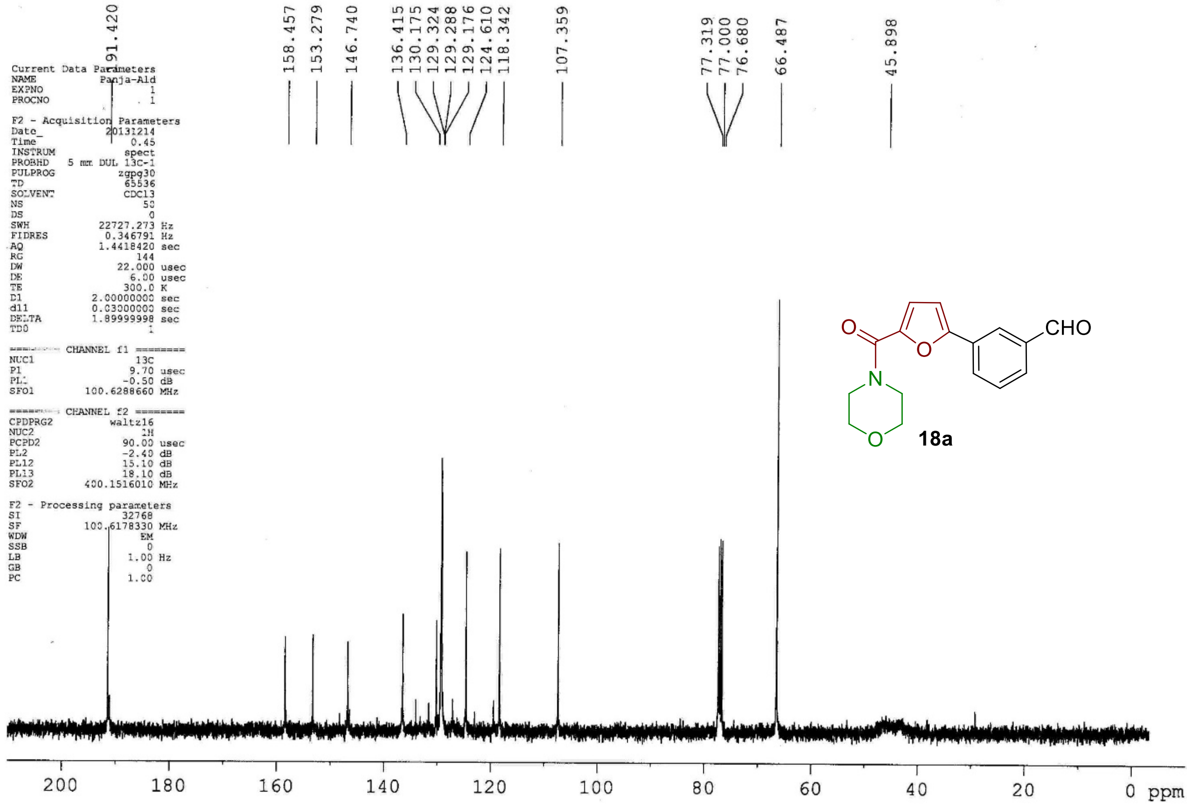

^{13}C NMR spectrum of compound **18a**

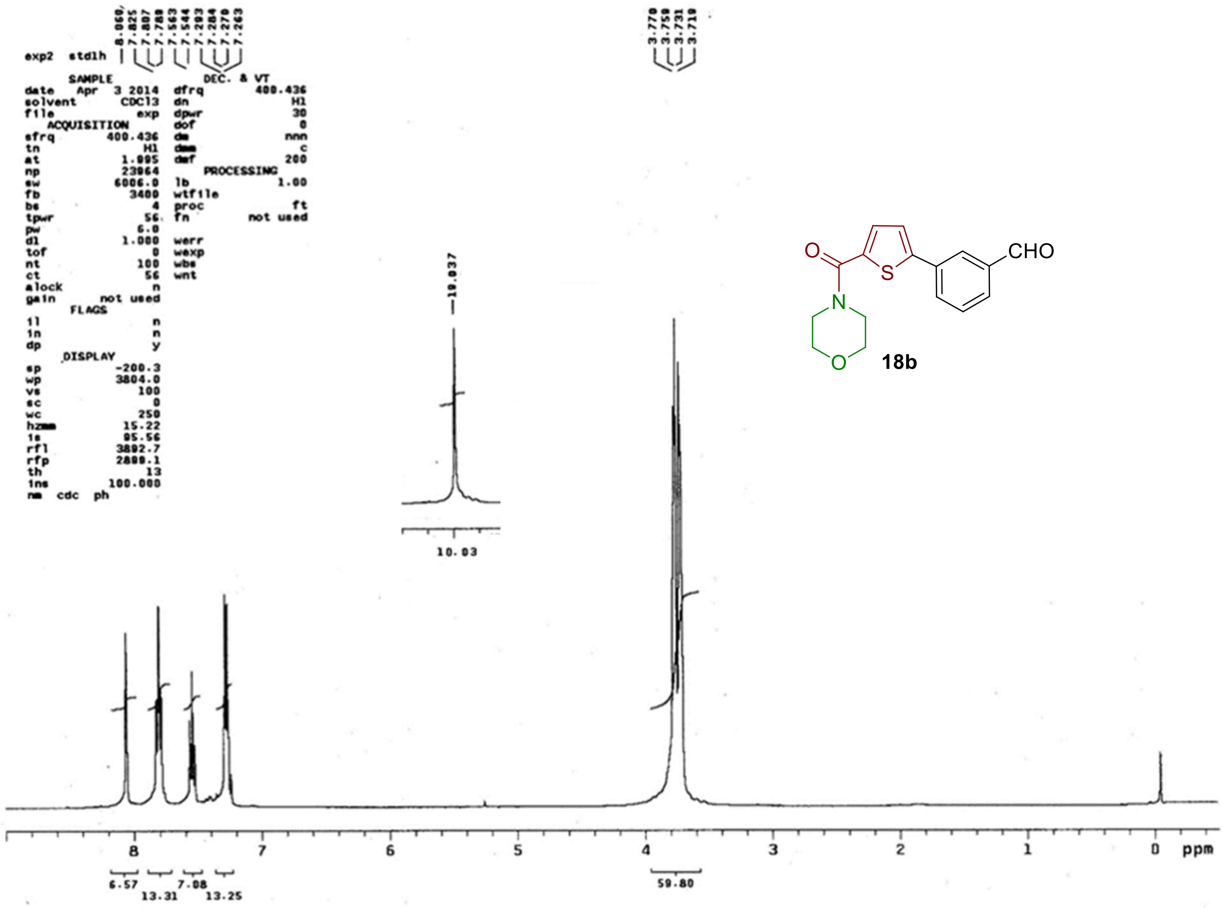

^{1}H NMR spectrum of compound **18b**

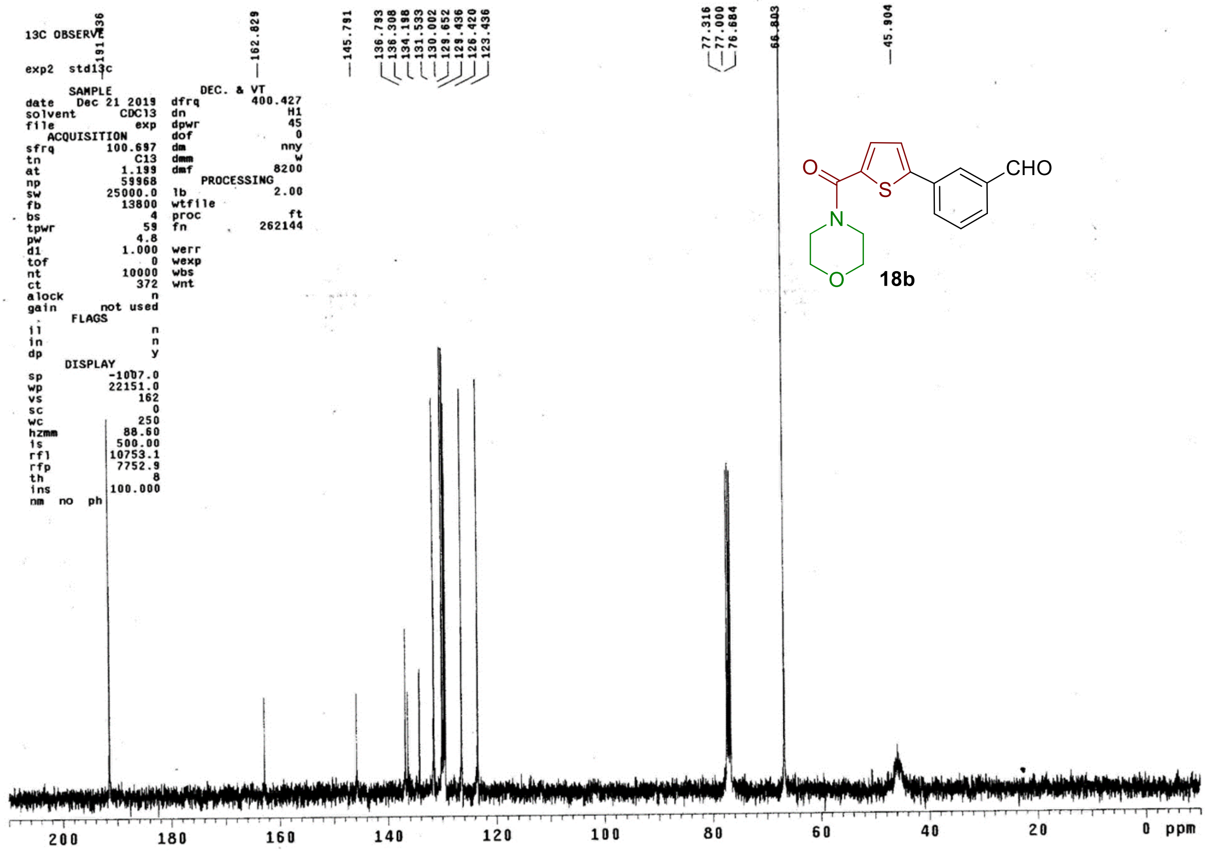

^{13}C NMR spectrum of compound **18b**

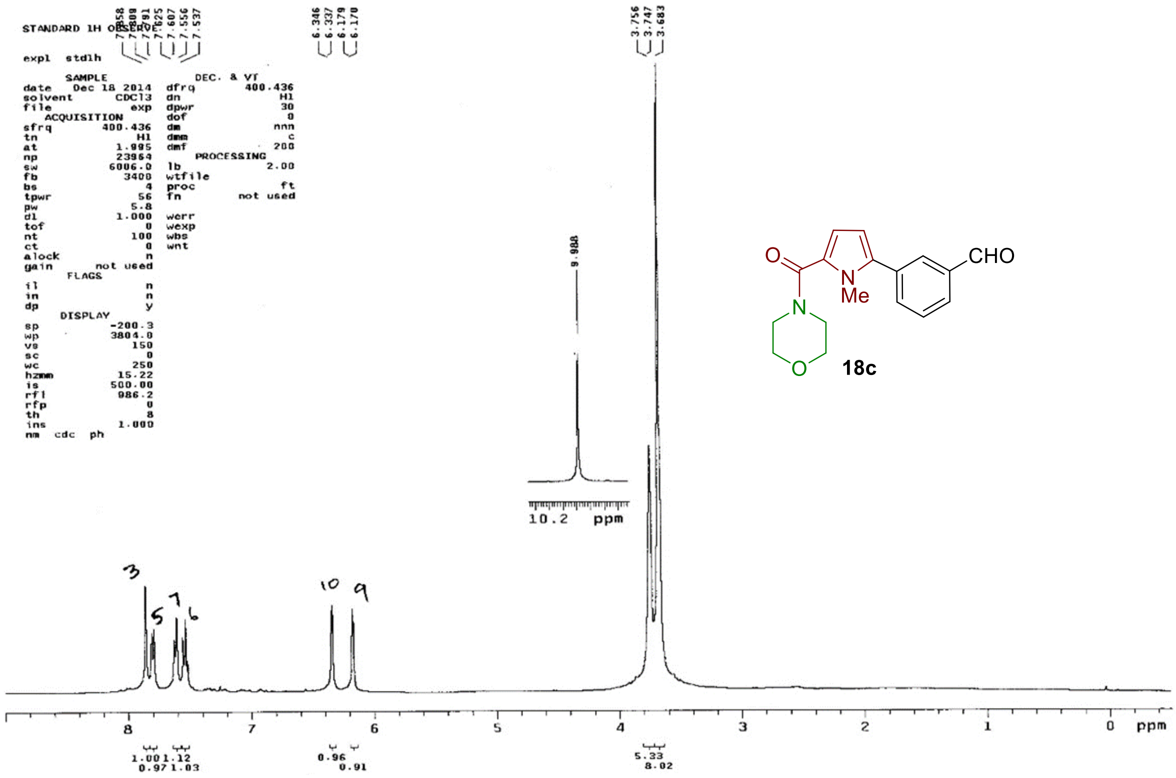

^{1}H NMR spectrum of compound **18c**

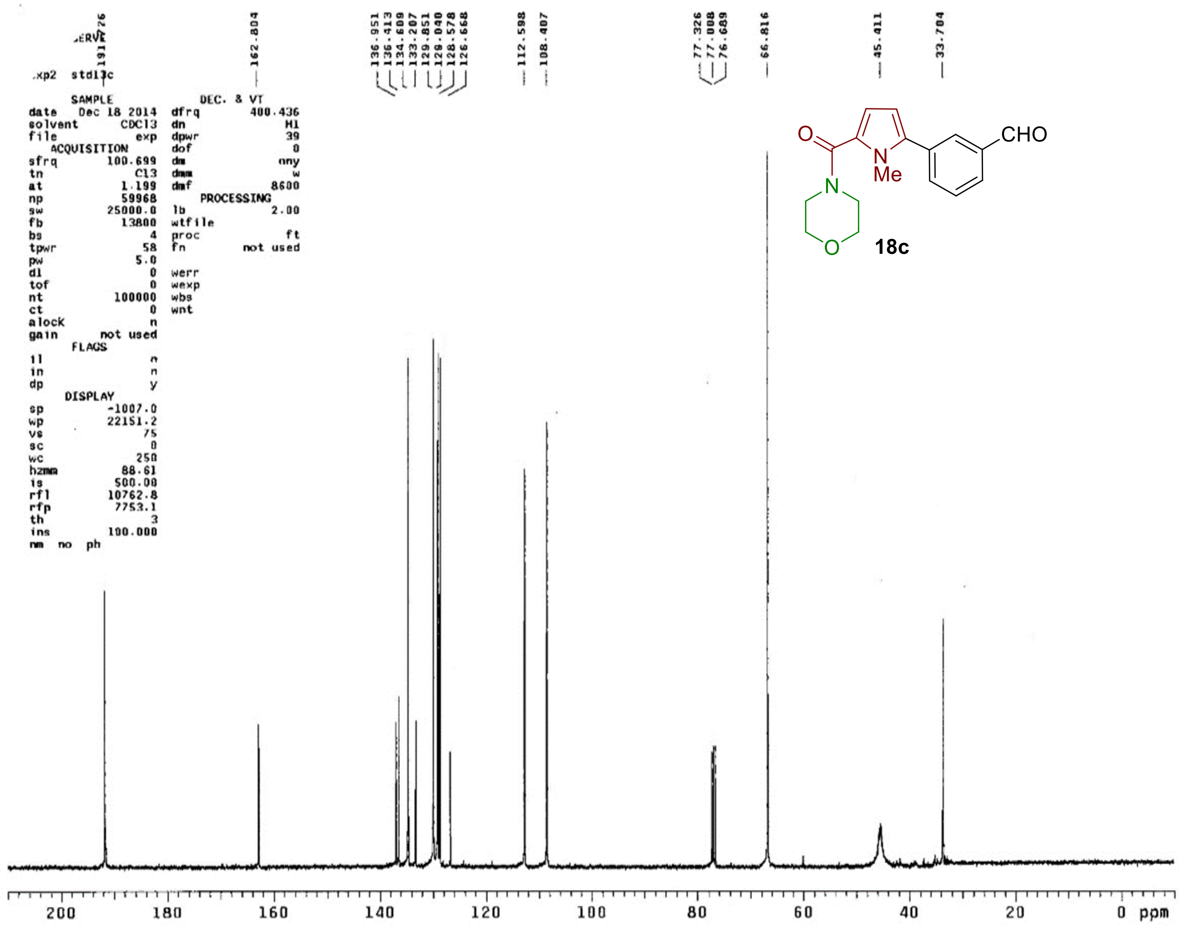

^{13}C NMR spectrum of compound **18c**

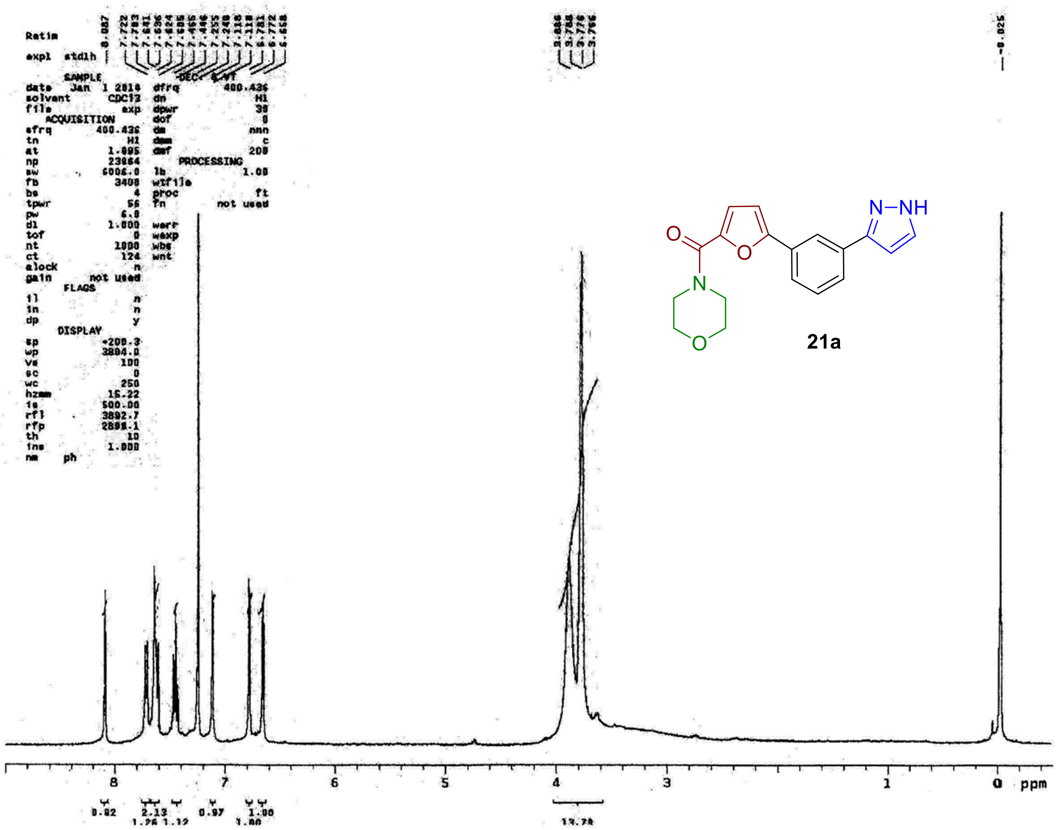

^{1}H NMR spectrum of compound **21a**

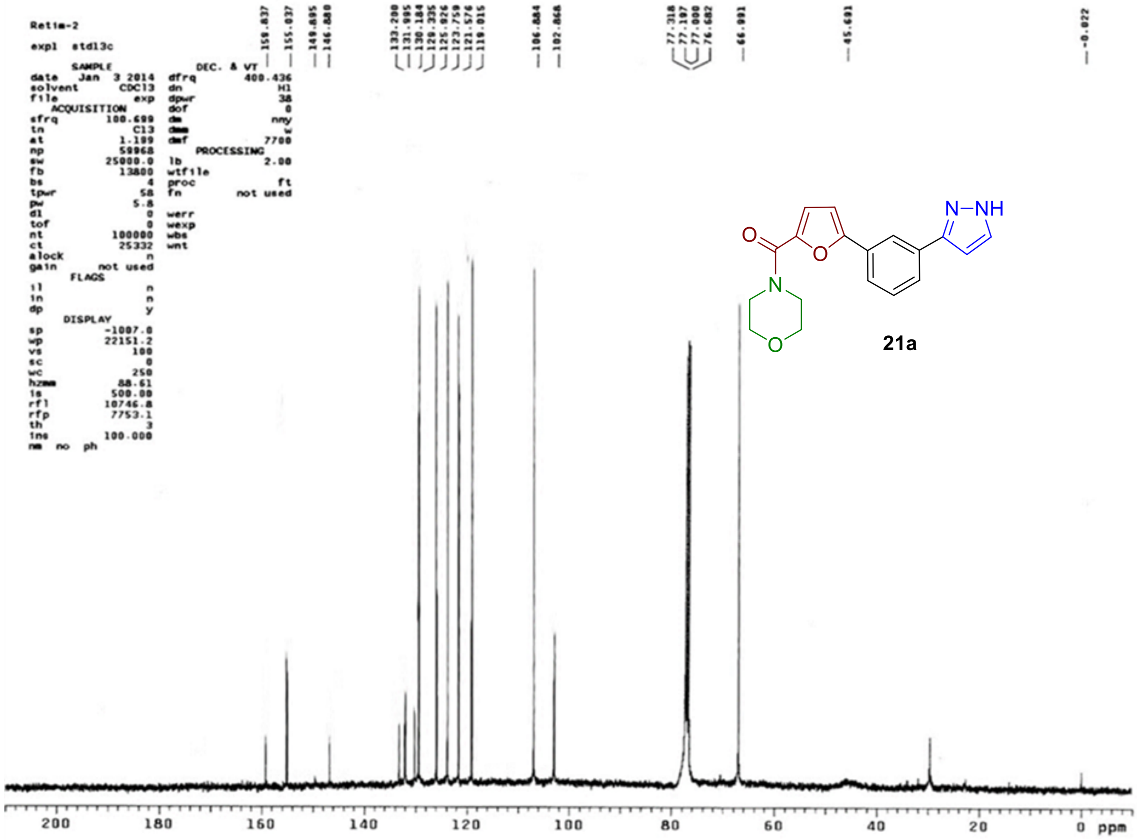

^{13}C NMR spectrum of compound **21a**

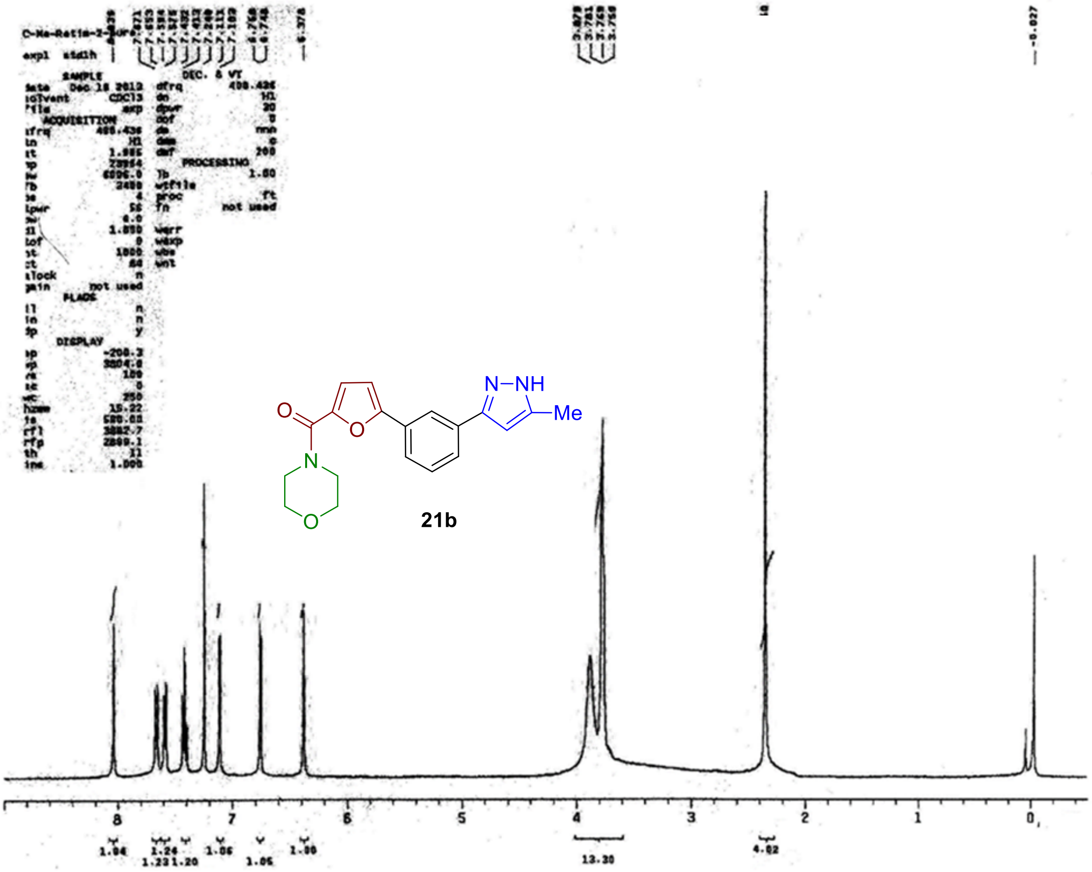

^{1}H NMR spectrum of compound **21b**

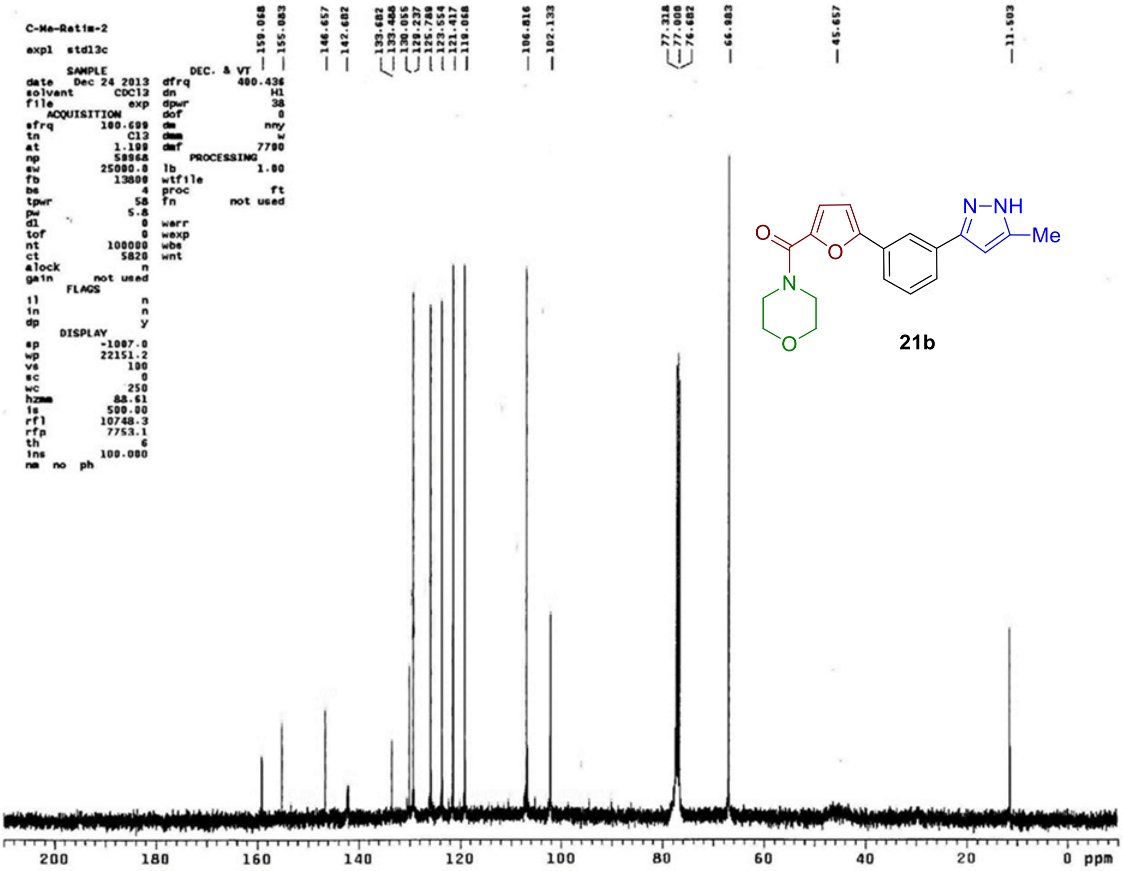

^{13}C NMR spectrum of compound **21b**

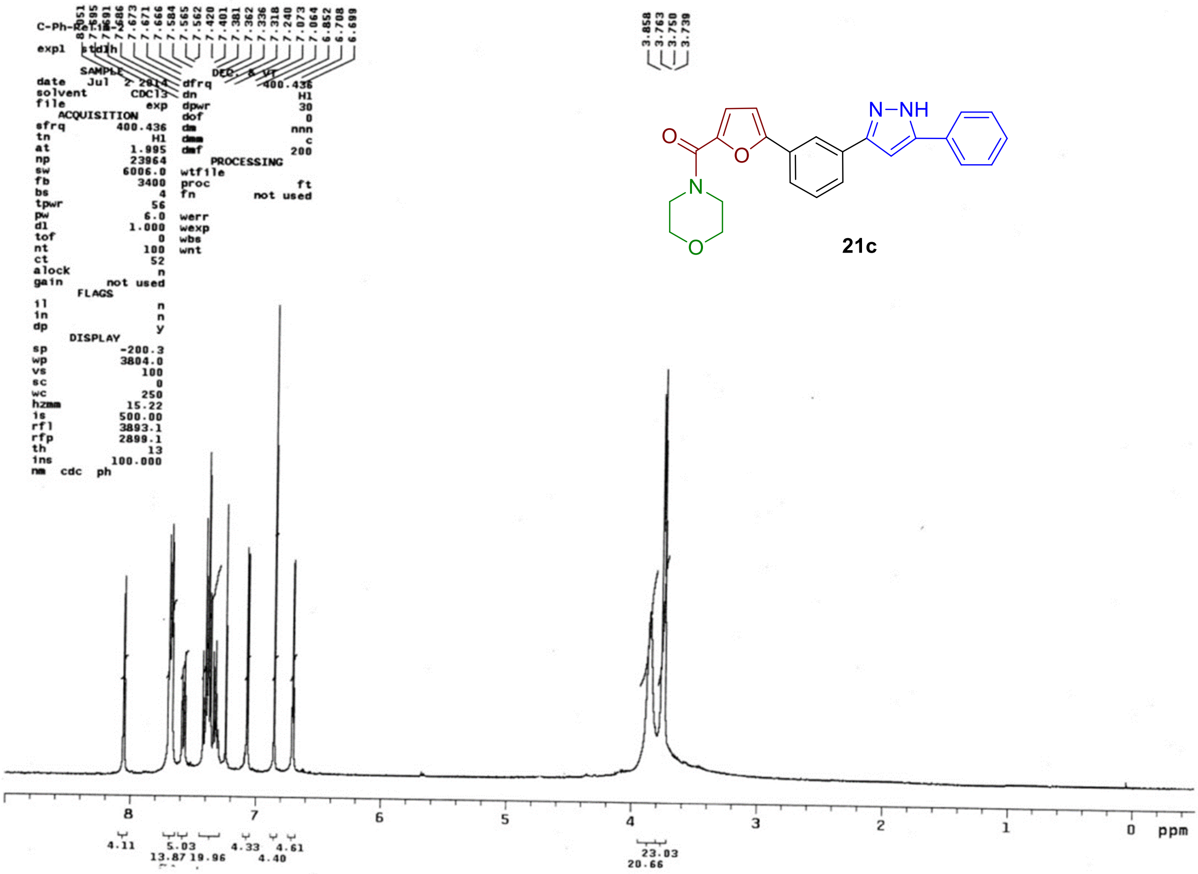

^{1}H NMR spectrum of compound **21c**

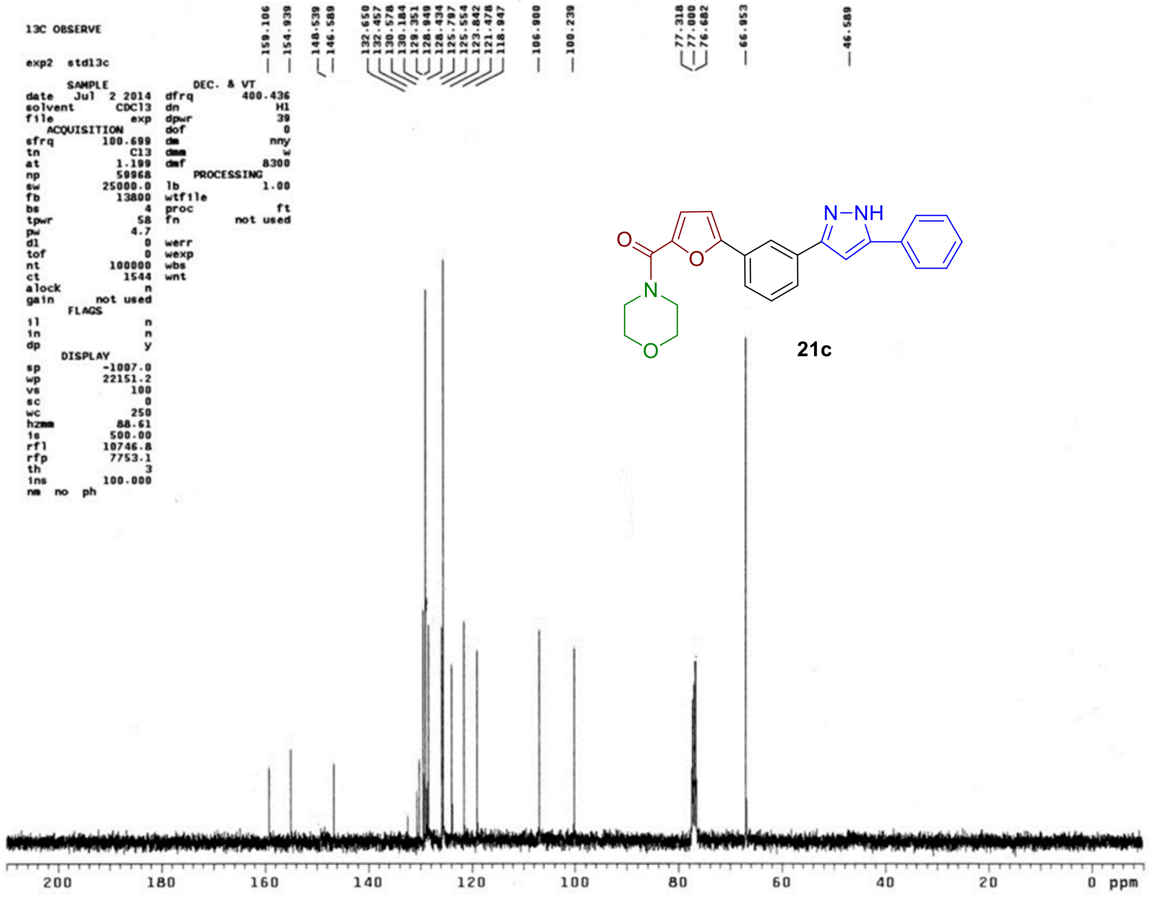

^{13}C NMR spectrum of compound **21c**

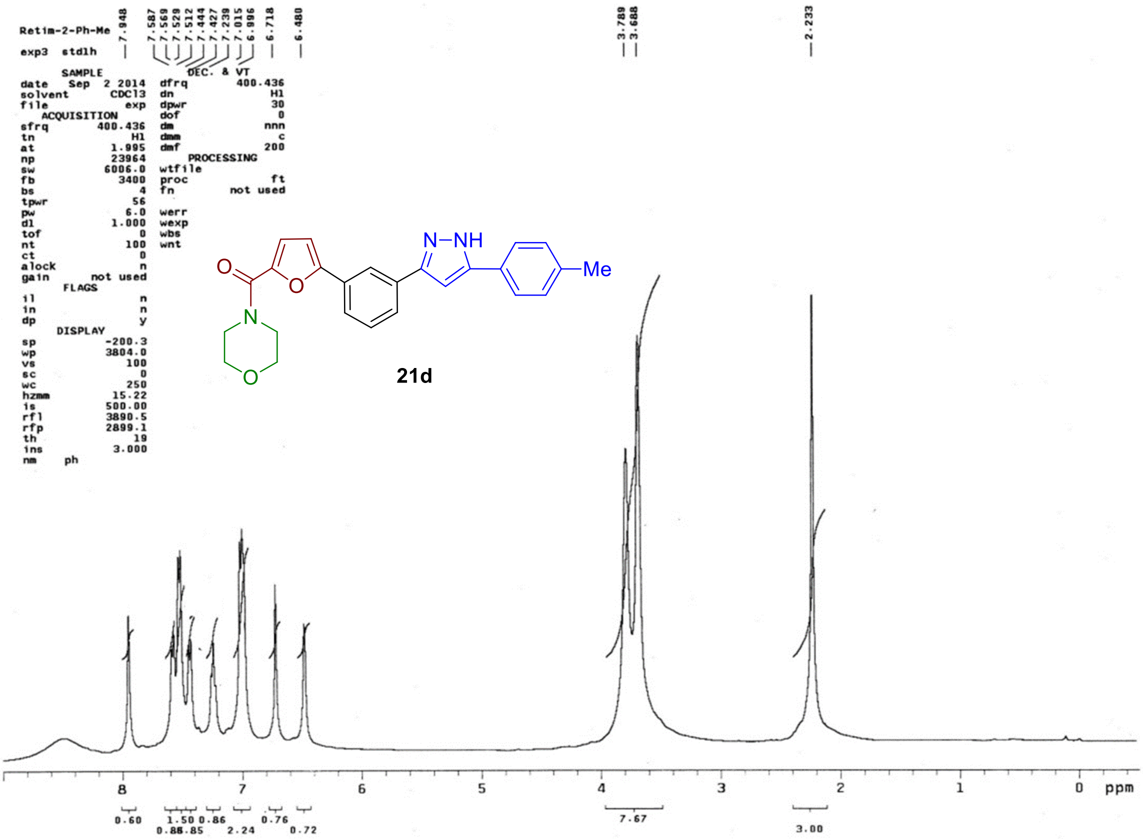

^{1}H NMR spectrum of compound **21d**

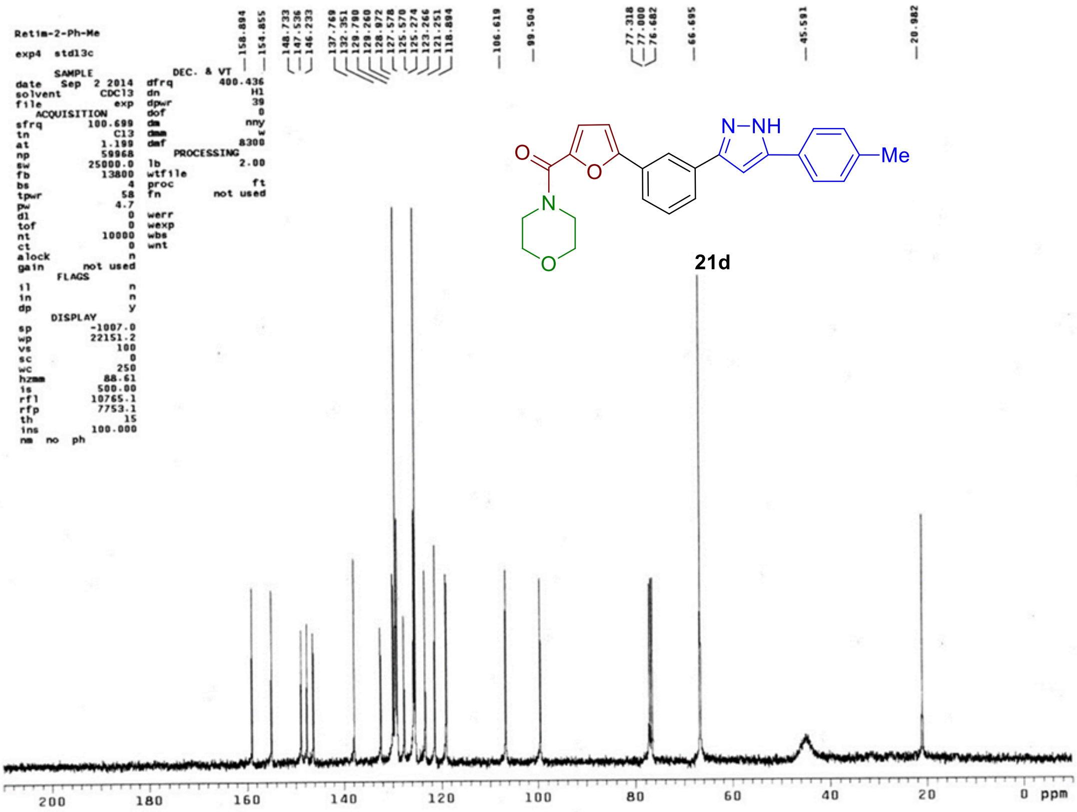

^{13}C NMR spectrum of compound **21d**

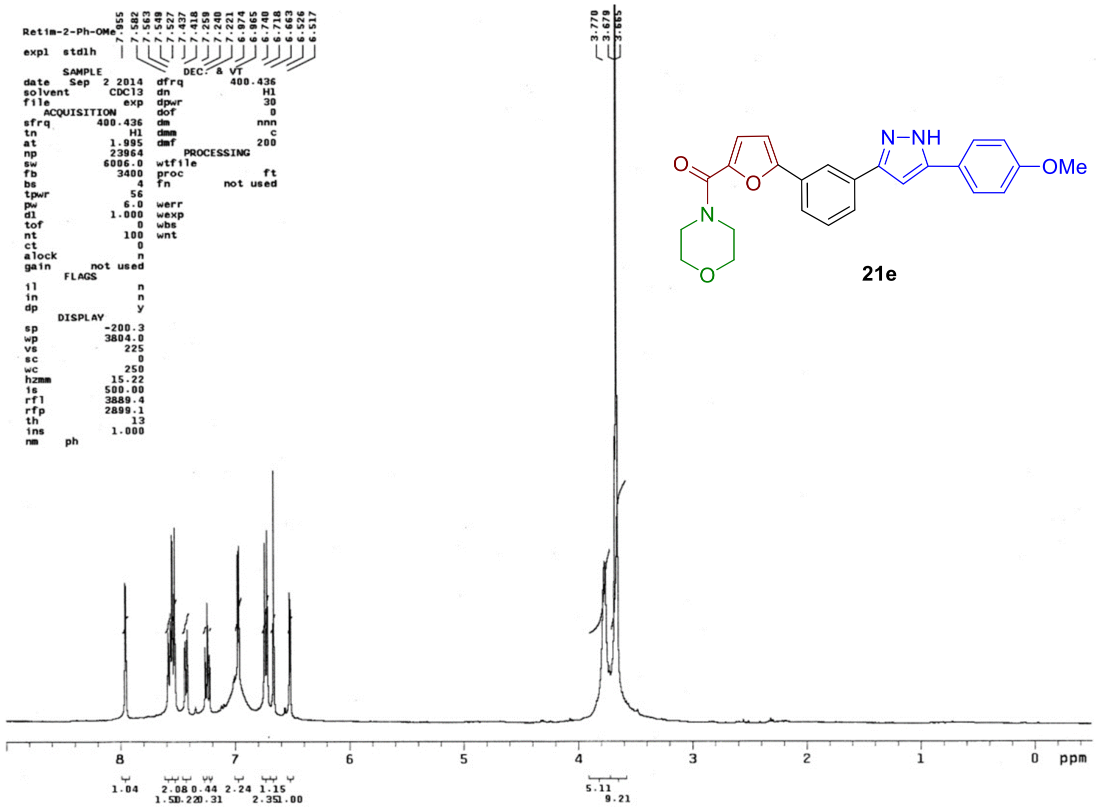

^{1}H NMR spectrum of compound **21e**

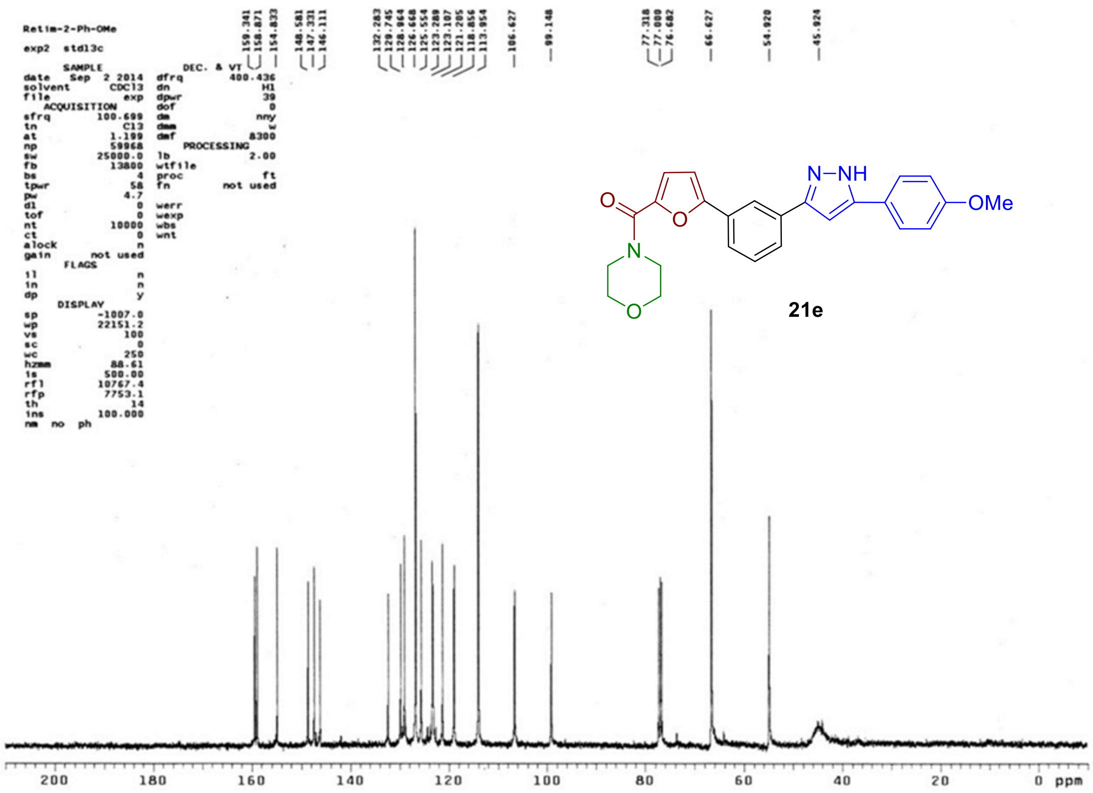

^{13}C NMR spectrum of compound **21e**

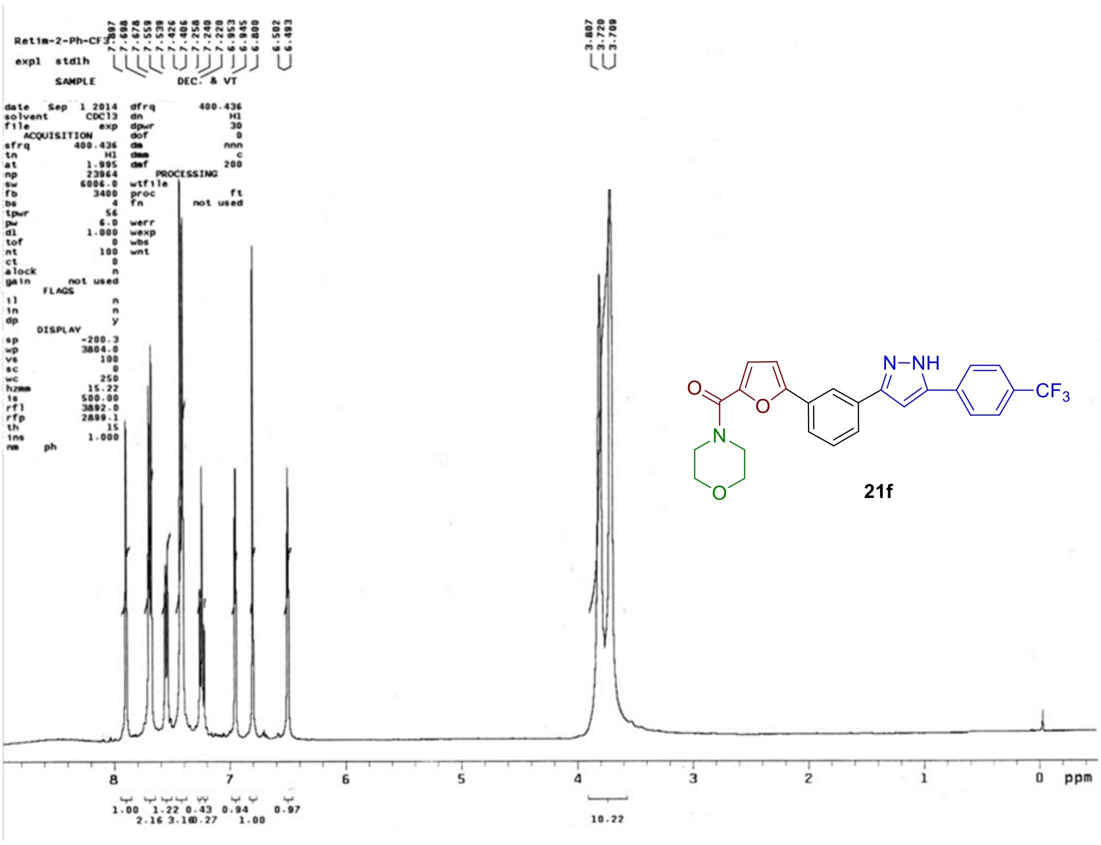

^{1}H NMR spectrum of compound **21f**

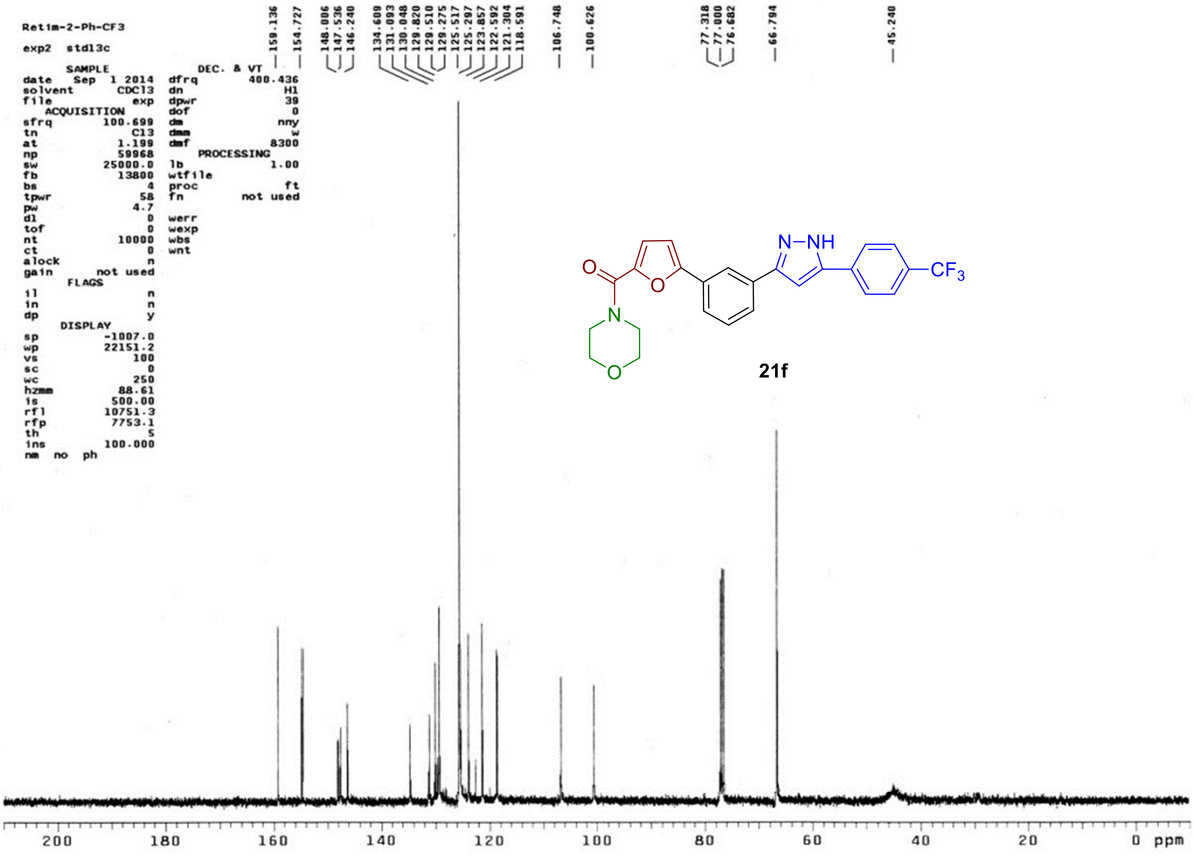

^{13}C NMR spectrum of compound **21f**

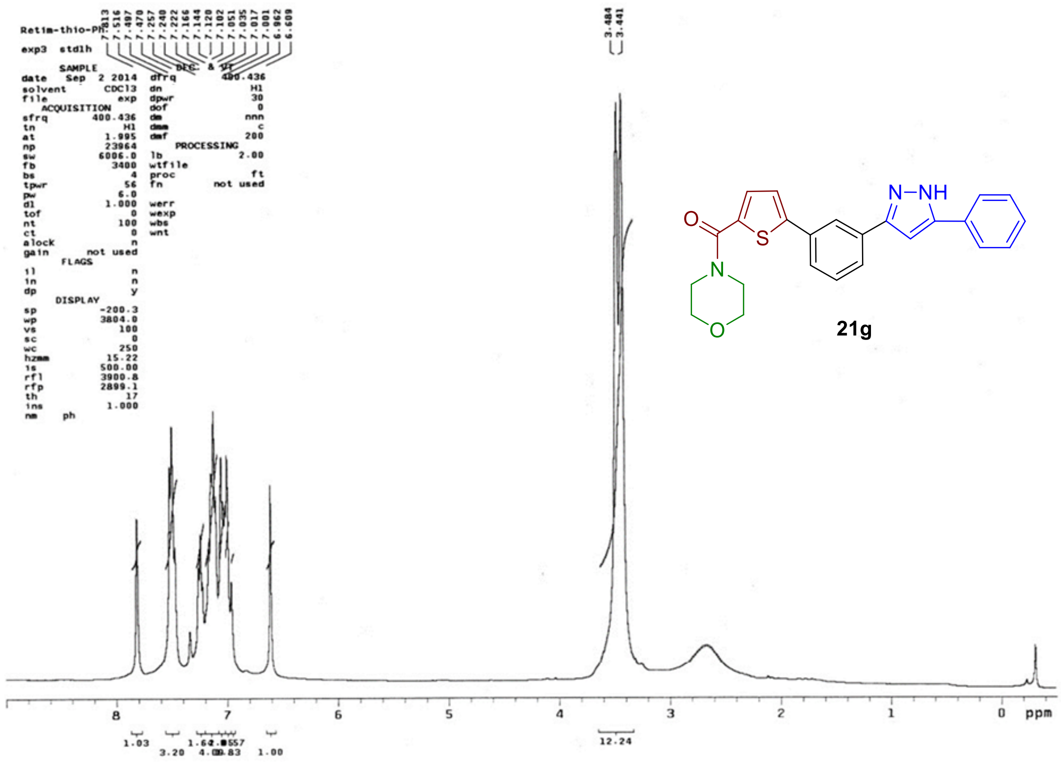

^{1}H NMR spectrum of compound **21g**

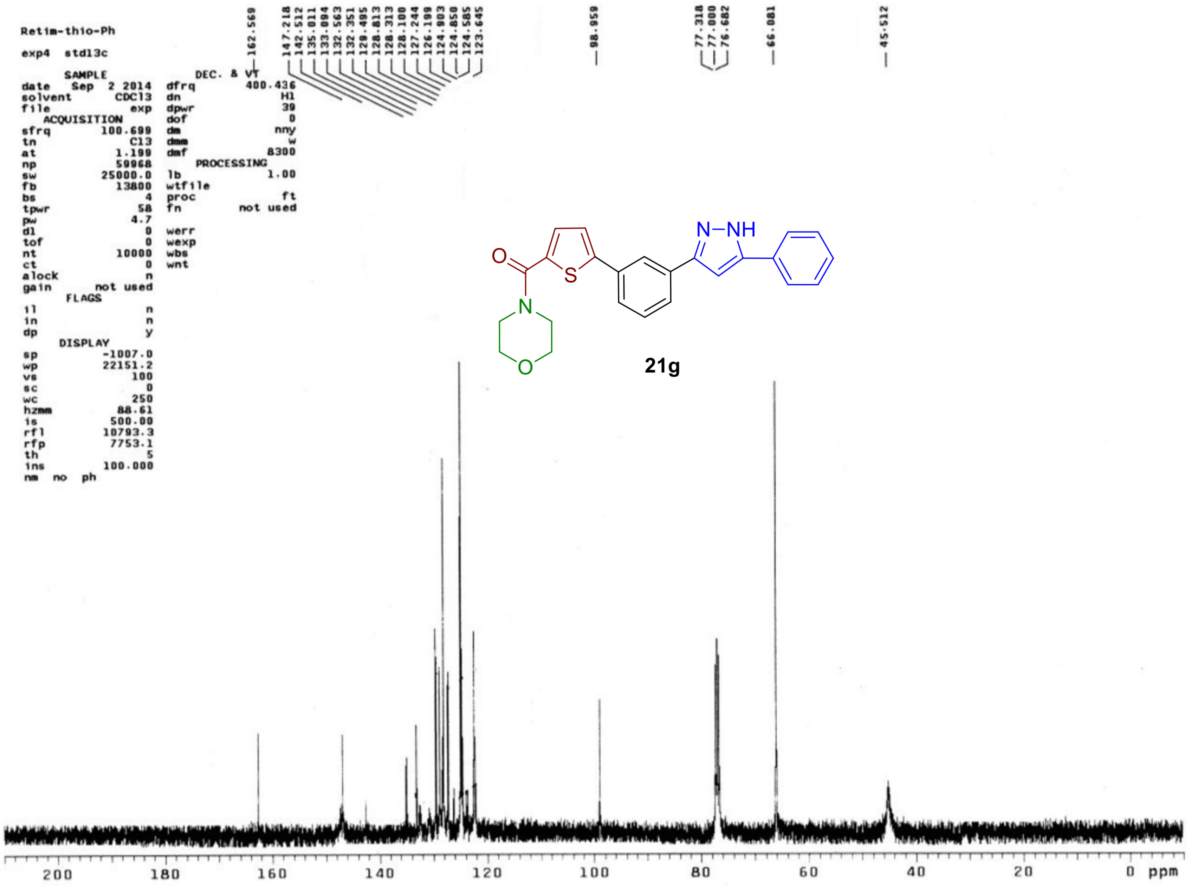

^{13}C NMR spectrum of compound **21g**

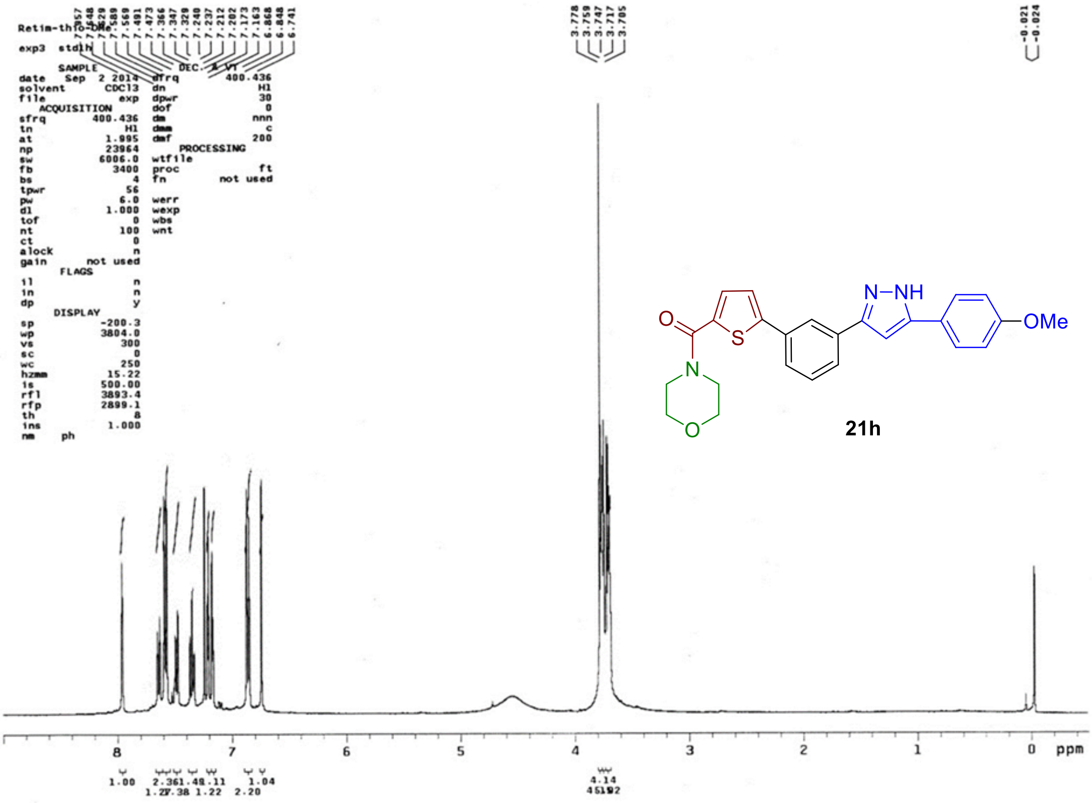

^{1}H NMR spectrum of compound **21h**

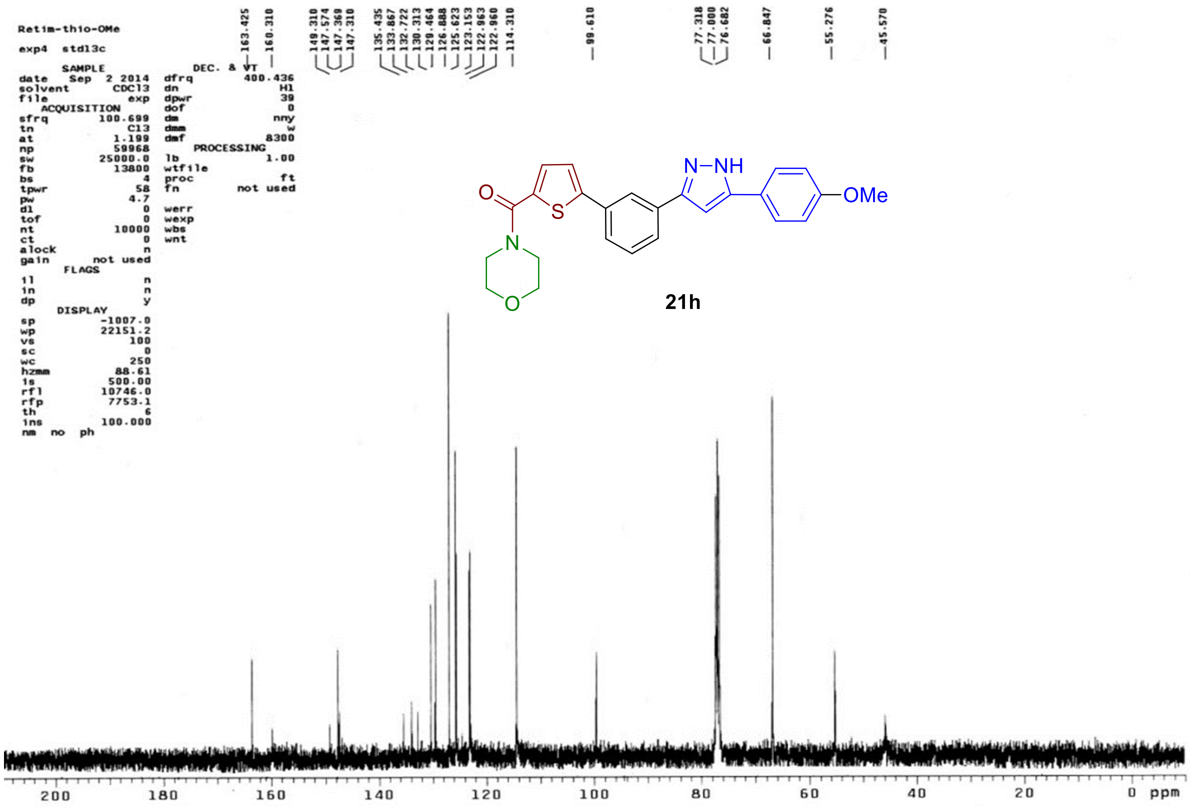

^{13}C NMR spectrum of compound **21h**

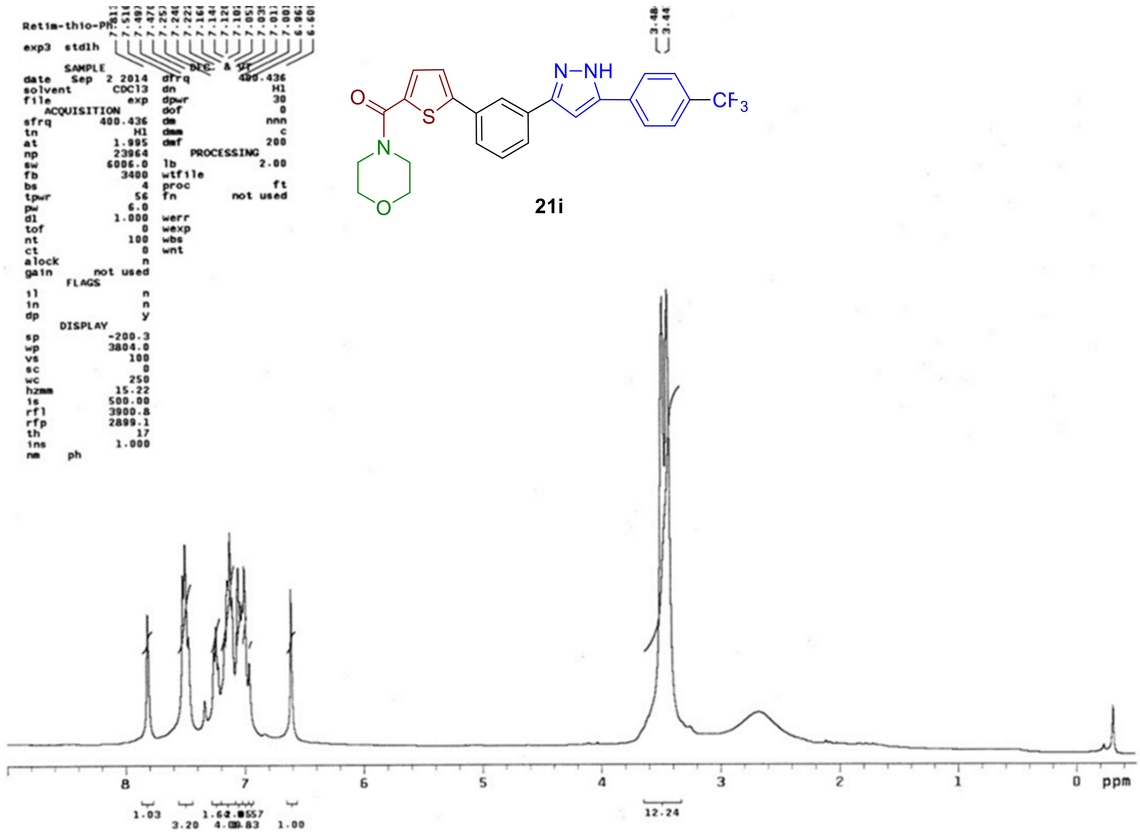

^{1}H NMR spectrum of compound **21i**

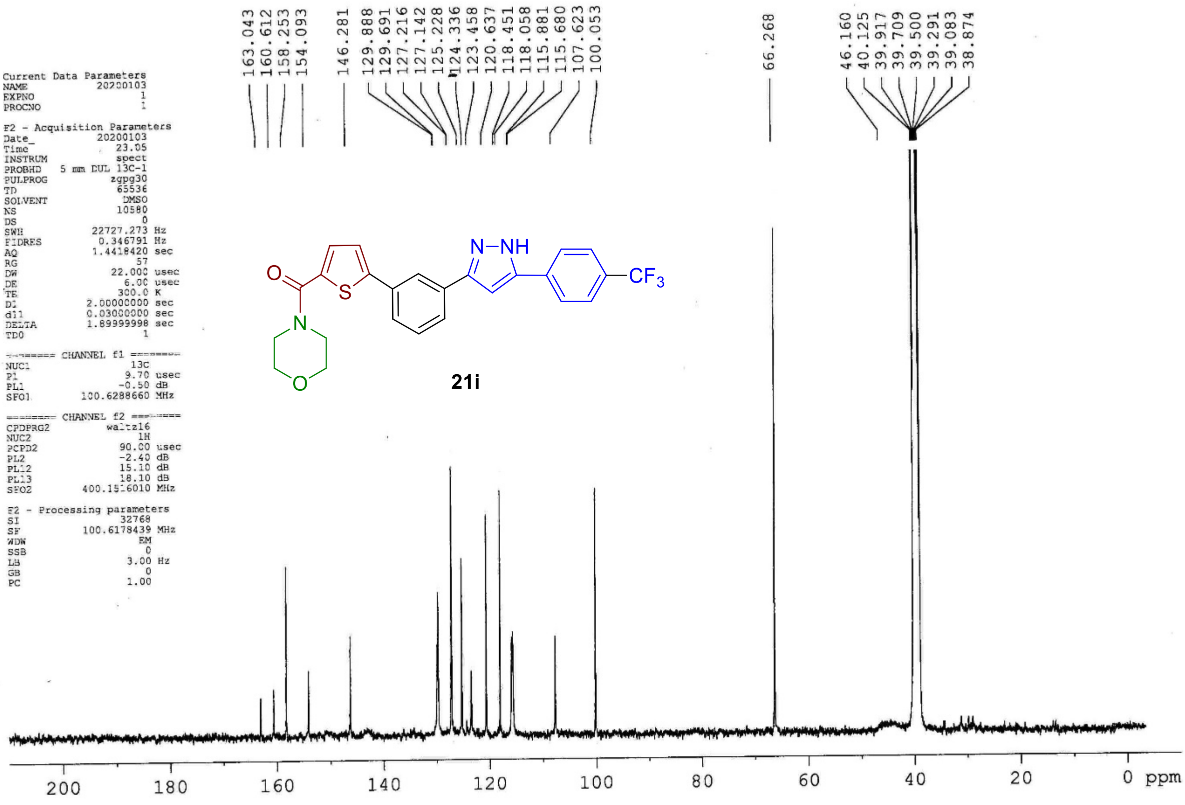

^{13}C NMR spectrum of compound **21i**

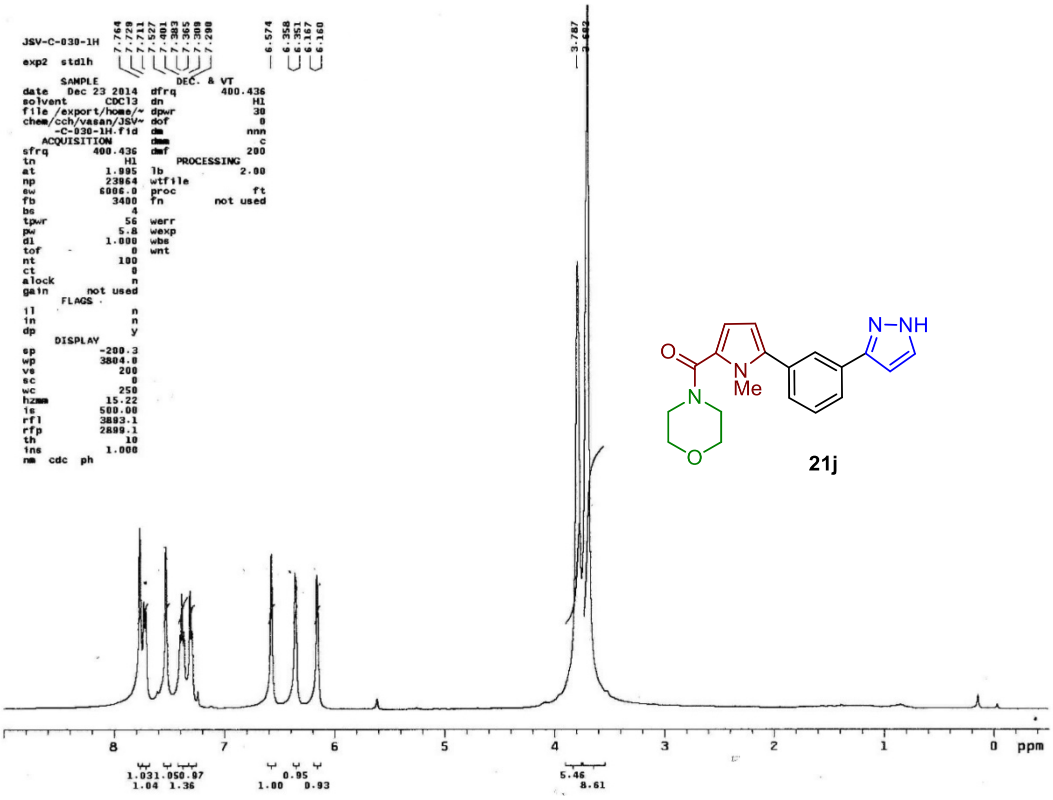

^{1}H NMR spectrum of compound **21j**

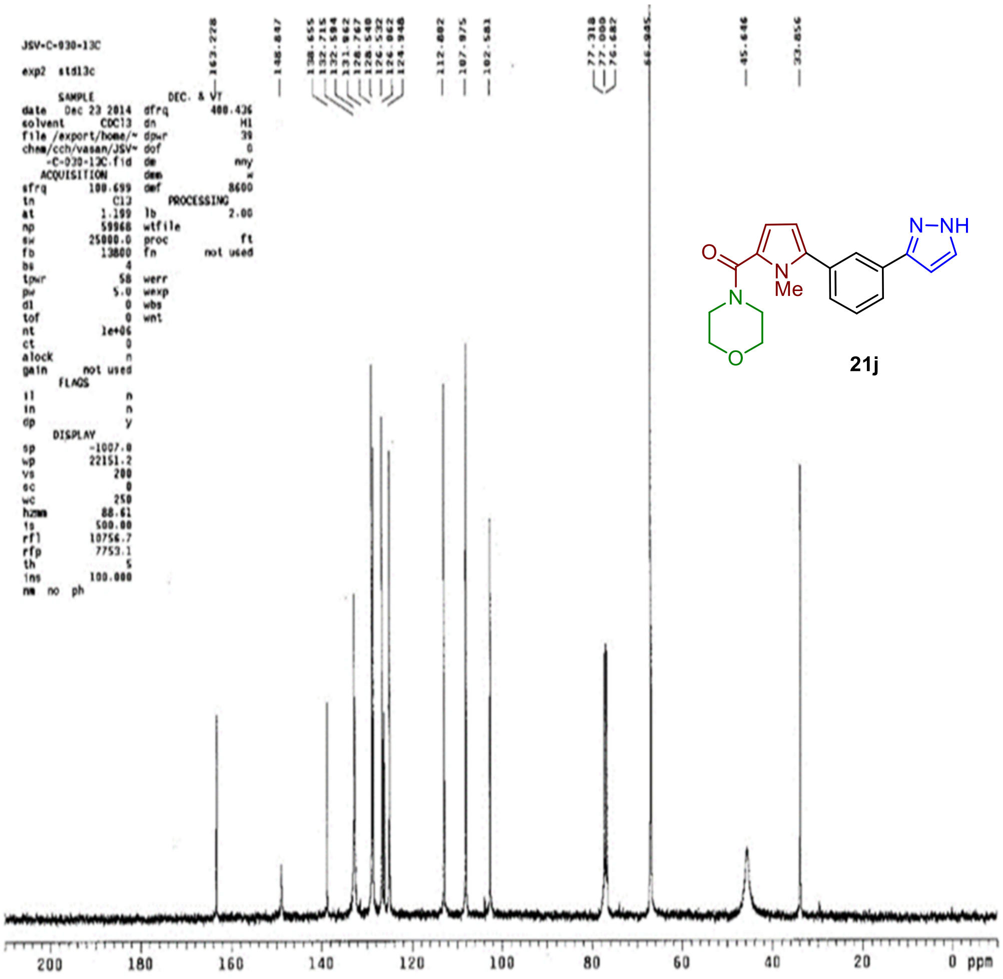

^{13}C NMR spectrum of compound **21j**

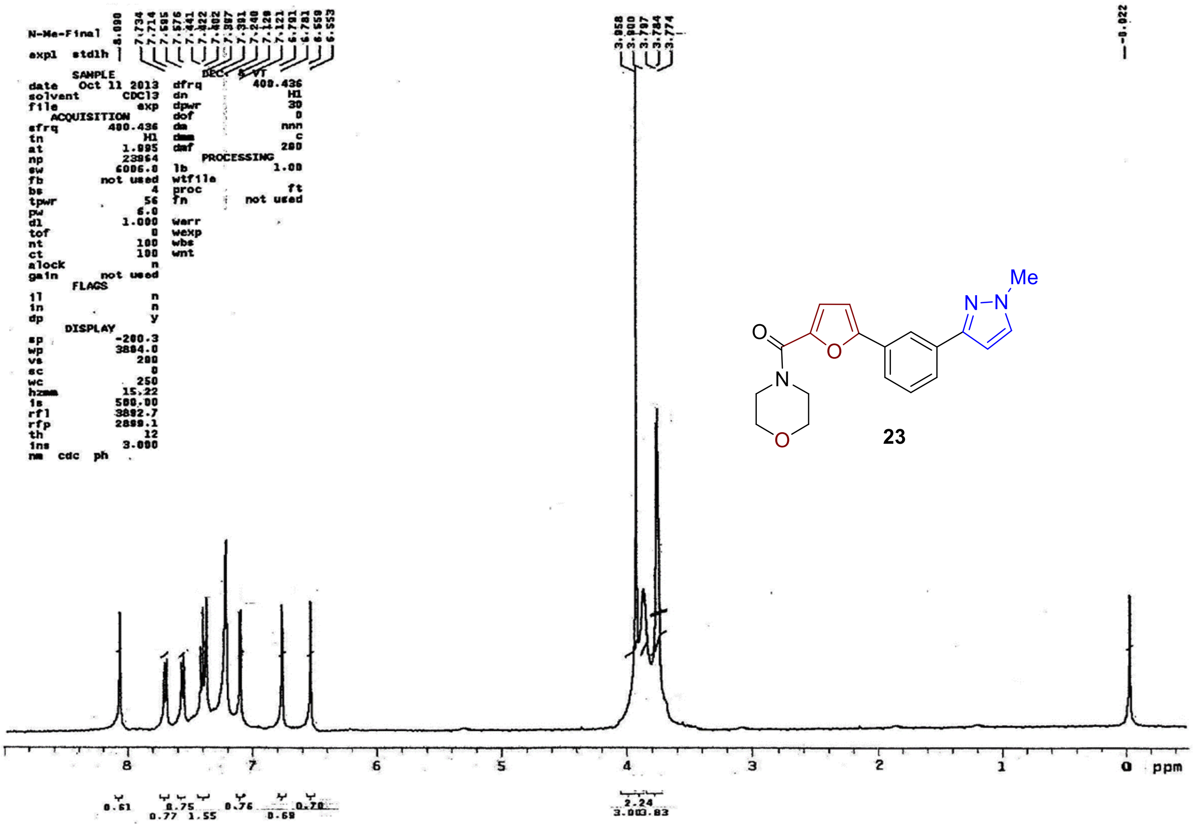

^{1}H NMR spectrum of compound **23**

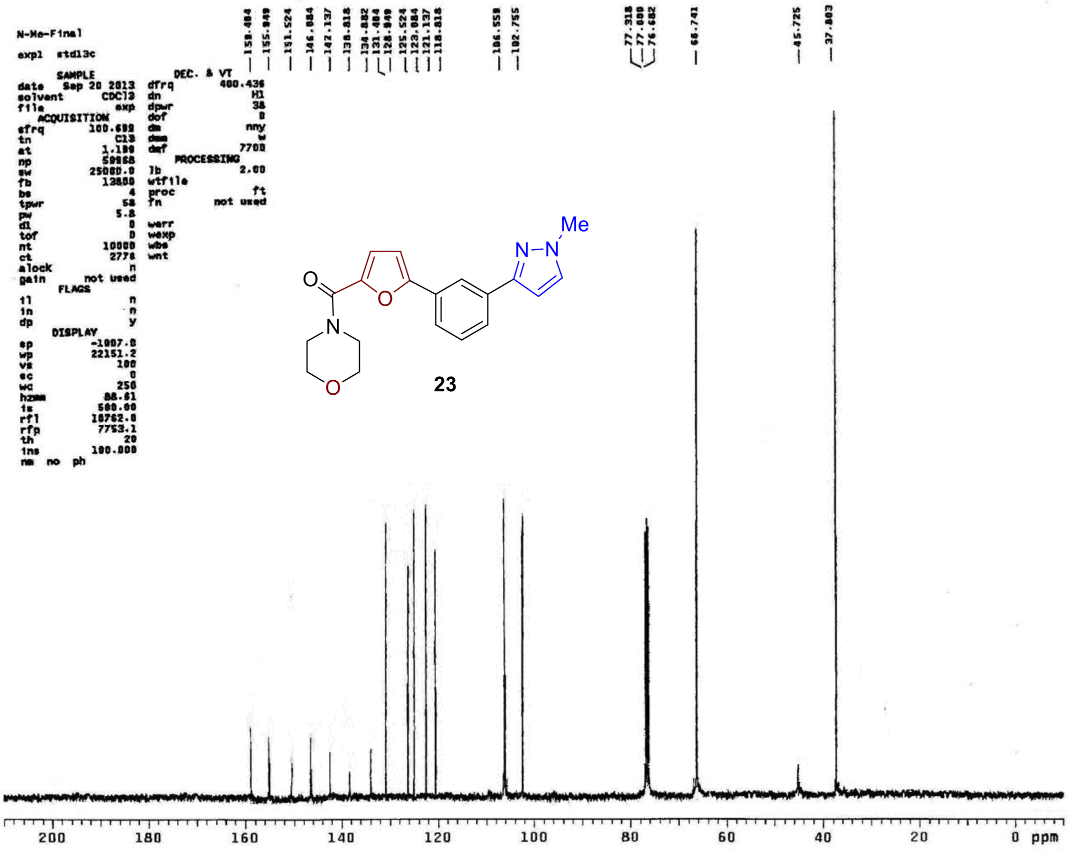

^{13}C NMR spectrum of compound **23**

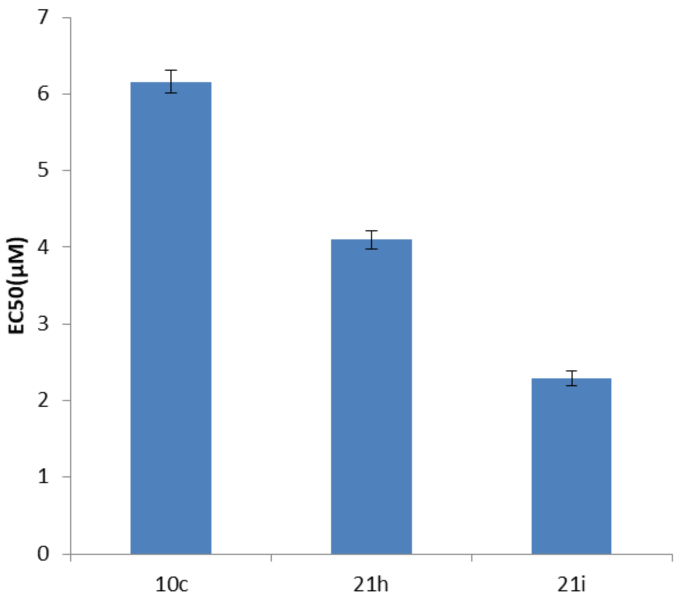

Figure S1. Comparison of EC_{50} values among compounds **10c**, **21h**, and **21i**.

X-ray Crystal Data of Compound 21g

Table 1. Crystal data and structure refinement for mo_150629_0m_a.

Identification code	mo_150629_0m_a	
Empirical formula	C24 H21 N3 O2 S	
Formula weight	415.50	
Temperature	296(2) K	
Wavelength	0.71073 Å	
Crystal system	Monoclinic	
Space group	C 2/c	
Unit cell dimensions	a = 27.0531(13) Å	α= 90°.
	b = 6.0926(3) Å	β= 109.8440(10)°.
	c = 26.4787(13) Å	γ = 90°.
Volume	4105.2(3) Å^3	
Z	8	
Density (calculated)	1.345 Mg/m^3	
Absorption coefficient	0.184 mm^{-1}	
F(000)	1744	
Crystal size	0.20 x 0.15 x 0.15 mm^3	
Theta range for data collection	1.600 to 26.616°.	
Index ranges	-33<=h<=33, -7<=k<=5, -33<=l<=33	
Reflections collected	17304	
Independent reflections	4292 [R(int) = 0.0438]	
Completeness to theta = 25.242°	99.9 %	
Absorption correction	Semi-empirical from equivalents	
Max. and min. transmission	0.9485 and 0.8934	
Refinement method	Full-matrix least-squares on F^2	

Data / restraints / parameters	4292 / 216 / 326
Goodness-of-fit on F^2	1.020
Final R indices [I>2sigma(I)]	R1 = 0.0515, wR2 = 0.1296
R indices (all data)	R1 = 0.0906, wR2 = 0.1531
Extinction coefficient	n/a
Largest diff. peak and hole	0.448 and -0.459 e.Å^{-3}

Table 2. Atomic coordinates (x 10^4) and equivalent isotropic displacement parameters ($Å^2$x 10^3) for mo_150629_0m_a. U(eq) is defined as one third of the trace of the orthogonalized U^{ij} tensor.

	x	y	z	U(eq)
C(1)	6104(1)	1523(4)	8963(1)	51(1)
C(2)	6216(1)	-521(5)	9197(1)	68(1)
C(3)	6733(1)	-1209(6)	9429(1)	80(1)
C(4)	7140(1)	120(6)	9426(1)	80(1)
C(5)	7032(1)	2146(6)	9188(1)	77(1)
C(6)	6520(1)	2831(5)	8957(1)	63(1)
C(7)	5558(1)	2310(4)	8724(1)	50(1)
C(8)	5362(1)	4098(4)	8384(1)	53(1)
C(9)	4825(1)	4076(4)	8270(1)	49(1)
C(10)	4403(1)	5436(4)	7906(1)	48(1)
C(11)	4506(1)	7534(4)	7764(1)	55(1)
C(12)	4117(1)	8708(4)	7381(1)	59(1)
C(13)	1456(1)	4326(5)	6288(1)	56(1)
C(14)	3898(1)	4588(4)	7677(1)	50(1)
C(15)	3501(1)	5765(4)	7297(1)	47(1)
C(16)	3623(1)	7834(4)	7145(1)	56(1)
C(17)	2976(1)	4781(4)	7069(1)	48(1)
C(18)	2817(1)	2737(4)	7165(1)	53(1)
C(19)	2280(1)	2311(4)	6884(1)	54(1)
C(20)	2030(1)	4041(4)	6580(1)	51(1)
N(1)	5168(1)	1254(4)	8816(1)	60(1)

N(2)	4725(1)	2343(4)	8537(1)	57(1)
O(1)	1242(1)	5993(3)	6378(1)	73(1)
S(1)	2451(1)	6209(1)	6635(1)	56(1)
C(21)	1425(1)	731(7)	5823(2)	66(1)
C(22)	1230(2)	536(10)	5224(2)	96(1)
C(23)	617(1)	2742(8)	5727(1)	69(1)
C(24)	463(2)	2477(9)	5134(2)	88(1)
N(3)	1192(1)	2671(5)	5978(1)	55(1)
O(2)	673(1)	542(7)	4995(1)	107(1)
C(21')	1486(6)	1810(30)	5514(8)	73(2)
C(22')	1207(6)	-300(30)	5510(9)	85(3)
C(23')	681(5)	3780(30)	5443(8)	75(3)
C(24')	417(7)	1610(30)	5413(10)	88(3)
N(3')	1251(5)	3500(30)	5766(6)	66(2)
O(2')	659(6)	-170(30)	5254(6)	91(3)

Table 3. Bond lengths [Å] and angles [°] for mo_150629_0m_a.

C(1)-C(2)	1.378(4)
C(1)-C(6)	1.385(4)
C(1)-C(7)	1.474(3)
C(2)-C(3)	1.389(4)
C(2)-H(2)	0.9500
C(3)-C(4)	1.369(4)
C(3)-H(3)	0.9500
C(4)-C(5)	1.372(5)
C(4)-H(4)	0.9500
C(5)-C(6)	1.376(4)
C(5)-H(5)	0.9500
C(6)-H(6)	0.9500
C(7)-N(1)	1.327(3)
C(7)-C(8)	1.398(3)
C(8)-C(9)	1.380(3)
C(8)-H(8)	0.9500
C(9)-N(2)	1.348(3)
C(9)-C(10)	1.472(3)
C(10)-C(11)	1.386(4)
C(10)-C(14)	1.392(3)
C(11)-C(12)	1.386(4)
C(11)-H(11)	0.9500
C(12)-C(16)	1.376(4)
C(12)-H(12)	0.9500

C(13)-O(1)	1.232(3)
C(13)-N(3)	1.342(4)
C(13)-C(20)	1.492(3)
C(14)-C(15)	1.396(3)
C(14)-H(14)	0.9500
C(15)-C(16)	1.396(4)
C(15)-C(17)	1.470(3)
C(16)-H(16)	0.9500
C(17)-C(18)	1.369(4)
C(17)-S(1)	1.727(2)
C(18)-C(19)	1.412(3)
C(18)-H(18)	0.9500
C(19)-C(20)	1.359(4)
C(19)-H(19)	0.9500
C(20)-S(1)	1.717(3)
N(1)-N(2)	1.349(3)
N(1)-H(1)	0.8800
C(21)-N(3)	1.462(4)
C(21)-C(22)	1.496(5)
C(21)-H(21A)	0.9900
C(21)-H(21B)	0.9900
C(22)-O(2)	1.420(5)
C(22)-H(22A)	0.9900
C(22)-H(22B)	0.9900
C(23)-N(3)	1.471(4)
C(23)-C(24)	1.491(5)

C(23)-H(23A)	0.9900
C(23)-H(23B)	0.9900
C(24)-O(2)	1.410(5)
C(24)-H(24A)	0.9900
C(24)-H(24B)	0.9900
C(21')-N(3')	1.482(14)
C(21')-C(22')	1.493(16)
C(21')-H(21C)	0.9900
C(21')-H(21D)	0.9900
C(22')-O(2')	1.406(15)
C(22')-H(22C)	0.9900
C(22')-H(22D)	0.9900
C(23')-C(24')	1.491(16)
C(23')-N(3')	1.496(14)
C(23')-H(23C)	0.9900
C(23')-H(23D)	0.9900
C(24')-O(2')	1.405(15)
C(24')-H(24C)	0.9900
C(24')-H(24D)	0.9900

C(2)-C(1)-C(6)	117.9(2)
C(2)-C(1)-C(7)	121.5(2)
C(6)-C(1)-C(7)	120.5(2)
C(1)-C(2)-C(3)	120.5(3)
C(1)-C(2)-H(2)	119.8
C(3)-C(2)-H(2)	119.8

C(4)-C(3)-C(2)	120.8(3)
C(4)-C(3)-H(3)	119.6
C(2)-C(3)-H(3)	119.6
C(3)-C(4)-C(5)	119.2(3)
C(3)-C(4)-H(4)	120.4
C(5)-C(4)-H(4)	120.4
C(4)-C(5)-C(6)	120.2(3)
C(4)-C(5)-H(5)	119.9
C(6)-C(5)-H(5)	119.9
C(5)-C(6)-C(1)	121.4(3)
C(5)-C(6)-H(6)	119.3
C(1)-C(6)-H(6)	119.3
N(1)-C(7)-C(8)	110.2(2)
N(1)-C(7)-C(1)	120.4(2)
C(8)-C(7)-C(1)	129.5(2)
C(9)-C(8)-C(7)	106.0(2)
C(9)-C(8)-H(8)	127.0
C(7)-C(8)-H(8)	127.0
N(2)-C(9)-C(8)	106.0(2)
N(2)-C(9)-C(10)	122.4(2)
C(8)-C(9)-C(10)	131.4(2)
C(11)-C(10)-C(14)	118.7(2)
C(11)-C(10)-C(9)	121.0(2)
C(14)-C(10)-C(9)	120.2(2)
C(12)-C(11)-C(10)	120.2(2)
C(12)-C(11)-H(11)	119.9

C(10)-C(11)-H(11)	119.9
C(16)-C(12)-C(11)	120.7(3)
C(16)-C(12)-H(12)	119.6
C(11)-C(12)-H(12)	119.6
O(1)-C(13)-N(3)	123.4(2)
O(1)-C(13)-C(20)	118.1(2)
N(3)-C(13)-C(20)	118.2(2)
C(10)-C(14)-C(15)	121.7(2)
C(10)-C(14)-H(14)	119.1
C(15)-C(14)-H(14)	119.1
C(14)-C(15)-C(16)	118.1(2)
C(14)-C(15)-C(17)	119.2(2)
C(16)-C(15)-C(17)	122.7(2)
C(12)-C(16)-C(15)	120.5(2)
C(12)-C(16)-H(16)	119.8
C(15)-C(16)-H(16)	119.8
C(18)-C(17)-C(15)	128.0(2)
C(18)-C(17)-S(1)	110.04(18)
C(15)-C(17)-S(1)	121.97(19)
C(17)-C(18)-C(19)	113.6(2)
C(17)-C(18)-H(18)	123.2
C(19)-C(18)-H(18)	123.2
C(20)-C(19)-C(18)	112.7(2)
C(20)-C(19)-H(19)	123.7
C(18)-C(19)-H(19)	123.7
C(19)-C(20)-C(13)	128.3(3)

C(19)-C(20)-S(1)	111.31(19)
C(13)-C(20)-S(1)	119.88(19)
C(7)-N(1)-N(2)	105.9(2)
C(7)-N(1)-H(1)	127.0
N(2)-N(1)-H(1)	127.0
C(9)-N(2)-N(1)	112.0(2)
C(20)-S(1)-C(17)	92.34(12)
N(3)-C(21)-C(22)	109.4(3)
N(3)-C(21)-H(21A)	109.8
C(22)-C(21)-H(21A)	109.8
N(3)-C(21)-H(21B)	109.8
C(22)-C(21)-H(21B)	109.8
H(21A)-C(21)-H(21B)	108.2
O(2)-C(22)-C(21)	113.2(4)
O(2)-C(22)-H(22A)	108.9
C(21)-C(22)-H(22A)	108.9
O(2)-C(22)-H(22B)	108.9
C(21)-C(22)-H(22B)	108.9
H(22A)-C(22)-H(22B)	107.8
N(3)-C(23)-C(24)	110.3(3)
N(3)-C(23)-H(23A)	109.6
C(24)-C(23)-H(23A)	109.6
N(3)-C(23)-H(23B)	109.6
C(24)-C(23)-H(23B)	109.6
H(23A)-C(23)-H(23B)	108.1
O(2)-C(24)-C(23)	111.8(4)

O(2)-C(24)-H(24A)	109.2
C(23)-C(24)-H(24A)	109.2
O(2)-C(24)-H(24B)	109.2
C(23)-C(24)-H(24B)	109.2
H(24A)-C(24)-H(24B)	107.9
C(13)-N(3)-C(21)	126.1(3)
C(13)-N(3)-C(23)	121.0(3)
C(21)-N(3)-C(23)	112.8(3)
C(24)-O(2)-C(22)	110.7(4)
N(3')-C(21')-C(22')	108.0(13)
N(3')-C(21')-H(21C)	110.1
C(22')-C(21')-H(21C)	110.1
N(3')-C(21')-H(21D)	110.1
C(22')-C(21')-H(21D)	110.1
H(21C)-C(21')-H(21D)	108.4
O(2')-C(22')-C(21')	113.8(14)
O(2')-C(22')-H(22C)	108.8
C(21')-C(22')-H(22C)	108.8
O(2')-C(22')-H(22D)	108.8
C(21')-C(22')-H(22D)	108.8
H(22C)-C(22')-H(22D)	107.7
C(24')-C(23')-N(3')	108.3(13)
C(24')-C(23')-H(23C)	110.0
N(3')-C(23')-H(23C)	110.0
C(24')-C(23')-H(23D)	110.0
N(3')-C(23')-H(23D)	110.0

H(23C)-C(23')-H(23D)	108.4
O(2')-C(24')-C(23')	115.8(14)
O(2')-C(24')-H(24C)	108.3
C(23')-C(24')-H(24C)	108.3
O(2')-C(24')-H(24D)	108.3
C(23')-C(24')-H(24D)	108.3
H(24C)-C(24')-H(24D)	107.4
C(21')-N(3')-C(23')	109.7(12)
C(24')-O(2')-C(22')	114.8(14)

Symmetry transformations used to generate equivalent atoms:

Table 4. Anisotropic displacement parameters ($Å^2 x 10^3$) for mo_150629_0m_a. The anisotropic displacement factor exponent takes the form: $-2\pi^2[h^2 a^{*2}U^{11} + ... + 2 h k a^* b^* U^{12}]$

	U^{11}	U^{22}	U^{33}	U^{23}	U^{13}	U^{12}
C(1)	44(1)	61(2)	43(1)	3(1)	10(1)	-2(1)
C(2)	56(2)	69(2)	72(2)	14(2)	14(1)	-6(2)
C(3)	67(2)	80(2)	84(2)	28(2)	15(2)	12(2)
C(4)	46(2)	109(3)	80(2)	27(2)	14(2)	9(2)
C(5)	45(2)	103(3)	79(2)	24(2)	17(1)	-5(2)
C(6)	48(1)	74(2)	64(2)	17(2)	16(1)	-3(1)
C(7)	42(1)	56(2)	47(1)	1(1)	11(1)	-8(1)
C(8)	46(1)	58(2)	55(2)	9(1)	18(1)	-2(1)
C(9)	48(1)	53(2)	44(1)	2(1)	13(1)	-1(1)
C(10)	46(1)	53(1)	46(1)	-2(1)	18(1)	3(1)
C(11)	53(2)	54(2)	58(2)	-5(1)	21(1)	-2(1)
C(12)	63(2)	47(1)	71(2)	2(1)	27(2)	4(1)
C(13)	46(1)	60(2)	62(2)	-4(1)	17(1)	13(1)
C(14)	48(1)	50(1)	50(1)	5(1)	15(1)	5(1)
C(15)	46(1)	49(1)	47(1)	-1(1)	18(1)	9(1)
C(16)	58(2)	49(2)	60(2)	4(1)	20(1)	14(1)
C(17)	46(1)	50(1)	48(1)	2(1)	16(1)	13(1)
C(18)	50(1)	53(2)	54(2)	8(1)	14(1)	14(1)
C(19)	50(1)	53(2)	58(2)	2(1)	19(1)	8(1)
C(20)	45(1)	55(2)	54(2)	-4(1)	17(1)	12(1)
N(1)	46(1)	60(1)	66(1)	19(1)	8(1)	-4(1)

N(2)	44(1)	59(1)	61(1)	11(1)	9(1)	-3(1)
O(1)	49(1)	64(1)	104(2)	-14(1)	22(1)	16(1)
S(1)	48(1)	49(1)	66(1)	6(1)	12(1)	14(1)
C(21)	60(2)	64(2)	70(2)	-10(2)	18(2)	15(2)
C(22)	86(2)	120(3)	73(3)	-32(2)	18(2)	24(3)
C(23)	49(2)	86(3)	66(2)	-8(2)	12(2)	9(2)
C(24)	73(2)	108(3)	66(2)	-10(2)	0(2)	18(2)
N(3)	45(1)	64(2)	53(2)	-4(1)	14(1)	13(1)
O(2)	92(2)	131(3)	74(2)	-38(2)	-2(2)	20(2)
C(21')	63(4)	88(5)	65(5)	-18(4)	17(4)	20(4)
C(22')	77(4)	98(5)	70(5)	-17(4)	13(5)	13(4)
C(23')	53(4)	93(4)	66(5)	-9(4)	5(4)	15(4)
C(24')	69(4)	107(5)	74(5)	-14(4)	7(4)	9(4)
N(3')	50(4)	80(4)	63(4)	-10(4)	12(4)	19(4)
O(2')	79(4)	107(5)	71(5)	-18(4)	7(4)	7(4)

Table 5. Hydrogen coordinates (x 10^4) and isotropic displacement parameters ($Å^2$x 10^3) for mo_150629_0m_a.

	x	y	z	U(eq)
H(2)	5937	-1466	9199	81
H(3)	6805	-2617	9591	96
H(4)	7494	-355	9588	97
H(5)	7312	3079	9183	92
H(6)	6451	4230	8789	75
H(8)	5558	5121	8258	64
H(11)	4843	8170	7930	66
H(12)	4193	10131	7280	71
H(14)	3822	3173	7782	60
H(16)	3363	8644	6877	67
H(18)	3046	1703	7398	64
H(19)	2112	961	6905	65
H(1)	5193	82	9018	72
H(21A)	1328	-598	5983	79
H(21B)	1813	861	5958	79
H(22A)	1371	1772	5072	115
H(22B)	1366	-843	5123	115
H(23A)	483	4161	5810	82
H(23B)	457	1552	5875	82
H(24A)	75	2436	4974	106

H(24B)	590	3759	4983	106
H(21C)	1865	1649	5721	88
H(21D)	1448	2252	5143	88
H(22C)	1348	-1428	5327	102
H(22D)	1281	-789	5885	102
H(23C)	520	4873	5614	90
H(23D)	642	4298	5077	90
H(24C)	395	1278	5771	106
H(24D)	53	1739	5158	106
